# Comparing antibody assays as correlates of protection against COVID-19 in the COVE mRNA-1273 vaccine efficacy trial

**DOI:** 10.1126/scitranslmed.ade9078

**Published:** 2023-04-19

**Authors:** David Benkeser, David C. Montefiori, Adrian B. McDermott, Youyi Fong, Holly E. Janes, Weiping Deng, Honghong Zhou, Christopher R. Houchens, Karen Martins, Lakshmi Jayashankar, Flora Castellino, Britta Flach, Bob C. Lin, Sarah O’Connell, Charlene McDanal, Amanda Eaton, Marcella Sarzotti-Kelsoe, Yiwen Lu, Chenchen Yu, Bhavesh Borate, Lars W. P. van der Laan, Nima S. Hejazi, Avi Kenny, Marco Carone, Brian D. Williamson, Jennifer Garver, Erin Altonen, Thomas Rudge, Chuong Huynh, Jacqueline Miller, Hana M. El Sahly, Lindsey R. Baden, Sharon Frey, Elissa Malkin, Stephen A. Spector, Michele P. Andrasik, James G. Kublin, Lawrence Corey, Kathleen M. Neuzil, Lindsay N. Carpp, Rolando Pajon, Dean Follmann, Ruben O. Donis, Richard A. Koup, Peter B. Gilbert, on behalf of the Immune Assays, Moderna Inc., Coronavirus Vaccine Prevention Network (CoVPN)/Coronavirus Efficacy (COVE), United States Government (USG)/CoVPN Biostatistics Teams

**Affiliations:** 1 Department of Biostatistics and Bioinformatics, Rollins School of Public Health, Emory University, Atlanta, GA 30322, USA; 2 Department of Surgery and Duke Human Vaccine Institute, Duke University Medical Center, Durham, NC 27710, USA; 3 Vaccine Research Center, National Institute of Allergy and Infectious Diseases, National Institutes of Health, Bethesda, MD 20892, USA; 4 Vaccine and Infectious Disease Division, Fred Hutchinson Cancer Center, Seattle, WA 98109, USA; 5 Public Health Sciences Division, Fred Hutchinson Cancer Center, Seattle, WA 98109, USA; 6 Moderna Inc., Cambridge, MA 02139, USA; 7 Biomedical Advanced Research and Development Authority, Washington, DC 20201, USA; 8 Department of Biostatistics, T.H. Chan School of Public Health, Harvard University, Boston, MA 02115, USA; 9 Department of Biostatistics, University of Washington, Seattle, WA 98195, USA.; 10 Kaiser Permanente Washington Health Research Institute, Seattle, WA 98101, USA; 11 Battelle, West Jefferson, OH 43162, USA; 12 Baylor College of Medicine, Houston, TX 77030, USA; 13 Brigham and Women’s Hospital, Boston, MA 02115, USA; 14 Department of Internal Medicine, Saint Louis University, St. Louis, MO 63110, USA; 15 Vaccine Research Unit, School of Medicine and Health Sciences, George Washington University, Washington, DC 20052, USA; 16 Division of Pediatric Infectious Diseases, University of California, San Diego, La Jolla, CA 92093, USA; 17 Rady Children’s Hospital, San Diego, CA 92123, USA; 18 Department of Laboratory Medicine and Pathology, University of Washington, Seattle, WA 98115, USA; 19 Center for Vaccine Development and Global Health, University of Maryland School of Medicine, Baltimore, MD 21201, USA; 20 Biostatistics Research Branch, National Institute of Allergy and Infectious Diseases, National Institutes of Health, Bethesda, MD 20892, USA

## Abstract

The best assay or marker to define mRNA-1273 vaccine–induced antibodies as a correlate of protection (CoP) is unclear. In the COVE trial, participants received two doses of the mRNA-1273 COVID-19 vaccine or placebo. We previously assessed IgG binding antibodies to the spike protein (spike IgG) or receptor binding domain (RBD IgG) and pseudovirus neutralizing antibody 50 or 80% inhibitory dilution titer measured on day 29 or day 57, as correlates of risk (CoRs) and CoPs against symptomatic COVID-19 over 4 months after dose. Here, we assessed a new marker, live virus 50% microneutralization titer (LV-MN_50_), and compared and combined markers in multivariable analyses. LV-MN_50_ was an inverse CoR, with a hazard ratio of 0.39 (95% confidence interval, 0.19 to 0.83) at day 29 and 0.51 (95% confidence interval, 0.25 to 1.04) at day 57 per 10-fold increase. In multivariable analyses, pseudovirus neutralization titers and anti-spike binding antibodies performed best as CoRs; combining antibody markers did not improve correlates. Pseudovirus neutralization titer was the strongest independent correlate in a multivariable model. Overall, these results supported pseudovirus neutralizing and binding antibody assays as CoRs and CoPs, with the live virus assay as a weaker correlate in this sample set. Day 29 markers performed as well as day 57 markers as CoPs, which could accelerate immunogenicity and immunobridging studies.

## INTRODUCTION

The identification and validation of a correlate of protection (CoP), an immune biomarker that can be used to reliably predict the degree of vaccine efficacy against a clinically relevant outcome (1–3), is a priority in coronavirus disease 2019 (COVID-19) vaccine research (4, 5). CoPs are valuable for expediting vaccine development and use. For example, for a vaccine with established efficacy, a CoP could serve as a primary endpoint for immunobridging of vaccine efficacy to a target population that was not included in the randomized trial(s) that demonstrated efficacy or support approval of alternate vaccine regimens (e.g., modified schedule, dose, or variant viral strains). Common CoPs for licensed vaccines are measurements of binding antibodies (bAbs) or neutralizing antibodies (nAbs) (2), and multiple lines of investigation (6–12) have supported these immune markers as CoPs for COVID-19 vaccines.

Immune correlate analyses of randomized phase 3 trials provide particularly valuable evidence to support an immune biomarker as a CoP. In the Coronavirus Efficacy (COVE) phase 3 trial of the mRNA-1273 vaccine (NCT04470427), conducted at 99 clinical sites in the United States, 30,420 participants were randomized at a 1:1 ratio to receive mRNA-1273 vaccine or placebo. Injections were administered on day 1 (D1) and D29, with all participants receiving their first trial injection between 27 July and 23 October 2020. Efficacy of the mRNA-1273 vaccine in the blinded phase (median follow-up, 5.3 months) was 93.2% [95% confidence interval (CI), 91.0 to 94.8%] against symptomatic, virologically confirmed COVID-19 starting ≥14 days after D29 (13). We recently reported that immunoglobulin G (IgG) bAbs against the spike protein (spike IgG), IgG bAbs against the spike receptor binding domain (RBD IgG), 50% inhibitory dilution pseudovirus-nAb (PsV-nAb ID_50_) titer, and 80% inhibitory dilution PsV-nAb (PsV-nAb ID_80_) titer all correlated inversely with symptomatic, virologically confirmed COVID-19 (hereafter, “primary COVID-19 endpoint”) in two-dose vaccine recipients. Furthermore, these features were associated with mRNA-1273 vaccine efficacy against the primary COVID-19 endpoint through 4 months after D29 (10). These findings held whether the antibody markers were measured at D29 (1 month after first dose) or at D57 (1 month after second dose).

The present analysis had three objectives. First, we assessed nAbs measured using a live virus 50% microneutralization assay (LV-MN_50_), which were not assessed previously (10), as a correlate of risk (CoR) and as a CoP (14) against the primary COVID-19 endpoint in the COVE trial using the same clinical data previously analyzed (10) and using the same and additional statistical methods. Second, we synthesized the evidence supporting each of the 10 markers [the four markers from (10) and the LV-MN_50_ marker from this work, each measured at two time points] as immune correlates and ranked their performance. Last, we performed machine learning analyses evaluating multivariable CoRs of COVID-19 by studying how to best predict occurrence of the primary COVID-19 endpoint among vaccine recipients on the basis of the five immune assays and both sampling time points. This analysis provides comparisons of prediction performance across the individual markers and addresses whether combining multiple markers improves prediction of COVID-19. All markers measured antibodies against the vaccine strain or against the dominant circulating strain at the time, D614G, both in the ancestral lineage; severe acute respiratory syndrome coronavirus 2 (SARS-CoV-2) strains circulating during trial follow-up were all of the ancestral lineage or of slightly genetically drifted lineages (15). Therefore, this study essentially evaluated homologous antibody responses as immune correlates.

## RESULTS

### Immunogenicity subcohort, case-cohort sets, and COVID-19 endpoints

The demographic and clinical characteristics of participants in the randomly sampled immunogenicity subcohort (1010 vaccine recipients and 137 placebo recipients), as well as participant flow from enrollment through inclusion in the D29 or D57 marker case-cohort set, have been described (10). The COVID-19 endpoint for the correlate analysis was the same as the COVID-19 endpoint for the primary efficacy analysis (13, 16) (primary COVID-19 endpoint): first occurrence of virologically confirmed symptomatic SARS-CoV-2 infection in participants with no evidence of previous SARS-CoV-2 infection. However, although the primary efficacy analysis counted COVID-19 endpoints starting 14 days after D29, the D29 marker correlate analyses counted vaccine breakthrough COVID-19 endpoints starting 7 days after D29 (*n* = 46; last endpoint occurred 126 days after D29), and the D57 marker correlate analyses counted vaccine breakthrough COVID-19 endpoints starting 7 days after D57 (*n* = 36; last endpoint occurred 100 days after D57) [figure S3 of (10)]. Seven days was chosen as the purported earliest time after D29 or D57 by which primary COVID-19 endpoints would not have their D29 or D57 antibody markers influenced by the SARS-CoV-2 infection causing the COVID-19 endpoint.

### Lower LV-MN_50_ titers were observed in vaccine cases versus non-cases

**Fig. 1.**
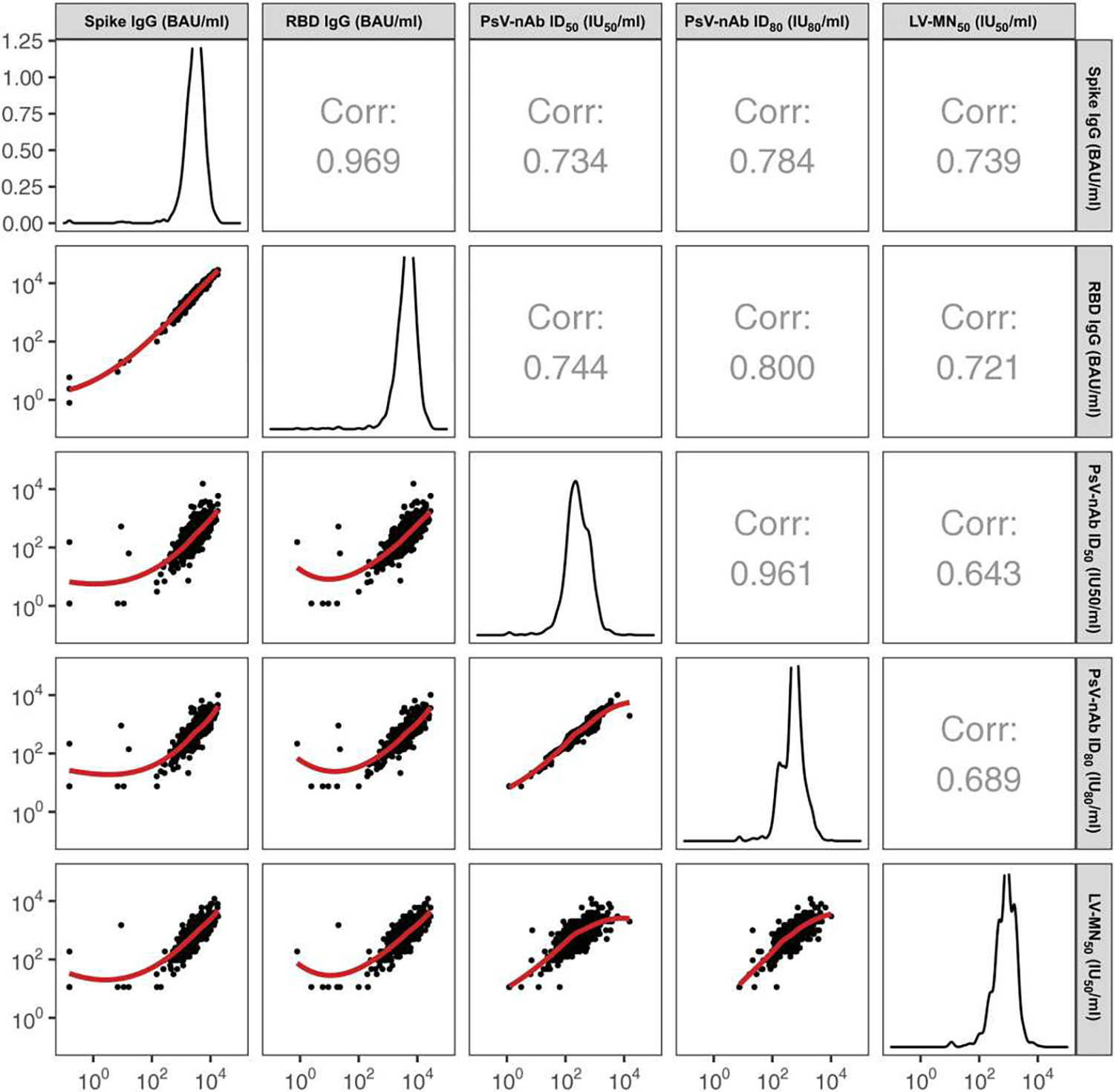
D57 LV-MN50 titers are more highly correlated with D57 spike IgG concentrations and with D57 RBD IgG concentrations than with D57 PsV-nAb ID50 titers or with D57 PsV-nAb ID80 titers. Analyses were conducted in baseline SARS-CoV-2–negative per-protocol vaccine recipients in the immunogenicity sub-cohort. Corr indicates the baseline variable-adjusted Spearman rank correlation. ID_50_, 50% inhibitory dilution; ID_80_, 80% inhibitory dilution; LV, live virus; MN_50_, 50% microneutralization dilution; nAb, neutralizing antibody; PsV, pseudovirus. Correlations among spike IgG, RBD IgG, PsV-nAb, ID_50_, and PsV-nAb ID_80_ were reported previously [figure S6 of (10)]. Serological assay readouts are expressed in values relative to the World Health Organization (WHO) International Standard for anti–SARS-CoV-2 immunoglobulin (27). bAb readouts were converted to bAb units per milliliter (BAU/ml), and PsV-nAb titers and microneutralization assay readouts were calibrated to international units per milliliter (IU_50_/ml or IU_80_/ml).

LV-MN_50_ nAb titers were detectable in 69.2% (95% CI: 65.8, 72.4%) of vaccine recipient non-cases at D29 and 99.3% (98.3, 99.7%) of vaccine recipient non-cases at D57 (Table 1; table S1 provides the numbers of participants with antibody markers measured at D29 and D57). D57 LV-MN_50_ was highly correlated with both D57 spike IgG and D57 RBD IgG (Spearman rank correlations *r* = 0.74 and 0.72, respectively) (Fig. 1). D29 LV-MN_50_ showed correlations of similar strength with each of the other D29 markers (all *r* > 0.74; fig. S1). The D57 LV-MN_50_ and D57 PsV-nAb ID_50_ assay measurements were less correlated [*r* = 0.64 (0.60, 0.68)] (Fig. 1). D29 LV-MN_50_ and D57 LV-MN_50_ titers were weakly correlated [*r* = 0.47 (0.42, 0.52)] (fig. S2).

**Fig. 2.**
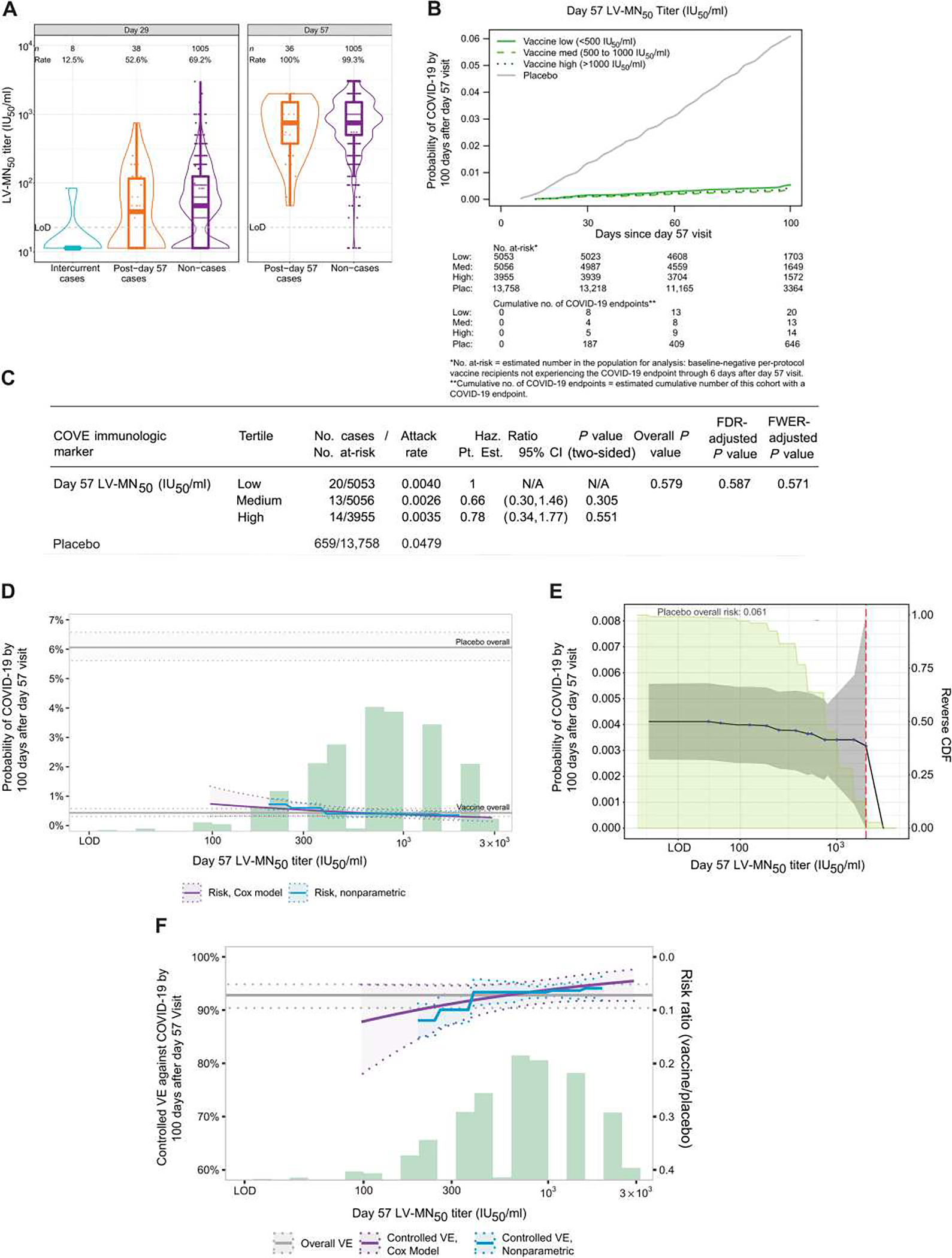
Correlate analyses show limited evidence for D57 LV-MN50 titer as a CoR and as a CoP. (**A**) LV-MN_50_ titers are shown binned by COVID-19 outcome status in baseline SARS-CoV-2–negative per-protocol vaccine recipients. Each sample was tested independently in singlet by one operator on one test plate following the standard operator procedures. The same sample was then tested by a second operator in singlet on a different plate on the same day. If necessary, repeat testing of any samples was performed in singlet by one operator on a different test day. The final reportable value for each sample was the median LV-MN_50_ titer of a minimum of two passing independent results. Data points are from the D29 marker or D57 marker case-cohort set. The violin plots contain interior box plots with upper and lower horizontal edges being the 25th and 75th percentiles of antibody concentrations, respectively, and the middle line being the 50th percentile; the vertical bars show the distance from the 25th (or 75th) percentile of antibody concentration and the minimum (or maximum) antibody concentration within the 25th (or 75th) percentile of antibody concentration minus (or plus) 1.5 times the interquartile range. Each side shows a rotated probability density (estimated by a kernel density estimator with a default Gaussian kernel) of the data. Positive response rates were computed with inverse probability of sampling weighting. Positive response was defined by value > LoD (22.66 IU_50_/ml). Post-D57 cases are COVID-19 endpoints starting 7 days after D57 through the end of blinded follow-up (last COVID-19 endpoint 126 days after dose 2); intercurrent cases are COVID-19 endpoints starting 7 days after D29 through 6 days after D57. IU, international units; LoD, limit of detection. (**B**) Shown is the cumulative incidence of COVID-19 for the low, medium, and high tertiles of D57 LV-MN_50_ titers. (**C**) Shown are the estimated hazard ratios of COVID-19 for the medium versus low and for the high versus low tertiles of D57 LV-MN_50_. Both comparisons were made in baseline SARS-CoV-2–negative per-protocol participants. All *P* values are based on Wald tests; multiplicity adjustments are shown controlling false discovery rate and FWER over the set of *P* values (separately for D29 and for D57 marker CoRs) both for quantitative markers and categorical markers (considering all five antibody markers: spike IgG, RBD IgG, PsV-nAb ID_50_, PsV-nAb ID_80_, and LV-MN_50_) using the Westfall and Young (34) permutation method (10,000 replicates). The overall *P* value is from a generalized Wald test of whether the hazard rate of COVID-19 differed across the low, medium, and high subgroups. N/A, not applicable. (**D**) Shown is the cumulative incidence of COVID-19 by 100 days after D57 by D57 LV-MN_50_ titer, estimated using (solid purple line) a Cox model or (solid blue line) a nonparametric model. Purple dotted lines indicate the bootstrap point-wise 95% CIs; blue dotted lines indicate the influence function–based Wald-based 95% confidence intervals (CIs). Upper and lower horizontal gray lines indicate overall cumulative incidence of COVID-19 from 7 to 100 days after D57 in placebo and vaccine recipients, respectively. The green histogram indicates the frequency distribution of D57 marker among baseline SARS-CoV-2–negative per-protocol vaccine recipients. (**E**) Shown is the cumulative incidence of COVID-19 by 100 days after D57 by D57 LV-MN_50_titer PsV-nAb ID_50_ titer above a threshold [versus at a specific threshold, as in (D)]. Blue dots indicate point estimates at each COVID-19 primary endpoint linearly interpolated by solid black lines; gray shading indicates point-wise 95% CIs. The estimates and CIs assume a nonincreasing threshold-response function. The upper boundary of the green shaded area indicates the estimate of the reverse cumulative distribution function (CDF) of D57 LV-MN_50_ concentrations. The vertical red dashed line indicates the D57 LV-MN_50_ occurred (in the time frame of 7 days after D57 through to the data cutoff date of 26 March 2021). (**F**) Shown is vaccine efficacy by D57 LV-MN_50_ titer estimated by different implementations of (30). The solid purple line indicates vaccine efficacy by D57 LV-MN_50_ titer, estimated using a Cox proportional hazard implementation of (30); dotted purple lines indicate bootstrap point-wise 95% CIs. The solid blue line indicates vaccine efficacy by D57 LV-MN_50_ titer, estimated using a nonparametric implementation of (30); dotted blue lines indicate 95% CIs. The green histogram indicates the frequency distribution of D57 marker among baseline SARS-CoV-2–negative per-protocol vaccine recipients. The horizontal gray line indicates overall vaccine efficacy from 7 to 100 days after D57, and the dotted gray lines indicates 95% CIs. Analyses were adjusted for baseline risk score, at-risk status, and community of color status. Microneutralization assay readouts were calibrated to the WHO anti–SARS-CoV-2 immunoglobulin International Standard (27) and are expressed in IU_50_/ml.

Geometric mean LV-MN_50_ nAb titers were lower in vaccine recipient cases versus non-cases at D29 [31.4 international units, 50% inhibitory dose/ml (IU_50_/ml) (95% CI: 22.0, 45.0) versus 48.4 IU_50_/ml (44.6, 52.6); cases:non-cases ratio = 0.65 (0.45, 0.94)]. The estimated difference was smaller for D57, and CIs for the geometric mean ratio crossed 1.0 [594 IU_50_/ml (433 and 816) in cases versus 718 IU_50_/ml (676 and 763) in non-cases, cases:non-cases ratio = 0.83 (0.60, 1.14)] (Table 1). Figure 2A shows the distributions of D29 and D57 LV-MN_50_ nAb titers in vaccine recipient cases and non-cases. Seven of the eight (87.5%) intercurrent cases, defined as COVID-19 endpoints occurring between 7 days after D29 and 6 days after D57, had D29 LV-MN_50_ titers below the assay’s limit of detection compared with 30.8% of non-cases. In contrast, all post-D57 cases had detectable D57 LV-MN_50_ titers (similar to the 99.3% of non-cases with detectable D57 LV-MN_50_ titers). There were low frequencies of placebo recipients with LV-MN_50_ above the assay’s limit of detection (e.g., at D57, 1.5% in non-cases and 0.2% in cases) (fig. S3); the other assays also had frequencies near zero (10). The reverse cumulative distribution curves of D29 and of D57 LV-MN_50_ and overall vaccine efficacy estimates are shown in fig. S4.

### CoR analysis of LV-MN_50_ using inverse probability sampling–weighted Cox regression

The Cox model–based COVID-19 cumulative incidence curves for vaccine recipient subgroups, defined by D57 LV-MN_50_ tertile, show that point estimates of COVID-19 risk decreased as tertile increased, with a hazard ratio for the medium versus low D57 LV-MN_50_ tertile of 0.66 (95% CI: 0.30, 1.46; *P* = 0.31) and for high versus low D57 LV-MN_50_ tertile of 0.78 (95% CI: 0.34, 1.77; *P* = 0.55) (Fig. 2, B and C). The wide CIs for the two hazard ratios suggest a lack of precision and no statistical evidence for a correlation (*P* = 0.58, Fig. 2C). For quantitative D57 LV-MN_50_, the estimated hazard ratio per 10-fold increase (95% CI) was 0.51 (0.25, 1.04; *P* = 0.065) (Table 2). For prespecified vaccine recipient subgroups, point estimates of hazard ratios per 10-fold increase of D57 LV-MN_50_ ranged from 0.37 (95% CI: 0.14, 0.96) to 0.73 (0.22, 2.46) (fig. S5), with most of the CIs including one.

D29 LV-MN_50_ had stronger evidence as an inverse CoR than D57 LV-MN_50_, where both the family-wise error rate (FWER)–adjusted *P* value for the quantitative marker and for the marker in tertiles passed multiplicity correction (FWER-adjusted *P* = 0.017 and 0.021, respectively) (Table 2 and fig. S6). The hazard ratio per 10-fold D29 LV-MN_50_ increment was 0.39 (0.19, 0.83), the hazard ratio for the medium versus low tertile was 0.37 (0.17, 0.82), and the hazard ratio for the high versus low tertile was 0.46 (0.21, 1.01). Cox modeling analyses estimating cumulative incidence for subgroups of vaccine recipients with D57 LV-MN_50_ titers at a given value also showed that increasing D57 LV-MN_50_ titer was associated with decreased COVID-19 cumulative incidence, with estimates of 0.0073 (95% CI: 0.0032, 0.013) at 100 IU_50_/ml, 0.0046 (0.0031, 0.0062) at 500 IU_50_/ml, and 0.0031 (0.0017, 0.0040) at 2000 IU_50_/ml, an ~2.5-fold difference in risk across these values (Fig. 2D).

### CoR analysis of LV-MN_50_ using nonparametric targeted minimum loss–based threshold regression

Nonparametric threshold regression analyses estimating cumulative incidence for subgroups of vaccine recipients with D57 LV-MN_50_ titers above a given threshold value showed a mild decrease in cumulative incidence as D57 LV-MN_50_ titer threshold increased. The estimates were 0.0041 (95% CI: 0.0026, 0.0056), 0.0036 (0.0020, 0.0052), and 0.0032 (0.00, 0.0093) at D57 LV-MN_50_ titer thresholds of undetectable (<22.66 IU_50_/ml), 500 IU_50_/ml, and 2000 IU_50_/ml, respectively (Fig. 2E). This decrease was less for D29 LV-MN_50_ (fig. S7).

### CoP analysis of LV-MN_50_ using Cox proportional hazards estimation and nonparametric monotone dose-response estimation of controlled vaccine efficacy

Vaccine efficacy point estimates rose as D57 LV-MN_50_ titer increased (Fig. 2F). At the D57 LV-MN_50_ titer of 100 IU_50_/ml, the estimated vaccine efficacy was 87.9% (95% CI: 78.2, 94.7%); this increased to 92.4% (89.7, 94.8%) at 500 IU_50_/ml and to 94.9% (92.0, 97.2%) at 2000 IU_50_/ml (purple curve). Similar results were seen with nonparametric estimation of the vaccine efficacy–by–D57 LV-MN_50_ curve (blue line, Fig. 2F). Analogous curves of vaccine efficacy–by–D29 LV-MN_50_ titer were similar, with slightly greater increase in estimated vaccine efficacy with titer (fig. S8). Using a sensitivity analysis that assumed the existence of unmeasured confounding that would make it harder for vaccine efficacy to increase with titer, estimated vaccine efficacy still increased (albeit to a lesser extent) with increasing D57 LV-MN_50_ titer (fig. S9).

### CoP analysis of LV-MN_50_ using mediation analysis of vaccine efficacy

The method by Benkeser *et al.* (17) was used to assess D29 LV-MN_50_ titeras a mediator of vaccine efficacy, which identifies the fraction of total risk reduction conferred by vaccination that can be attributed to the given marker. An estimated 29.2% (95% CI: 17.2, 41.2%) of vaccine efficacy was mediated by D29 LV-MN_50_ titer (Table 3). The D57 nAb markers could not be assessed as mediators of vaccine efficacy, because detectable response rates in vaccine recipients exceeded 98%. Thus, there was not enough overlap between marker values in placebo and vaccine recipients to perform the analysis.

### Comparison of LV-MN_50_ and PsV-nAb ID_50_ titers as CoRs and as CoPs

On the basis of the above analyses of the LV-MN_50_ markers and the same analyses of the PsV-nAb markers (10), we compared performance of the two assays as CoRs and as CoPs. The readouts of the two assays can be directly compared because they are expressed in the same units (IU_50_/ml) based on calibration to the World Health Organization anti–SARS-CoV-2 immunoglobulin International Standard. Table 2, table S2, and figs. S3 to S13 provide side-by-side comparisons of the LV-MN_50_ and PsV-nAb ID_50_ results. Overall, the evidence in support of PsV-nAb ID_50_ titer as a CoR and as a CoP was stronger than that in support of LV-MN_50_ titer for both the D29 and D57 markers. In addition, as noted above, an estimated 29.2% (95% CI: 17.2, 41.2%) of vaccine efficacy was mediated by D29 LV-MN_50_ titer; in contrast an estimated 68.5% (58.5, 78.4%) of vaccine efficacy was mediated by D29 PsV-nAb ID_50_ titer (10). Moreover, the estimated proportion of vaccine efficacy mediated through D29 PsV-nAb ID_50_ titer alone was similar to that mediated through both D29 neutralization markers analyzed together [62.9% (52.9, 72.8%)], supporting the lack of incremental value in adding a live virus measurement to the PsV measurement.

### Ranking the individual immune markers based on CoR and CoP criteria

We next systematically compared the immune correlates performance of all five antibody markers at D57 and then repeated this comparison for the five markers at D29. We then conducted the ranking combining all markers and both time points, and, lastly, we compared performance of each antibody marker at D29 versus at D57. We ranked by three categories of correlate-quality criteria: (1) risk prediction or strength of association of an immune marker with COVID-19 in vaccine recipients [four criteria: (i) point estimate of hazard ratio per SD increment, (ii) *P* value for hazard ratio departing from unity, (iii) point estimate of hazard ratio high versus low tertile, and (iv) *P* value for hazard ratio departing from unity]; (2) extent of vaccine efficacy modification by an immune marker [three criteria: (i) span of the point estimate of vaccine efficacy from the 5th to 95th percentile of the marker as obtained by the marginalized Cox model, (ii) span of the point estimate of vaccine efficacy from the 5th to 95th percentile of the marker as obtained by nonparametric estimation, and (iii) upper 95% confidence limit of the *E* value for the marginalized Cox model (high versus low)]; and (3) extent of the vaccine efficacy that is mediated through an immune marker [two criteria: (i) point estimate and (ii) lower 95% confidence limit of proportion of vaccine efficacy mediated through the immune marker (when these were available)].

For the D57 markers, PsV-nAb ID_80_ ranked highest in both evaluable categories (Table 4). The greatest difference in assay performance was between the bAb and PsV-nAb assays versus the live virus neutralization assay. For the D29 markers, pike IgG ranked highest in category 1, whereas PsV-nAb ID_50_ ranked highest in categories 2 and 3 (Table 4). Similar to the D57 results, the D29 LV-MN_50_ marker ranked below both bAb markers and both PsV-nAb assay markers in all three categories.

When ranking performance across all assay readouts and across both the D57 and D29 time points, D29 spike IgG, D29 PsV-nAb ID_80_, and D29 PsV-nAb ID_50_ performed best across categories 1, 2, and 3, respectively (table S3). When comparing within each D29 and D57 antibody marker pair for a given immune assay readout, the D29 marker had higher median ranks in both categories 1 and 2 for four of the five immune assay readouts. Spike IgG is the only exception where the D29 versus D57 comparison did not yield consistent results across both categories: D29 spike IgG ranked higher than D57 spike IgG in category 1, whereas the opposite was true in category 2.

### Comparison of all pairs of individual markers in terms of their standardized association with COVID-19 risk

After an immune marker is accepted as a CoP for a certain vaccine, it typically will be used as a primary endpoint in an immunobridging study for comparing the geometric mean marker value between a new condition and a standard condition. Therefore, a criterion for comparing the quality of two accepted CoPs is the ratio of sample sizes required to power the future immunobridging study for comparing the geometric mean between the two randomized study arms. For all pairs of the five markers at each time point, Follmann’s method (18) was applied to calculate this sample size ratio, with a marginalized Cox model implementation (see Materials and Methods). Analyses were performed separately for D29 and D57, because the method does not provide an approach for comparing the markers across time points. For the D57 markers, PsV-nAb ID_80_ requires the smallest sample size to detect the same geometric mean ratio effect size (0.94 times that of PsV-nAb ID_50_, 0.58 times that of spike IgG, and 0.23 times that of LV-MN_50_) (table S4). In addition, RBD IgG was slightly more efficient than spike IgG (0.85 times less sample size). For the D29 markers, spike IgG requires the smallest sample size (0.90 times that of RBD IgG, 0.61 times that of PsV-nAb ID_80_, and 0.41 times that of LV-MN_50_) (table S5). In addition, PsV-nAb ID_80_ was slightly more efficient than PsV-nAb ID_50_ (0.94 times less sample size). LV-MN_50_ would require between a 2.3 and 4.0 times–greater sample size than the other four markers.

### Sensitivity analysis for D29 markers

Stronger evidence for D29 markers may be anticipated, given that individuals with low D29 antibody markers may be at high risk for symptomatic COVID-19 before D57. Accordingly, these high-risk individuals would be included in the analysis of the D29 markers but not the D57 analysis. However, in a setting with lower transmission, there may be fewer such high-risk individuals, and, as such, D29 correlates may not generalize as well to these settings. To study this point, we included a sensitivity analysis that studied the D29 markers and their association with COVID-19 occurring more than 7 days after D57—the identical set of COVID-19 endpoints used in the analysis of the D57 markers. Restricting to post-D57 endpoints attenuated hazard ratio–associated D29 markers, resulting in hazard ratios similar to the D57 markers (table S6).

### Multivariable CoR analysis: Cox proportional hazards models

**Fig. 3.**
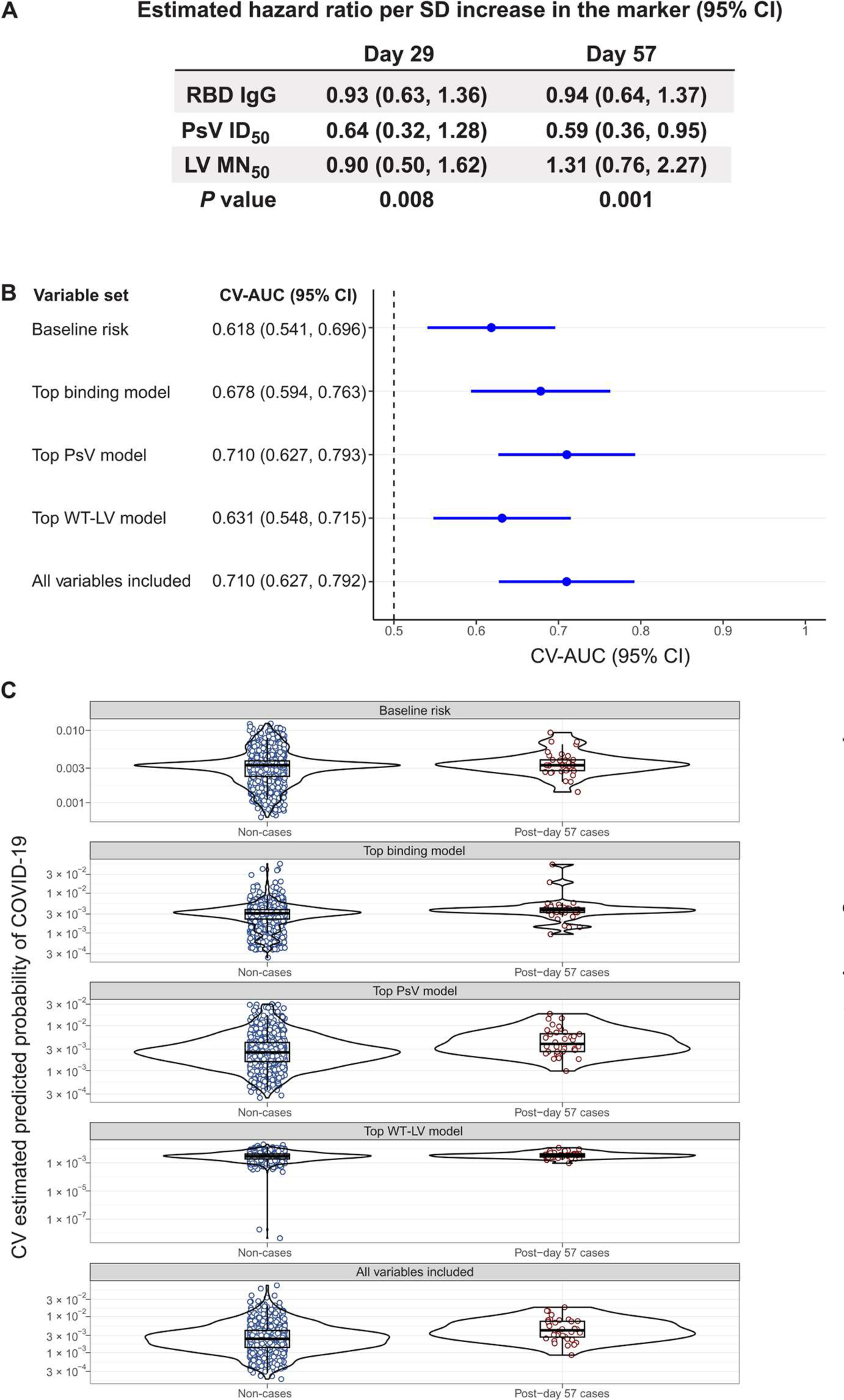
Multivariable modeling of COVID-19 risk shows that PsV neutralization titers and anti-spike bAbs perform best as CoRs, with no improvement in performance by including multiple markers. (**A**) Shown is the estimated COVID-19 hazard ratio per SD increase of the indicated antibody marker value in baseline SARS-CoV-2–negative per-protocol vaccine recipients. Hazard ratio was assessed using multivariable models. The two-phase sampling Cox model was adjusted for baseline risk score, at-risk status, and community of color status. The *P* values are from a generalized Wald test of the null hypothesis that all assay marker variables have null association. (**B**) The forest plot shows discrete Super Learner performance (weighted CV-AUC with 95% CI) for baseline risk factors, the top binding model, the top PsV-nAb model, the top wild-type live virus (WT-LV)–nAb model, and the model including all marker variables. All models include the baseline risk factors. The top binding model includes D57 bAb spike IgG, the top PsV-nAb model includes D29 and D57 PsV-nAb ID_80_, and the top LV-MN_50_ model includes D57 LV-MN_50_. The dashed vertical line indicates a CV-AUC of 0.5 (the prediction performance achieved by random guessing). (**C**) Shown are the distributions of weighted CV-estimated prediction probabilities for post-D57 cases (*n* = 36) and non-cases (*n* = 1005) using discrete Super Learner for baseline risk factors, the top binding model, the top PsV-nAb model, the top WT-LV-MN_50_ model, and the model including all marker variables. Post-D57 cases are COVID-19 endpoints starting 7 days after D57 through the end of blinded follow-up (last COVID-19 endpoint 126 days after dose 2). Serological assay readouts assessed as immune correlates were first expressed in values relative to the WHO International Standard for anti–SARS-CoV-2 immunoglobulin (27). bAb readouts were converted to BAU/ml, and PsV-nAb titers and microneutralization assay readouts were calibrated to IU_50_/ml or IU_80_/ml. CV-AUC, cross-validated area under the receiver operating characteristic curve.

We next studied the antibody markers in the same model to investigate which markers are the strongest independent CoRs when also accounting for other markers. In a Cox proportional hazards model that included the three prespecified D57 markers—RBD IgG, PsV-nAb ID_50_, and LV-MN_50_—the estimated hazard ratio of COVID-19 per SD increase in D57 PsV-nAb ID_50_ was 0.59 (95% CI: 0.36, 0.95) compared with 0.94 (0.64, 1.37) for D57 RBD IgG and 1.31 (0.76 and 2.27) for D57 LV-MN_50_ (Fig. 3A). This result supports PsV-nAb ID_50_ as the best independent correlate, being the only marker associated with COVID-19 with all three markers in the model. A similar result was obtained for the corresponding D29 markers (Fig. 3A). Exploratory analyses that refit the Cox model with each pair of the three antibody markers also yielded consistent and robust evidence for PsV-nAb ID_50_ as an independent inverse CoR (table S7). An exploratory analysis that refit the Cox model to the three markers with D57 PsV-nAb ID_80_ swapped in for D57 PsV-nAb ID_50_ yielded hazard ratios of 0.48 (95% CI: 0.20, 1.14) for D57 PsV-nAb ID_80_, 0.94 (0.64 and 1.36) for D57 RBD IgG, and 1.08 (0.57, 2.05) for D57 LV-MN_50_ (generalized Wald test of all three markers, *P* = 0.017), again supporting PsV-nAb ID_80_ as a better correlate than PsV-nAb ID_50_.

### Multivariable CoR analysis for predicting COVID-19 occurrence

We next used ensemble machine learning [“stacking” (19, 20) using the Super Learner algorithm (21)] to investigate whether individual-level primary COVID-19 endpoint outcomes in mRNA-1273 vaccine recipients were best predicted by individual immune markers or combinations thereof by building predictive models with combinations spanning all five immune assays and both sampling time points. The metric used for comparing the classification accuracy of the different models was the point estimate and the 95% CI of the cross-validated area under the receiver operating characteristic (ROC) curve (CV-AUC) (22) for each model fit. The goal of this analysis was to assess how much antibody markers improved prediction of risk after accounting for baseline risk factors (at-risk status, community of color classification, and baseline risk score, adjusted for in all correlate analyses). Thus, all models included baseline risk factors, and the CV-AUC of 0.618 (95% CI: 0.541, 0.696), attained by the discrete Super Learner using baseline risk factors alone, was the benchmark against which improvement was assessed (Fig. 3B). In the top performing model that only considered baseline factors and the bAb markers, classification accuracy improved, with a CV-AUC of 0.678 (0.594, 0.763). D57 spike IgG in L1-penalized logistic regression was the only bAb variable included in this model (table S8). Classification accuracy improved when considering the PsV neutralization markers instead of the bAb markers, with top performing discrete Super Learner model CV-AUC = 0.710 (0.627, 0.793). The PsV neutralization variables in this model were D57 PsV-nAb ID_80_, D29 PsV-nAb ID^80^, and the indicator of whether D29 PsV-nAb ID_80_ increased at least twofold from baseline (table S8). Classification accuracy was lower, however, when considering baseline factors and the live virus microneutralization markers, with top performing discrete Super Learner model CV-AUC = 0.631 (0.548, 0.715). Including both binding and PsV neutralization markers did not further improve classification accuracy, with top performing discrete Super Learner model CV-AUC = 0.710 (0.627, 0.792), the same performance achieved with the top PsV-nAb model. The weighted CV-estimated prediction probabilities for the primary COVID-19 endpoint, obtained using discrete Super Learner, had descriptively the most separation between non-cases and cases for the top PsV-nAb model and for the model including all marker variables (Fig. 3C), consistent with the results above.

## DISCUSSION

For participants in the COVE trial with no evidence of previous SARS-CoV-2 infection at baseline and who received two doses of mRNA-1273 vaccine, LV-MN_50_ at D29 correlated inversely with risk, with multiple hypothesistesting adjustment indicating a significant association for this time point (FWER-adjusted *P* values for the quantitative marker and for the marker in tertiles, *P* = 0.017 and 0.021, respectively), whereas LV-MN_50_ at D57 had a weaker association that did not pass hypothesis testing adjustment. Correspondingly, vaccine efficacy against COVID-19 rose with increasing LV-MN_50_ titer, and, again, this relationship generally appeared stronger for the D29 than the D57 marker. D29 LV-MN_50_ titer was estimated to mediate a small proportion (29%) of the overall 92.3% vaccine efficacy.

Across all analyses, evidence for correlates was stronger for nAbs measured by the PsV-based versus live virus–based neutralization assay, consistent with the findings of a nonhuman primate challenge study (6). Prentice surrogate endpoint evaluation further supported this conclusion. However, an immune correlate analysis of the COV002 (U.K.) trial of the ChAdOx1 nCoV-19 (AZD1222) vaccine (9) reported that live virus neutralization titer measured 28 days after dose 2 was as good (or potentially even better) a correlate of AZD1222 protection against COVID-19 as lentiviral PsV neutralization titers. A potential determinant of these differences is the relative precision of these live virus assays, which was not reported.

Furthermore, in that analysis, estimated vaccine efficacy was near zero for vaccine recipients with undetectable live virus neutralization but was positive for vaccine recipients with undetectable PsV neutralization. Postvaccination PsV-nAb ID_50_ titers were lower in COV002 than those in COVE, with a median value of 22.6 IU_50_/ml (interquartile range: 11.6, 46.8 IU_50_/ml) measured 28 days after dose 2 in nucleic acid amplification test–negative controls in COV002 [table S2 of (9)] versus a median value of 254 IU_50_/ml (interquartile range: 148, 499 IU_50_/ml) measured 28 days after dose 2 in non-cases in the immunogenicity subcohort of COVE (10). Thus, the vaccine platform may influence the performance of live virus neutralization assay readouts as immune correlates.

The apparent limitation of the live virus neutralization assay may reflect the diversity of live virus assay designs. It may also reflect the replication capacity of SARS-CoV-2 in Vero-E6 cells derived from African green monkey epithelial cells. In contrast, the PsV assay was performed in human embryonic kidney 293 cells, a human cell line overexpressing angiotensin-converting enzyme–related carboxypeptidase (ACE2) (the primary cellular receptor for SARS-CoV-2). A live virus neutralization assay using human airway epithelial or lung epithelial cells may yield a better CoP. In addition, because the ancestral SARS-CoV-2 strain was used in the live virus neutralization assay, the use of a strain more closely representative of the circulating variant during the time of follow-up may also yield a better correlate. Consistent with this hypothesis, the D614G strain (used in the PsV neutralization assay) was the predominant variant during the trial. Another potential explanation is the greater technical variability in the live virus assay, such that correlate strength depends on assay precision (as also suggested by the better correlate with ID_80_ values versus ID_50_ values in the PsV neutralization assay, as discussed below). Another potential explanation for why the live virus–nAb measurement may be a weaker correlate compared with the PsV-nAb measurements is greater intrasample variability. However, the assay validation studies did not support this, with similar estimated percent coefficients of variation (total counting interoperator and intra-assay variation) of 42.7% for D57 LV-MN_50_ compared with 44.1% for D57 PsV-nAb ID_50_. However, the intervaccine recipient variance of the D57 LV-MN_50_ marker was lower than that of the D57 PsV-nAb ID_50_ marker (0.177 compared with 0.220), indicating a greater biologically relevant dynamic range for the PsV assay that improves its ability to perform as a CoP.

From the PsV assay, the ID_80_ titer readout performed better as a CoP than the ID_50_ titer readout, consistent with a recent finding for a HIV monoclonal antibody (23). Traditionally, where neutralization assays have been used as a CoP, ID_50_ titer has been used, because the readout results are from the center portion of the standard curve and have more stability from a repeatability perspective. ID_50_ has continually demonstrated to be a CoP, and it is anticipated that these results will continue to be used for immunobridging purposes. However, this finding motivates future research for vaccines to pursue improving the correlate by comparing performance of ID_80_ values versus ID_50_ values and studying other neutralization readouts that may further optimize the correlate. Another conclusion is that the antibody markers generally performed better as CoPs when measured 4 weeks after dose 1 (at dose 2) than when measured 4 weeks after dose 2. A potential explanation is a “ceiling effect” of the markers at D57, when many vaccine recipients had high antibody responses that reduced intervaccine recipient dynamic range compared with the markers at D29 (for example, fig. S10 shows wider variability of LV-MN_50_ titers at D29 than at D57). Another potential explanation is the lower dynamic range of the markers at D57 due to early COVID-19 endpoints occurring in individuals with low antibody responses before D57. Our sensitivityanalysis that removed these intercurrent COVID-19 endpoints showed attenuated estimates of the association of the immune markers with COVID-19. Nevertheless, the D29 markers generally retained estimated associations that were at least as strong as at D57. One hypothesis for why D29 markers retain such a strong association, despite not necessarily reflecting peak antibody activity, is that the D29 markers may reflect host factors that associate with improved vaccine immunity; for example, being a strong vaccine responder may be revealed more clearly at D29 after one dose and obscured more at D57 after two doses of the potent mRNA vaccine. In other words, there may be a maximum antibody response the body can make, and getting to that point more quickly could mark a stronger immune system. Underlying factors such as innate responses, B cell memory pools, and epitope breadth remain to be determined. Nevertheless, our results suggest that it may be feasible to define a CoP at a measurement time point before completion of the full immunization series, which would provide the practical advantage of accelerating immunogenicity and immunobridging studies. Given the possibility of a three-dose primary immunization series for naïve populations such as young children, this finding may have implications for more efficiently predicting the efficacy of such an immunization series. Moreover, analysis of the sample size ratio required for powering a future immunogenicity or immunobridging study estimated that PsV-nAb ID_80_ was more efficient than spike IgG when measured at D57; however, the opposite was true when measured at D29, where ID_80_ failed to detect weak responses that scored as positive using the less stringent ID_50_. Further analyses would be needed to definitively determine whether spike IgG is a particularly efficient or practical correlate, given the earlier time point advantage.

Many of the strengths of this analysis are the same as those of our previous correlate analyses (10–12). An additional contribution of this work is the application of multivariable marker analyses, which could be conducted because the full dataset of the originally planned antibody markers became available (5). These analyses allowed comparing the strength of the antibody markers as immune correlates and assessing whether and how the antibody markers can be combined to improve an antibody-based correlate.

Limitations of this study include that it evaluated short-term efficacy only against virus strains highly similar to the vaccine-insert strain; thus, this study is a “homologous antibody correlates study.” An additional limitation of this work is that this study evaluated one specific live virus neutralization assay, which differed from that studied in (9), making it difficult to directly compare the live virus neutralization results of the two assays. It is also unknown whether alternative live virus neutralization assays would perform differently as CoPs for the mRNA-1273 vaccine. An additional fundamental limitation of any CoP analyses based on data from randomized trials is the need for strong, untestable assumptions to conclude causality. In particular, our approaches generally require the assumption of no unmeasured confounding of the marker readout and the risk of COVID-19. Although we have attempted to address this to some degree through the inclusion of causal sensitivity analyses, this fundamental assumption underlies CoP methodology. Thus, causal conclusions should be subject to additional scrutiny using alternative experimental designs. Additional limitations are the same as those of our previous correlate analyses (10–12).

Future work on the COVE study to further characterize immune CoPs of the mRNA-1273 vaccine will be to apply the binding and PsV neutralization assays to samples at 4 weeks after dose 3 and to study antibody marker measurements to Omicron strains as CoPs against COVID-19 caused by infection from the Omicron variants. These studies are planned to be conducted in SARS-CoV-2–naïve individuals with no evidence of SARS-CoV-2 infection at any time up to dose 3 and in SARS-CoV-2–nonnaïve individuals with evidence of SARS-CoV-2 infection after receiving the two-dose primary series and before dose 3. Given that the correlate analyses of COVE to date have been restricted to SARS-CoV-2–naïve individuals, COVID-19 endpoints by ancestral strain-like viruses, and antibodies to the ancestral strain, these future analyses should provide multiple insights relevant for guiding vaccine development and use in the contemporary context of the COVID-19 pandemic.

## MATERIALS AND METHODS

### Study design

The overall objective was to complete the evaluation of antibody markers measured at D29 and at D57 as CoRs and as CoPs against the primary COVID-19 endpoint in the COVE phase 3 trial of the mRNA-1273 COVID-19 vaccine. This included univariate analyses of the LV-MN_50_ marker, measured at D29 and D57, as well as multivariable analyses of the suite of measured D29 and D57 markers. The two stages of the immune correlate analysis of the COVE trial are described in the Statistical Analysis Plan in data file S1; this paper is restricted to stage 1 correlates.

Antibody markers of interest were measured using three different immune assays, detailed below: a bAb assay, a PsV-nAb assay, and a LV-MN_50_ assay. Laboratory staff conducting the immune assays were blinded to group allocation during data collection and analysis. The univariable CoR analyses of bAb and PsV-nAb markers (D29 and D57) were included in our previous work; in the present work, these markers are included in multivariable analyses. Table 7 of the Statistical Analysis Plan provides the minimum numbers of primary COVID-19 endpoint cases in the vaccine arm required for each immune correlate analysis.

Using a case-cohort sampling design (24), participants were randomly sampled for measurement of antibody markers on D1, D29, and D57; antibody markers were also measured on D1, D29, and D57 in all vaccine recipients with a breakthrough COVID-19 endpoint. The same case-cohort sets were used for the analysis of the LV-MN_50_ markers as previously used for the binding and PsV neutralization markers (10). Correlate analyses were conducted for baseline-negative per-protocol participants defined in (10) as participants with no immunologic or virologic evidence of prior COVID-19 at enrollment [as in (16)] who received both doses without major protocol violations.

The Institutional Review Board (IRB) approval number for the use of human serum samples in the PsV neutralization assay is Pro00105358 (DUHS IRB, 2424 Erwin Rd., Durham, NC, 919.668.5111, Federalwide Assurance No: FWA 00009025 Suite 405). The human specimens for Battelle’s analysis were collected from human volunteers in accordance with the requirements of Moderna Inc. IRB of record (Advarra IRB; Clinical Trial NCT04470427). All human specimens received by Battelle were coded. Biospecimens were not identifiable to Battelle, nor did Battelle have any code key or way to associate results of analysis with the original human donors. Furthermore, there was no intention to try to identity or otherwise attribute any results of analysis to the original human donors. As such, this study did not meet regulatory criteria for categorization as human subject research for the Battellespecific scope of work, and Battelle is not considered to be engaged in research according to Department of Health and Human Services–published guidance. This opinion for the use of human serum samples in the microneutralization assay is identified as IRB HSRE 389–0100142771. The opinion was provided on behalf of the Battelle IRB: Federalwide Assurance FWA00004696, IRB Registration Number IRB0000284.

### Live SARS-CoV-2 virus nAb assay

Antibody-mediated neutralization of live wild-type SARS-CoV-2 (WA isolate, passage 3, Vero-E6 cells) was measured at Battelle using a microneutralization assay (25) that has been validated for the analysis of sera collected from individuals vaccinated with mRNA-1273. This assay quantifies serum nAbs against SARS-CoV-2 using an in situ enzyme-linked immunosorbent assay (ELISA) readout.

The SARS-CoV-2 stock was produced by infecting Vero-E6 cells [African green monkey kidney, passage 31; originally obtained from BEI Resources (catalog no. NR-596)] with CDC-provided material (2019-nCoV/USA-WA1/2020; GenBank, accession number MN985325.1; passage 3) at a multiplicity of infection of 0.001 in Eagle’s minimum essential medium supplemented with antibiotics and 2% fetal bovine serum. Virus-containing supernatant was harvested after 72 hours of incubation at 37° ± 2°C and 5 ± 2% CO_2_, pooled, clarified by centrifugation, aliquoted, and stored below −70°C. Dilutions of heat-inactivated serum samples and controls were incubated with this SARS-CoV-2 stock before inoculation in singlets in a 96-well cell culture plate containing a confluent VeroE6 cell monolayer. After a 40- to 46-hour incubation, the inoculum was removed, cell plates were fixed, and an in situ ELISA was performed to detect SARS-CoV-2 antigen.

For the ELISA, plates were incubated with anti-nucleocapsid protein primary antibody cocktail (clones HM1056 and HM1057) (EastCoast Bio; catalog nos. HM1056 and HM1057) for 60 min at 37°C. The plates were washed, the secondary antibody [goat anti-mouse IgG horseradish peroxidase conjugate, Fitzgerald; catalog no. 43C-CB1569) was added to the wells, and the plates were incubated for 60 min at 37°C. Refer to U.S. patent application nos. 17/447,022 and 17/336,443 for further details. The optical density value of each sample well was measured with a microplate reader using a wavelength of 405 nm and a 490-nm reference. Each sample was tested independently in singlet by one operator on one test plate following the standard operator procedures. The same sample was then tested by a second operator in singlet on a different plate on the same day. If necessary, repeat testing of any samples was performed in singlet by one operator on a different test day. The final reportable value for each sample was the median MN_50_ titer of a minimum of two passing independent results. The WT LV-MN_50_ marker is defined as the reciprocal serum dilution at which 50% of the test SARS-CoV-2 virus is neutralized, calculated using the Spearman-Kärber method (26). The assay limits are provided in table S9; the limit of detection, equal to 22.66 IU_50_/ml, was used to define a negative versus positive neutralization response, and values below the limit of detection were assigned a value of half the detection limit. The MN_50_ readout was calibrated to the World Health Organization 20/136 anti–SARS-CoV-2 immunoglobulin International Standard (27) and converted to international units by the Fred Hutchinson Cancer Center, with units in IU_50_/ml.

### Spike-pseudotyped lentivirus nAb assay

Antibody-mediated neutralization of lentiviral particles pseudotyped with full-length SARS-CoV-2 spike protein was assessed by a validated assay (28). The nAb titer readout was calibrated to the World Health Organization 20/136 anti–SARS-CoV-2 immunoglobulin International Standard (27) and converted to international units, with units of IU_50_/ml or IU_80_/ml. Assay limits are provided in table S9; the limit of detection, 2.42 IU_50_/ml or 15.02 IU_80_/ml, was used to define a negative versus positive neutralization response. Values below the limit of detection were assigned a value of half the detection limit.

### bAb assay

Serum IgG bAbs against spike protein and against RBD were measured using a validated solid-phase electrochemiluminescence S-binding IgG immunoassay (10). Arbitrary units per milliliter were converted to bAb units per milliliter (BAU/ml) using the World Health Organization 20/136 anti–SARS-CoV-2 immunoglobulin International Standard (27) as previously described (10). Assay limits are provided in table S9; antibody response was defined by detectable IgG concentration above the antigen-specific positivity cutoff (10.8424 BAU/ml for spike protein and 14.0858 BAU/ml for RBD).

### Statistical analysis

All data analyses were prespecified in the Statistical Analysis Plan (data file S1). Use of multiple statistical methods adds robustness to the results because it limits dependence on the assumptions of a single method or model being correct. Covariate adjustment and causal interpretations were performed identically as in (10). All correlate analyses were adjusted for the following baseline variables: at-risk status [defined in (16)], community of color classification (all persons other than white non-Hispanic), and baseline risk score. We interpreted CoR analyses as associative and correlative, rather than causal analyses, although these approaches also adjust for covariates above to attempt to isolate the most meaningful association between markers and risk of COVID-19. On the other hand, our CoP analyses assume a specific causal interpretation. The assumptions required to conclude causality are strong and vary by the particular method. Generally, an important assumption is that there are no confounders of the effect of the marker on COVID-19 risk beyond the adjustment variables above. For some methods, we can explicitly evaluate the sensitivity of our findings to this assumption.

Univariate analyses of the D29 and D57 LV-MN_50_ markers were assessed as CoRs in vaccine recipients. These markers were assessed using the same statistical analysis conducted previously for the binding and PsV neutralization markers (10). Inverse probability sampling–weighted Cox regression fit using the survey R package (29) was used for point and 95% CI estimation of the covariate-adjusted hazard ratio of the COVID-19 primary endpoint across LV-MN_50_ tertiles, per 10-fold increase in quantitative LV-MN_50_ titer, or per SD increase in the quantitative LV-MN_50_ titer. Wald-based *P* values for an association of each antibody marker with COVID-19 are also reported. These Cox models were also used to estimate LV-MN_50_ marker conditional cumulative incidence of the COVID-19 primary endpoint, with bootstrap 95% CIs reported. Nonparametric dose-response regression (30) was also used to estimate LV-MN_50_ marker conditional cumulative incidence of the COVID-19 primary endpoint, with influence function–based Wald-based 95% CIs reported. Point estimates of LV-MN_50_ marker threshold conditional cumulative incidence of the COVID-19 primary endpoint and 95% point-wise CIs were calculated using nonparametric targeted minimum loss–based threshold regression (31).

A multivariable Cox model was fit (using the same fitting approach as for individual markers) that included D29 RBD IgG, D29 PsV-nAb ID_50_, and D29 LV-MN_50_. The model adjusted for the same baseline factors as those adjusted for in the univariable marker analyses. Point estimates and 95% CIs are reported for the three marker hazard ratio parameters. This analysis was also repeated using the D57 versions of the same three antibody markers. In exploratory analyses, the Cox models were fit with pairs of antibody markers, as detailed in the Statistical Analysis Plan.

Cross-validated model selection, also referred to as discrete super learning (21), was used to compare the individual-level classification accuracy of models including different combinations of input variables for predicting in vaccine recipients occurrence of the COVID-19 endpoint. In this approach, many prespecified candidate prediction models are evaluated in terms of their predictive ability, and the top model is selected using cross-validation. The learner-screener combinations that were entered into the superlearner are provided in table S10, and the variable sets that were used as input feature sets for the superlearner are provided in table S11. For each variable set, a point and 95% CI estimate of CV-AUC for the superlearner model fit is used to summarize classification accuracy. To provide an honest evaluation of the discrete Super Learner, nested cross-validation was used wherein a separate super learner was fit in each of 10 training samples, with its performance evaluated in a held-out validation sample. These Super Learner–based analyses were performed with the open source SuperLearner R package (32).

Point and 95% CI estimates of vaccine efficacy by D29 or D57 LV-MN_50_ marker values were obtained by a causal inference approach using Cox proportional hazards estimation; this statistical analysis was the same as done previously for the binding and PsV neutralization makers (16). In addition, nonparametric monotone dose-response estimation was used to obtain point and 95% CI estimates of vaccine efficacy by D29 or D57 marker values (30); these results have advantage of allowing an arbitrary nondecreasing shape of how vaccine efficacy changes with the indicated marker. Implementation of the nonparametric methods is described in the Statistical Analysis Plan.

D29 LV-MN_50_ titer was assessed as a mediator of vaccine efficacy using the method described by Benkeser *et al.* (17). D57 LV-MN_50_ titer was not assessed as a mediator of vaccine efficacy by this method, because it did not meet the prespecified criterion of having at least 10% of vaccine recipients having marker value equal to the value in placebo recipients. See the Statistical Analysis Plan for additional details.

The method by Follmann (18) was applied to compare markers in terms of the size of their standardized association with risk of COVID-19. Markers with stronger correlate signals will have higher standardized associations and therefore may be better suited for usage as an endpoint in future immunogenicity or immunobridging studies. The results of this method are presented in terms of a sample size ratio. For example, if the ratio of standardized effect size for D57 spike IgG compared with that for D57 PsV-nAb ID_50_ is 2, then a future correlates study would need to enroll twice as many participants to achieve a similar power to reject the null hypothesis using the inferior marker. In effect, the method provides a more interpretable and practicable means of comparing the magnitude of *P* values for different markers. The bootstrap method described by Follmann was used to build 95% CIs about the estimated sample size ratios.

All analysis was implemented in R version 4.0.3, and the code was verified using mock data. All *P* values are two-sided. For each set of hypothesis tests, *q* values and FWER *P* values (FWER-adjusted *P* values) were computed over the set of *P* values (separately for D29 and for D57 marker CoRs) both for quantitative markers and categorical markers (considering all five antibody markers: spike IgG, RBD IgG, PsV-nAb ID_50_, PsV-nAb ID_80_, and LV-MN_50_) using the Westfall and Young (33) permutation method (10,000 replicates).

## Supplementary Material

Suppl Material

Supp Data File S1

Suppl MDAR Reproducibility

## Figures and Tables

**Table 1.** Live virus neutralization MN_50_ titer response rates and geometric mean titers (GMTs) by COVID-19 outcome status.

		COVID-19 cases*			Non-cases in immunogenicity subcohort			Comparison	
				
Visit for marker	Marker^†^	*N* ^‡^	Response rate (95% CI)§	GMT (95% CI)	*N* ||	Response rate (95% CI)	GMT (95% CI)	Response rate difference (95% CI)	Ratio of GM (cases/non-cases) (95% CI)

D29	Live virus-MN_50_ (lU_50_/ml)	46	45.7% (31.6, 60.4%)	31.4 (22.0, 45.0)	1005	69.2% (65.8, 72.4%)	48.4 (44.6, 52.6)	−24% (−38, −8.4%)	0.65 (0.45, 0.94)

D57	Live virus−MN_50_ (IU_50_/ml)	36	100.0% (100.0, 100.0%)	594 (433, 816)	1005	99.3% (98.3, 99.7%)	718 (676, 763)	1% (0, 2%)	0.83 (0.60, 1.14)

* Cases for D29 marker correlate analyses (intercurrent cases + post-D57 cases) are baseline SARS-CoV-2–negative per-protocol vaccine recipients with the symptomatic infection COVID-19 primary endpoint diagnosed starting 7 days after D29 through the end of the blinded phase. Cases for D57 marker correlate analyses (post-D57 cases) are baseline SARS-CoV-2–negative per-protocol vaccine recipients with the symptomatic infection COVID-19 primary endpoint diagnosed starting 7 days after D57 through the end of the blinded phase. The last COVID-19 endpoint within the blinded phase occurred 100 days after D57.

† Microneutralization assay readouts were calibrated to the WHO anti–SARS-CoV-2 immunoglobulin International Standard (27) and are expressed in international units/ml (IU_50_/ml).

‡ *N* for cases for D29 marker analyses is the number of vaccine breakthrough cases with days 1 and 29 antibody marker data included, and *N* for cases for D57 marker analyses is the number of vaccine breakthrough cases with days 1, 29, and 57 antibody data included.

§ Response rate = estimated frequency of participants with MN_50_ > limit of detection (= 22.66 IU_50_/ml) as calculated using inverse probability of sampling weighting.

|| *N* for non-cases in the immunogenicity subcohort is the number of participants with days 1, 29, and 57 antibody marker data included in both the D29 and D57 marker correlate analyses, where non-cases did not experience the COVID-19 primary endpoint up to the time of the data cut and had no evidence of SARS-CoV-2 infection up to 6 days after D57 visit. The numbers of baseline-negative per-protocol participants with antibody markers measured (for each of the five immunoassays) and included in each of the D29 and D57 correlate analyses are given in table S1.

Analysis based on baselinenegative per-protocol vaccine recipients in the D29 marker or D57 marker case-cohort sets. Median (interquartile range) days from dose 1 to D29 was 28 (28 to 30) and from D29 to D57 was 28 (28 to 30).

**Table 2.** Hazard ratio for COVID-19 as nAb marker values increase.

Time point	Antibody marker*	No. cases/no. at-risk^†^	Hazard ratio	Point estimate	(95% CI)	*P* value (two-sided)	FDR-adjusted *P* value^‡^	FWER-adjusted *P* value
D29	LV-MN_50_ (IU_50_/ml)	55/14,141	Per 10-fold increase	0.39	(0.19, 0.83)	0.014	0.016	0.017
D29	PsV-nAb ID_50_ (IU_50_/ml)	55/14,141	Per 10-fold increase§	0.33	(0.17, 0.65)	0.001	0.002	0.004
D57	LV-MN_50_ (IU_50_/ml)	47/14,064	Per 10-fold increase	0.51	(0.25, 1.04)	0.065	0.075	0.108
D57	PsV-nAb ID_50_ (IU_50_/ml)	47/14,064	Per 10-fold increase§	0.42	(0.27, 0.65)	<0.001	0.003	0.002
D29	LV-MN_50_ (IU_50_/ml)	55/14,141	Per SD increase	0.62	(0.43, 0.91)	0.014	0.016	0.017
D29	PsV-nAb ID_50_ (IU_50_/ml)	55/14,141	Per SD increase	0.55	(0.38, 0.79)	0.001	0.002	0.004
D57	LV-MN_50_ (IU_50_/ml)	47/14,064	Per SD increase	0.78	(0.59, 1.02)	0.065	0.075	0.108
D57	PsV-nAb ID_50_ (IU_50_/ml)	47/14,064	Per SD increase	0.69	(0.57, 0.83)	<0.001	0.003	0.002

* Serological assay readouts assessed as immune correlates were first expressed in values relative to the WHO International Standard for anti–SARS-CoV-2 immunoglobulin (27): PsV-nAb titers and microneutralization assay readouts were calibrated to international units/ml (IU_50_/ml).

† No. at-risk = estimated number in the population for analysis: baseline-negative per-protocol vaccine recipients not experiencing the COVID-19 endpoint through 6 days after D29 visit (D29 markers) or D57 visit (D57 markers); no. cases = estimated number of this cohort with an observed COVID-19 endpoint starting 7 days after D29 visit (D29 markers) or D57 visit (D57 markers).

‡ FDR (false discovery rate)–adjusted *P* values and FWER (family-wise error rate)–adjusted *P* values were computed over the set of *P* values both for quantitative markers and categorical markers (low, medium, and high) using the Westfall and Young permutation method (10,000 replicates).

§ PsV-nAb ID_50_ hazard ratios per 10-fold increase were previously published [Fig. 3A and figure S17A of (10)] and are included here for comparison.

Analysis is based on baseline-negative per-protocol vaccine recipients in the D29 marker or D57 marker case-cohort set. Baseline covariates adjusted for baseline risk score, at-risk status, community of color status, and maximum failure event time 126 days after D29 visit (D29 markers) or 100 days after D57 visit (D57 markers).

**Table 3.** Mediation effect estimates for quantitative D29 nAb markers with 95% CIs.

Antibody marker(s)	Direct VE	Indirect VE	Proportion Mediated
D29 LV-MN_50_	84.2% (76.5, 89.3%)	53.3% (36.4, 65.7%)	29.2% (17.2, 41.2%)
D29 PsV-nAb ID_50_*	56.0% (42.2, 66.5%)	83.2% (76.9, 87.8%)	68.5% (58.5, 78.4%)
D29 PsV-nAb ID_50_ + D29 LV-MN_50_	62.0% (50.0, 71.1%)	80.6% (73.3, 85.8%)	62.9% (52.9, 72.8%)

* D29 PsV-nAb ID_50_ mediation effect point estimates were previously published [table S9 of (*10*)] and are included here for comparison.

Direct vaccine efficacy (VE) indicates VE comparing vaccine versus placebo with marker set to the value of placebo recipients (undetectable). Indirect VE indicates VE in vaccinated participants comparing observed marker versus hypothetical marker under placebo (undetectable). Proportion mediated indicates the fraction of total risk reduction from vaccine (overall 92.3% VE) attributed to the antibody marker(s) computed as 1 – log(1 – indirect VE/100)/log(1 – total VE/100).

**Table 4.** Ranking of D57 antibody marker performance in each of two categories of immune correlate quality criteria.

	Category 1 : CoR									Category 2: CoP: VE modification						
		
	HR per SD (Cox, quant.)		HR *P* value (Cox, quant.)		HR high versus low fertile (Cox)		HR *P* value fertile (Cox)		CoR: median rank	Range of CVE Pt. Est. (Cox, 5th to 95th pere.)		Range of CVE Pt. Est. (NP, 5th to 95th percentile)		*E* value marg. risk ratio 95% UCL		CoP: median rank
									
	Pt. Est. (95% Cl)	Rank	FWER	Rank	Pt. Est. (95% Cl)	Rank	FWER	Rank		Pt. Est.	Rank	Pt. Est.	Rank	*E* value	Rank	

D57 spike IgG (BAU/ml)	0.85 (0.76, 0.95)	5	0.020	4	0.23 (0.09, 0.60)	2	0.020	1	**3**	2.7	5	21.9	2	3.1	2	**2**

D57 RBD IgG (BAU/ml)	0.80 (0.70, 0.92)	4	0.010	3	0.28 (0.12, 0.67)	3	0.021	2	**3**	4.0	4	23.9	1	2.6	3	**3**

D57 PsV-nAb ID_50_ (IU_50_/ml)	0.69 (0.57, 0.83)	2	0.002	1	0.31 (0.12, 0.80)	4	0.108	4	**3**	7.5	2	17.7	4	2.0	4	**4**

D57 PsV-nAb ID_80_ (IU_80_/ml)	0.67 (0.54, 0.83)	1	0.003	2	0.20 (0.07, 0.61)	1	0.025	3	**1.5**	8.4	1	17.8	3	3.3	1	**1**

D57 LV-MN_50_ (IU_50_/ml)	0.78 (0.59, 1.02)	3	0.108	5	0.78 (0.34, 1.77)	5	0.571	5	**5**	5	3	6.0	5	1.0	5	**5**

Baseline covariates were adjusted for baseline risk score, at-risk status, and community of color status. The maximum failure event time was 100 days after the D57 visit. FWER-adjusted *P* values were computed over the set of *P* values both for quantitative markers and categorical markers (low, medium, and high) using the Westfall and Young permutation method (10,000 replicates). All serological assay readouts assessed as immune correlates were first expressed in assay values relative to the WHO International Standard for anti–SARS-CoV-2 immunoglobulin (27); bAb readouts were converted to bAb units per milliliter (BAU/ml); and PsV-nAb titers and microneutralization assay readouts were calibrated to international units/ml (IU_50_/ml or IU_80_/ml). Within both categories, D57 PsV-nAb ID_80_ had the best performance as assessed by median rank. In Category 2, Pt. Est. is computed as (1 – VE at 5th percentile)/(1 – VE at 95th percentile). CVE, controlled vaccine efficacy; NP, nonparametric; UCL, upper confidence limit.

**Table 5.** Ranking of D29 antibody marker performance in each of three categories of immune correlate–quality criteria.

	Category 1 : CoR									Category 2: CoP: VE modification							Category 3: CoP: VE mediation				
			
	HR per SD (Cox, quant.)		HR *P* value (Cox, quant.)		HR high versus low fertile (Cox)		Hazard ratio *P* value tertile (Cox)		CoR: median rank	Range of CVE Pt. Est. (Cox, 5th to 95th perc.)		Range of CVE Pt. Est. (NP, 5th to 95th percentile)		*E* value marg. risk ratio 95% UCL		CoP: median rank	Proportion mediated				CoP: median rank
											
	Pt. Est. (95% Cl)	Rank	FWER	Rank	Pt. Est. (95% Cl)	Rank	FWER	Rank		Pt. Est.	Rank	Pt. Est.	Rank	*E* value	Rank		Pt. Est. (95% Cl)	Rank	95% LCL	Rank	

D29 Spike IgG (BAU/ml)	0.73 (0.62, 0.86)	5	<0.001	1	0.19 (0.08, 0.44)	1	<0.001	1	**1**	6.6	5	16.9	3	4.5	1	**3**	–				–

D29 RBD IgG (BAU/ml)	0.68 (0.55, 0.83)	4	0.001	2	0.28 (0.13, 0.60)	3	0.005	4	**3.5**	9.3	3	14.9	4	3.0	3	**3**	–				–

D29 PsV-nAb ID_50_ (IU_50_/ml)	0.55 (0.38, 0.79)	2	0.004	3	0.32 (0.15, 0.69)	4	0.001	2	**2.5**	17.7	1	18.7	1	2.5	4	**1**	69.9 (59.8, 80.0)	1	59.8	1	**1**

D29 PsV-nAb ID_80_ (IU_80_/ml)	0.48 (0.30, 0.77)	1	0.006	4	0.22 (0.09, 0.51)	2	0.004	3	**2.5**	14.7	2	18.2	2	3.7	2	**2**	48.5 (35.0, 62.0)	2	35.0	2	**2**

D29 LV-MN_50_ (lU_50_/ml)	0.62 (0.43, 0.91)	3	0.017	5	0.46 (0.21, 1.01)	5	0.021	5	**5**	9.3	3	11.5	5	1.2	5	**5**	29.2 (17.2, 41.2)	3	17.2	3	**3**

Baseline covariates were adjusted for baseline risk score, at-risk status, and community of color status. The maximum failure event time was 126 days after the D29 visit. FWER-adjusted *P* values were computed over the set of *P* values both for quantitative markers and categorical markers (low, medium, and high) using the Westfall and Young permutation method (10,000 replicates). All serological assay readouts assessed as immune correlates were first expressed in assay values relative to the WHO International Standard for anti–SARS-CoV-2 immunoglobulin (27); bAb readouts were converted to BAU/ml, and PsV-nAb titers and microneutralization assay readouts were calibrated to IU_50_/ml or IU_80_/ml. Within category 1, D29 Spike IgG had the best performance as assessed by median rank. Within categories 2 and 3, D29 PsV-nAb ID_50_ had the best performance as assessed by median rank.

## Data Availability

All data associated with this study are present in the paper or the Supplementary Materials. Because the trial is ongoing, access to participant-level data and supporting clinical documents with qualified external researchers may be available upon request and is subject to review once the trial is complete. Such requests can be made to P.B.G. (pgilbert@fredhutch.org). The code is publicly available at Zenodo (34).
