# Supplementary material for: Comparing antibody assays as correlates of protection against COVID-19 in the COVE mRNA-1273 vaccine efficacy trial
Source: Sci Transl Med. Author manuscript; Available in PMC 2023 Jun 6. (PMC10243212; doi:10.1126/scitranslmed.ade9078)
Supplement: Suppl Material [file NIHMS1899084-supplement-Suppl_Material.pdf]

Science Translational Medicine
AAAS

Supplementary Materials for

Comparing antibody assays as correlates of protection against COVID-19 in the COVE mRNA-1273 vaccine efficacy trial

David Benkeser *et al.*

Corresponding author: Peter B. Gilbert, pgilbert@fredhutch.org

Sci. Transl. Med. **15**, eade9078 (2023)
DOI: 10.1126/scitranslmed.ade9078

The PDF file includes:

Members of the Immune Assays, Moderna Inc., CoVPN/COVE, and USG/CoVPN Biostatistics Teams
Figs. S1 to S13
Tables S1 to S11
Legend for data file S1
Reference (*35*)

Other Supplementary Material for this manuscript includes the following:

Data file S1
MDAR Reproducibility Checklist

Immune Assays Team

(PubMed listed, and ordered alphabetically by affiliation)

Affiliation	Team Members
Biomedical Advanced Research and Development Authority (BARDA), Washington, DC	Oleg Borisov, Flora Castellino, Brett Chromy, Mark Delvecchio, Ruben O. Donis, Tremel Faison, Corey Hoffman, Christopher Houchens, Tom Hu, Chuong Huynh, Pennie Hylton, Lakshmi Jayashankar, Aparna Kolhekar, James Little, Karen Martins, Jeanne Novak, Carol Sabourin, Evan Sturtevant, Xiaomi Tong, John Treanor, Danielle Turley, Leah Watson
Boston Consulting Group, Boston, MA	Gian King, Andrew Li, Najaf Shah, Smruthi Suryaprakash, Jue Xiang Wang
Division of AIDS, NIAID, NIH, Bethesda, MD	Patricia D'Souza
DMID (Microbiology and Infectious Diseases), NIAID, NIH, Bethesda, MD	Janie Russell, Aparna Kolhekar, Larry Wolfraim, Janet Lathey
Duke University, Durham, NC	David Beaumont, Rebecca Beerman, Kendall Bradley, Jiayu Chen, Xiaoju Daniell, Thomas Denny, Elizabeth Domin, Amanda Eaton, Kelsey Engel, Wenhong Feng, Juanfei Gao, Hongmei Gao, Kelli Greene, Sarah Hiles, Marianne Jessup-Cumming, Leihua Liu, Kristy Long, Kellen Lund, Kaia Lyons, Charlene McDanal, David C. Montefiori, Marcella Sarzotti-Kelsoe, Francesca Suman, Haili Tang, Jin Tong, Olivia Widman
The Tauri Group, an LMI company - Contract Support for U.S. Department of Defense (DOD) Joint Program Executive Office for Chemical, Biological, Radiological and Nuclear Defense (JPEO-CBRND) Joint Project Manager for Chemical, Biological, Radiological, and Nuclear Medical (JPM CBRN Medical), Fort Detrick, Maryland, USA	Christopher S. Badorrek, Gregory E. Rutkowski
Vaccine Research Center, NIAID, NIH, Bethesda, MD	Akua Abrah, Obrimpong Amoa-Awua, Manjula Basappa, Robin Carroll, Erykah Coe, Jevone Fentress, Britta Flach, Suprabhath Gajjala, Nazaire Jean-Baptiste, Richard A. Koup, Bob C. Lin, Adrian McDermott, Christopher Moore, Mursal Naisan, Muhammed Naqvi, Sandeep Narpala, Sarah O'Connell, Abhinaya Srikanth, Clare Whittaker, Weiwei Wu
Battelle Memorial Institute, West Jefferson, OH	Erin Altonen, Michael Anderson, Brittany Baur, Matthew Cupp, Wantong Du, Melicia Gainey, Jennifer Garver, Neil Gibson, Dalia Natour, Andrew Puttmann, Thomas Rudge, Jessica Sharits, Nancy Tirado

Moderna, Inc. Team

(PubMed listed, ordered alphabetically)

Affiliation	Team Members
Moderna, Inc., Cambridge, MA	Weiping Deng, Shu Hahn, Jacqueline Miller, Rolando Pajon, Honghong Zhou

Coronavirus Vaccine Prevention Network (CoVPN)/Coronavirus Efficacy (COVE) Team

(PubMed listed, and ordered alphabetically by institution affiliation)

Affiliation/Funding*	Study Group	Location
AB Clinical Trials	Atoya Adams, MD, MBA, Eric Miller	Las Vegas, NV
Accel Research Sites	Bruce G. Rankin DO, John Hill MD, Steven Shinn MD, Marshall Nash MD	DeLand, FL
Advanced Clinical Research	Sinikka L. Green MD, Colleen Jacobsen, Jayasree Krishnankutty, Sikhongi Phungwayo	Cedar Park, TX
Alliance for Multispecialty Research	Richard M. Glover, II MD, Drs. Stacy Slechta, Troy Holdeman, Robyn Hartvickson, Amber Grant	Newton, KS
Alliance for Multispecialty Research	Terry L. Poling MD, Terry D. Klein MD, Thomas C. Klein MD, Tracy R. Klein MD	Wichita, KS
Alliance for Multispecialty Research	William B. Smith MD, Richard L. Gibson MD, Jennifer Winbigler MD, Elizabeth Parker PA	Knoxville,TN
Baptist Health Center for Clinical Research	Priyantha N. Wijewardane, MD, Eric Bravo MD, Jeffrey Thessing MD, Michelle Maxwell APRN, Amanda Horn APRN	Little Rock, AR
Baylor College of Medicine, NIAID 1UM1AI148575-01S2	Hana El Sahly MD, Jennifer Whitaker MD, Catherine Mary Healy MD, Christine Akamine MD	Houston, TX
Benchmark Research	Laurence Chu, MD, R. Michelle Chouteau, MD	Austin, TX
Benchmark Research	Michael J. Cotugno MD, George H. Bauer, Jr. MD	Metairie, LA
Benchmark Research	Greg Hachigian MD, Masaru Oshita MD, Michael Cancilla NP, Deborah Murray NP, Kristen Kiersey NP	Sacramento, CA
Benchmark Research	William Seger MD, Mohammed Antwi, Allison Green, Anthony Kim	Fort Worth, TX
Brigham and Women's Hospital, NIAID UM1AI069412, NCATS UL1RR025758	Lindsey R Baden MD, Michael Desjardins MD, Jennifer A Johnson MD, Amy Sherman MD, Stephen R Walsh MD	Boston, MA
Carolina Institute for Clinical Research	Judith Borger DO, Ryan Starr DO, Scott Syndergaard DO, Nafisa Saleem MD	Fayetteville, NC
Centex Studies	Joel Solis MD, Martha Carmen Medina PA-C, Westly Keating PA-C, Edgar Garcia PA-C, Cynthia Bueno PA-C	McAllen, TX
Clinical Research Atlanta	Nathan Segall MD, Nathan Segall, Jon Finley, Mildred Stull	Stockbridge, GA
Clinical Trials of Texas	Douglas Scott Denham DO, Thomas Weiss MD, Ayoade Avworo DNP, Parke Hedges MD	San Antonio, TX
Coastal Carolina Research Center	Cynthia Becher Strout MD, Rica Santiago, Yvonne Davis, Patty Howenstine, Alison Bondell	Mount Pleasant, SC
Cornell Clinical Trials Unit - Weill Cornell Uptown & Weill Cornell Chelsea, NIAID UM1AI068619, NCAT UL1TR002384	Kristin Marks MS MD, Grant Ellsworth, MS, MD, Tina Wang, MD, Timothy Wilkin, MD, MPH, Mary Vogler, MD, Carrie Johnston, MD, MS	New York, NY
Covid19 Prevention Network (CoVPN, NIAID-NIH)	Michele P Andrasik, Jessica G Andriesen, Gail Broder, Lawrence Corey, Niles Eaton, Kathleen M Neuzil, Huub G Gelderblom, James G Kublin, Rachael McClennen, Nelson Michael, Merlin Robb, Carrie Sopher	Seattle, WA
DM Clinical Research	Vicki E. Miller MD, MPH, Fredric Santiago MD, Blanca Gomez FNP-C, Insiya Valika PA-C, Amy Starr FNP-C	Tomball, TX
Emory University – Ponce de Leon Clinical Research Site, NIAID 3UM1AI068614-14S1	Colleen Kelley MD MPH, Valeria D Cantos MD, Sheetal Kandiah MD MPH, Carlos del Rio MD	Atlanta, GA
Emory University – Hope Clinic, NIAID 1UM1AI148576-01	Nadine Rouphael MD, Paulina Rebolledo, Srilatha Edupuganti, Daniel Sans Graciaa	Decatur, GA
Emory University School of Medicine, NIAID 1UM1AI148576-01	Evan J Anderson MD, Andres Camacho-Gonzalez MD, Satoshi Kamidani MD, Christiana A Rostad MD, Meghan Teherani MD	Atlanta, GA
George Washington University, NIAID UM1AI068619	David Joseph Diemert MD, Elissa Malkin, Marc Siegel, Afsoon Roberts, Gary Simon	Washington, DC
Hackensack University Medical Center	Bindu Balani MD, Carolene Stephenson, Steven Sperber, Cristina Cicogna	Hackensack, NJ
Henry Ford Health System	Marcus J. Zervos MD, Paul Kilgore MD, MPH, Mayur Ramesh MD, Erica Herc MD, Kate Zenlea MPH	Detroit, MI
Hope Research Institute	Abram Burgher MD, Ann Marie Milliken	Phoenix, AZ
Hope Research Institute	Joseph D. Davis MD, Brendan Levy, Sandra Kelman	Chandler, AZ
Hope Research Institute	Matthew W. Doust MD, Denise Sample, Sandra Erickson	Phoenix, AZ
J. Lewis Research	Shane Glade Christensen MD, Christopher Matich, James Longe, John Witbeck	Salt Lake City, UT
J. Lewis Research	James Todd Peterson MD, Alexander Clark, Gerald Kelty, Issac Pena-Renteria	Salt Lake City, UT

Affiliation/Funding*	Study Group	Location
Jacksonville Center for Clinical Research	Michael J. Koren MD, Darlene Bartilucci MD, Jeffery Jacqmein MD, Alpa Patel MD, Carolyn Tran MD	Jacksonville, FL
Javara	Christina Kennelly MD, Robert Brownlee, Jacob Coleman, Hala Webster	Charlotte, NC
Johnson County Clin-Trials	Carlos A. Fierro MD, Natalia Leistner, Amy Thompson, Celia Gonzalez	Lenexa, KS
Kaiser Permanente Washington Health Research Institute, NIAID 1UM1AI148373-01	Lisa A Jackson MD MPH, Janice Suyehira MD	Seattle, WA
Laguna Clinical Research Associates	Milton Haber MD, Maria M. Regalado MD, Veronica Procasky RN JD, Alisha Lutat	Laredo, TX
Lynn Health Science Institute	Carl P. Griffin MD, Raymond Cornelison, William Schnitz, Shanda Gower	Oklahoma City, OK
Lynn Institute of the Rockies	Ripley R. Hollister MD, Jeremy Brown DO, Melody Ronk PA-C	Colorado Springs, CO
M3 Wake Research	Wayne Lee Harper MD, Lisa Cohen DO, Lynn Eckert PA-C, Matthew Hong MD	Raleigh, NC
MediSync Clinical Research Hattiesburg Clinic	Rambod Rouhbakhsh MD, MBA, Elizabeth Danford MD, John Johnson MD, Richard Calderone MD	Petal, MS
Meridian Clinical Research	Shishir Kumar Khetan MD, Oyebisi Olanrewaju AC-CRNP, Nan Zhai NP-C, Kimberly Nieves AC-CRNP, Allison O'Brien AC-CRNP	Rockville, MD
Meridian Clinical Research	Paul Simon Bradley MD, Amanda Lilienthal MSN NP-C, Jim Callis PA-C	Savannah, GA
Meridian Clinical Research	Adam Benson Brosz MD, Andrea Clement PA, Whitney West APRN, Luke Friesen PA, Paul Cramer APRN	Grand Island, NE
Meridian Clinical Research	Frank Steven Eder MD, Ryan Little FNP, Victoria Engler FNP, John Tarbox FNP, Heather Rattenbury-Shaw DO	Binghamton, NY
Meridian Clinical Research	David Jon Ensz MD, Tavane Harrison, Allie Oplinger	Dakota Dunes, SD
Meridian Clinical Research	Brandon James Essink MD, Jay Meyer MD, Frederick Raiser, III MD, Kimberly Mueller APRN, Roni Gray PA	Omaha, NE
Meridian Clinical Research	Keith William Vrbicky MD, Charles Harper MD, Chelsie Nutsch MD, Wendell Lewis III MD, Cathy Laflan MD	Norfolk, NE
Meridian Clinical Research	Jordan L. Whatley MD, Nicole Harrell MD, Amie Shannon MD, Crystal Rowell APRN, FNP-C, Christopher Dedon APRN, FNP-C	Baton Rouge, LA
NIH	Mamodikoe Makhene MD MPH	Bethesda, MD
New Horizons Clinical Research	Gregory Mark Gottschlich MD, Kate Harden PA-C, Melissa Gottschlich PA-C, Mary Smith MSN, FNP-C, Richard Powell MD	Cincinnati, OH
Optimal Research	Murray A. Kimmel DO, Simmy Pinto MD	Melbourne, FL
Optimal Research	Timothy P. Vachris MD, Mark Hutchens MD, Stephen Daniels DO, Margaret Wells MD	Austin, TX
Optimal Research	Mimi Van Der Leden MD, PhD, Peta Gay Jackson Booth MD	Rockville, MD
Palm Beach Research Center	Mira Baron MD, Pamela Kane DO, Shannen Seversen PA-C, Mara Kryvicky PA-C, Julia Lord PA-C	West Palm Beach, FL
Paradigm Clinical Research Center	Jamshid Saleh MD, Matthew Miles, Rafael Lupercio	Redding, CA
Quality of Life Medical & Research Centers	John W. McGettigan Jr. MD, Walter Patton MD, Riemke Brakema MD, Karin Choquette MSN, ABNP-C, Jonlyn McGettigan MSN, RN	Tucson, AZ
Rancho Paseo Medical Group	Judith L. Kirstein MD, Marcia Bernard NP	Banning, CA
Rapid Medical Research	Mary Beth Manning MD, Joan Rothenberg MD, Toby Briskin MD, Denise Roadman PAC, Sharita Tedder-Edwards FNP	Cleveland, OH
Research Centers of America	Howard I. Schwartz MD, Surisday Mederos, Barbara Corral, Jennifer Schwartz, Nelia Sanchez-Crespo	Hollywood, FL
Rutgers New Jersey Medical School, NIAID UM1AI068619	Shobha Swaminathan MD, Amesika Nyaku MD MS, Tilly Varughese MD, Michelle DallaPiazza MD	Newark, NJ
Saint Louis University, NIAID 1UM1AI148685-01	Sharon E Frey MD, Irene Graham MD, Getahun Abate MD PhD MSc, Daniel Hoft MD PhD	St. Louis, MO
St. Vincent's Health System	Leland N. Allen III MD, Leslie Anne Edwards MSN, CRNP, William Simpson Davis Jr., MS PA-C, Jessica Maria Mena, PA	Birmingham, AL
Suncoast Research Group	Mark E. Kutner MD, Jorge Caso MD, CPI, Maria Hernandez Moran APRN, Marianela Carvajal APRN, Janet Mendez APRN	Miami, FL
Sundance Clinical Research	Larkin T. Wadsworth III MD, Horacio Marafioti, Lyly Dang, Jennifer Berry, Lauren Clement	St. Louis, MO
Synexus Clinical Research	Michael Ryan Adams MD, Leslie Iverson PA	Murray, UT
Synexus Clinical Research	Joseph Lee Newberg MD, Laura Pearlman MS, MD, MBA	Chicago, IL
Synexus Clinical Research	Paul Joseph Nugent DO, Leonard Singer	Cincinnati, OH

Affiliation/Funding*	Study Group	Location
Synexus Clinical Research	Michele Diane Reynolds MD, Jennifer Bashour MD, Robert Schmidt MD	Dallas, TX
Synexus Clinical Research	Neil Parmanand Sheth MD, Kenneth Steil DO	Glendale, AZ
Synexus Clinical Research	Ramy Joseph Toma MD, William Kirby MD, Pink Folmar MD, Samantha Williams NP	Birmingham, AL
Synexus Clinical Research	Judith White MD, Robert Meyer MD, Sejal Patel MD, Prity Patel APRN	Orlando, FL
Tekton Research	Paul Pickrell MD, Stefanie Mott FNP-C, Carol Ann Linebarger MD, Hussain Malbari MD, David Pampe MD	Austin, TX
Texas Center for Drug Development	Veronica G. Fragoso MD, Lisa Holloway MD, Cecilia McKeown-Bragas MD, Teresa Becker MD, Vicki Miller MD	Houston, TX
Trial Management Associates	Barton G. Williams MD, William H. Jones MD	Wilmington, NC
VA Greater Los Angeles Healthcare System	Michael Lewis MD, Elham Ghadishah, Joseph Yusin, Mai Pham	Los Angeles, CA
University of California Los Angeles, NIAID UM1AI068619	Jesse L Clark MD, Steven Shoptaw PhD, Michele Vertucci PA, NP, Will Hernandez NP	Los Angeles, CA
University of California San Diego, NIAID UM1AI068636	Stephen A. Spector MD, Amaran Moodley MD, Jill Blumenthal MD, Lisa Stangl NP, Karen Deutsch NP	La Jolla, CA
University of Chicago	Kathleen M. Mullane DO PharmD, David Pitrak MD, Cheryl Nuss FNP, Judy Pi PharmD	Chicago, IL
University of Cincinnati, NIAID UN1AI068619	Carl Fichtenbaum MD, Margaret Powers-Fletcher PhD, Michelle Saemann RN, Sharon Kohrs RN	Cincinnati, OH
University of Colorado Denver, Anschutz Medical Campus, NIAID UM1AI068636	Thomas B. Campbell MD, Andrew Lauria, Jose Castillo Mancilla, Hillary Dunlevy	Aurora, CO
University of Illinois at Chicago – Project WISH, NIAID UM1AI068619	Richard M Novak MD, Andrea Wendrow, Scott Borgetti, Ben Ladner	Chicago, IL
University of Maryland School of Medicine, NIAID 1UM1AI148689-01	Karen L Kotloff MD, Matthew Laurens, Milagritos Tapia, Lisa Chrisley, Cheryl Young	Baltimore, MD
University of Miami, NIAID 3UM1AI068614-14S1	Susanne Doblecki-Lewis MD, Maria Luisa Alcaide, Jose Gonzales-Zamora, Stephen Morris	Miami, FL
University of North Carolina at Chapel Hill, NIAID UM1AI068619	Cynthia Gay MD MPH, David Wohl MD, Joseph Eron, Jr. MD	Chapel Hill, NC
University of Pennsylvania, NIAID 3UM1AI068614-14S1	Ian Frank MD, Debora Dunbar, David Metzger, Florence Momplaisir	Philadelphia, PA
University of Pittsburgh Medical Center, NIAID 1UM1AI148452-01	Judith Martin MD, Alejandro Hoberman MD, Timothy Shope MD MPH, Gysella Muniz MD	Pittsburgh, PA
University of Texas Medical Branch, NIAID 1UM1AI148575-01	Richard Rupp MD, Amber Stanford PA-C, Megan Berman MD, Laura Porterfield MD	Galveston, TX
VA Greater Los Angeles Healthcare System	Michael Lewis MD, Elham Ghadishah, Joseph Yusin, Mai Pham	Los Angeles, CA
Vanderbilt University Medical Center, NIAID 1UM1AI148452-01	Clarence Buddy Creech II MD, Shannon Walker MD, Stephanie Rolsma MD PhD, Robert Samuels, Isaac Thomsen MD	Nashville, TN
Vanderbilt University Medical Center, NIAID 3UM1AI068614-14S1	Spyros Andrews Kalams MD, Greg Wilson MD	Nashville, TN
Velocity Clinical Research	Gregg H. Lucksinger MD, Kevin Parks MD, Ryan Israelsen MD, Jaleh Ostovar FNP-C, Kary Kelly FNP-C	Medford, OR
Velocity Clinical Research, San Diego	Jeffrey Scott Overcash MD, Hanh Chu, Kia Lee, Karla Zepeda	La Mesa, CA
VitaLink Research	Luis I. De La Cruz MD, Steve Clemons, Elizabeth Everette, Suzanna Studdard	Greenville, SC
VitaLink Research	Gowdhami Mohan MD, Stefanie Tyson, Alyssa-Kay Peay, Danyel Johnson	Anderson, SC
VitaLink Research-Spartanburg	Gregory J. Feldman MD, May-Yin Suen, Jacqueline Muenzner, Joseph Boscia, Farhan Siddiqui	Spartanburg, SC
Wake Forest University Health Sciences	John Sanders MD,PhD, James Peacock MD, Julio Nasim MD	Winston Salem, NC
WR-Clinical Research Center of Nevada	Michael L. Levin MD, Julie Hussey MSN APRN FNP-C, Marcy Kulic MD	Las Vegas, NV
WR-ClinSearch	Mark Montgomery McKenzie MD, Teresa Deese, Erica Osmundsen, Christy Sweet	Chattanooga, TN
WR-Global Medical Research	Valentine Mbepson Ebuh MD MA MSc, Elwaleed Elnagar MD, Georgette Ebuh DNP APRN FNP-C, Genevieve Iwuala FNP	Dallas, TX
WR-Medical Center for Clinical Research	Laurie J. Han-Conrad MD, Todd Simmons MD, Denis Tarakjian MD	San Diego, CA

*Funding of institutions by the National Institute of Allergy and Infectious Diseases (NIAID) or research support by the National Center for Advancing Translational Science (NCATS) as indicated. All other institutions were funded by Office of the Assistant Secretary for Preparedness and Response, Biomedical Advanced Research and Development Authority. The content of this publication is solely the responsibility of the authors and does not necessarily represent theofficial views of the funding sources.

CoVPN/COVE Team (cont'd): COVE Trial Investigators and Study Teams

Principal Investigator	Study Team	Institution		Location
Atoya Adams, MD, MBA	Miriah Campbell, Eric Miller, Daisy Langarica, Alia Bober, Diana Giraldo	AB Clinical Trials		Las Vegas, NV
Michael Ryan Adams, MD	Leslie Iverson, Andryelle Toledo, Melinda Bullington, Alicia Hanten, Carolyn Taylor, Shannon Wright, Chase Carnahan, Rachel Law, Natalie Smith, Julie Taylor, Jared-Robert Blake, Stefanie Vasconez, Courtney Jensen	Synexus Clinical Research		Murray, UT
Leland N. Allen III, MD	Leslie Anne Edwards, William Simpson Davis, Jr., Ronald Meza, Jordan Stauffer, John Farringer, Faith Holmes, Rhonda Buzbee, Cristina Velez, Huse Lisa, Lisa Huse,Camelia Speegle, Gregory Prestage, Mary Perez, Jessica Space, Matthew Todd, Jessica McDowell, Marha Bunnell-Pollak, Jackie Ziegler, Jasmine Ali, Dumitru Sirbu, Kellie Williams, Logan Sawyer, Richelle Chambliss, Samantha Blackmon, Stephanie Brennan, Tiffany Gibbs, Alexandria Anderson, Caitlin Roll, Candace Robinson, Zachary McCoy, Jessica Bartlett, Kimberly Cornelison, Chris Bovell, Vincent Baglini, Christy Greenhalgh, Jessica Maria Mena, David House, Matt Honold, Esteban Zurita	St. Vincent's HealthSystem		Birmingham, AL
Evan J. Anderson, MD	Kathleen Stephens, Francine Dyer, Maya Stagg, Aaliyah Carron, Austin Lu, Julia Barton, Sy Tran, Leisa Bower, Esther Park, Jianguo Xu, Rebecca Gonzalez, Vy Ngo, Mike Shepard, Lezly Roxxette Zepeda, Karen Sytsma, Sandra Rojas-Honan, Felicia Glover, Susan Rogers, Theda Gibson, Christina A. Rostad, Andres Camacho- Gonzalez, Teresa Ball, Satoshi Kamidani, Mehgan Farah Teherani, Vikash Patel, EtzaPeters, Peggy Kettle, Lisa Macoy, Cindy Lubbers, Amber Samuel, Laila Hussaini, Kathryn Zaks, Caroline Ciric, Meg Taylor, Oliver Smith, Amy Muchinsky, SydneyBiccum, Laura Clegg, Dean Kleinhenz, Angelle Ijeoma, Hannah Huston	Emory University School of Medicine		Atlanta, GA
Lindsey Baden, MD	Xhoi Mitre, Jon Gothing, Bruce Bausk, Jessica Cauley, Natalie Izaguirre, Lewis Novack, Michael Seaman, Katherine Yanosick, Henry Rutherford, Junghyun Kim, Dominique Betterbed, Kathleen Garvey, Lauren Clore, Alexander Mills, Deepesh Duwadi, Alessandra Setaro, Kyl Bowman, Kevin McManus, Sidali Beriane, Fadi Ghantous, Christy Lavine, Jasper Ophel, Joseph Sapiente, Jessica Dorning, Tessa Speidel, Lauren Garneau, Robert Dannemiller, Kirquenique Rolle, Mulika Chhorn, Bailey McCarthy, Hana Flaxman, Milenko Tanasijevic, Cameron Nutt, Javier Barria, Andre Avila-Paz, Buteau Malhaika, Tong Alexandra, Tenaizus Woods, Bethany Evans, Hannah Jin, LaKeisha Gandy, Stephanie St. Pierre, Carolyn Darcy, Michael Corrado, James Maguire, Adetoun Okenla, Tamara Roldon Sevilla, David Kubiak, Cassandre Titus, Movita Harrigan, Maria Alvarado, Rose Theodat, Amy Sherman, Laura Platt, Kirsten Goodman, Laura Nicholson, Wilfredo Matias, Emily Koleske, RuthRodriguez, Nicole Taikeff, Jun Bai Park Chang, Julia Klopfer, Phoebe Cunningham, Elizabeth Sampson, Karen Magsipoc, Maureen Macgowan, Lauren Donahue, Haley Schram, Noah Abasciano, Megan Powell, Janet Morgan, Yazed Alsowaida, Olivia Riccardi, Neha Limaye, Virginia Loudermilk, Austin Kim, Kevin Zinchuk, Caitlin Grant,Charles Kelly, David Mellace, Jamie Myers, Erika Gribb, Jose Licona, Monica Feeley,Stephen R Walsh, Jennifer A Johnson, Ann Woolley, Alexis Liakos, Jane Kleinjan, Jon Gothing, Nicolas Issa, Michael Desjardins, Raphael Dolin, Alka Patel, OpeyemiTalabi, Christin Price, Paulette Chandler, Elizabeth W Karlson, Allison P Moriarty	Brigham and Women's Hospital		Boston, MA
Bindu Balani, MD	Smith Kerowyn, Sergio Garcia, Charo Valdez, Shelly Chin, Caitlin DiBello, Silvia Lara, Chika Ekweghariri, Abena Roberts, Abimbola Coker, Marie-Therese Estanbouli, Greg Eskinazi, Michael Tortoriello, Jay Elkareh, Meral Karakoc, Olga Spathis, Patrice Hassoun, Carolene Stephenson, Steven Sperber, Kaur Harveen, Cristina Cicogna, Ciaran Mannion	Hackensack University MedicalCenter		Hackensack, NJ
Mira Baron, MD	Pamela Kane, Maria Bermudez, Shannen Seversen, Mara Kryvicky, Julia Lord, Terri Barr, Daisy Acevedo, Elena Acosta, Delta Anderson, Alexandra Arango, Anne Bauer, Joshua Egbehor, Tim Flanary, Audrey Haber, Carol Henao, Patti Isaacson, Peter Jacob, Sakaiya Jackson, Karen Kodes, Ludovic La-Branche, Kimarie Lee- Russell, Carol Liso, Cristina Liso, Stephanie Morse, Michelle Navarrette, Christy Norcross, Nora Norcross, Annette Pitts, Mary Sergalis, David Scott, Tytiana Spearman, Danielle Theodore, Brian Thomas, Jennifer Torres	Palm Beach Research Center		West Palm Beach, FL
Judith Borger, DO	Jennifer Angell, Nicole Austin, Deanna Benz, Lucian Cappoli, Nicole Davis, Lynn Eckert, Kathryn Hostetter, Stephanie Keating, Jeanette Mangual-Coughlin, Avia McClain-Stocker, Ifeanyi Momodu, Cheryl Norris, Brennan Opanasenko, Stacey Saldua, Nafisa Saleem, Amy Sheets, Ryan Starr, Scott Syndergaard, Jennifer Thomas, Michelle Wallace, Jeffery Pemberton, Mitchell Arildsen, Dan Tomita	Carolina Institute forClinical Research		Fayetteville, NC
Paul Simon Bradley, MD	Taja Adams, Stephanie Ailey, Kira Bell, Shanice Bennett, Vincent Bernades, Jim Callis, Bounphone Chanthavong. Taryn Collett, Anne Crouch, Shannon Davis, Morgan Deal, Mimi Duncan, Brandon Essink, Laura Falcone, Debra Gabrielson, Brooke Halpern, Anyfa Hanna, Cassie Heisey, Dawn Kalloniatis, Andrew Kimball, Jeanette Lee, Amanda Lilienthal, Ginny McNew, Crystal Neely, Kay Lynn Olmsted, Nicole Osborn, Chevon Roberts, Pechoka Sanders, Cynthia Seedorf, Kathryn Stoddard, Jonathan Whelan, Stella Yoon	Meridian Clinical Research		Savannah, GA

Principal Investigator	Study Team	Institution	Location
Adam Benson Brosz, MD	Rhonda Richter, Debra Gabrielson, Kayla Flege, Ashley Bell, Karen Jo Johnson, Paul Cramer, Jessica Stanton, Andrea Clement, Whitney West, Laura Falcone, Amanda Friesz, Kathy Osborne, Summer Tophoj, Kimber Breeden, Susan Newman, Douglas Herbek, Lindsey Mettenbrink, Luke Friesen, Alison Pierce	Meridian Clinical Research	Grand Island, NE
Abram Burgher, MD	Stephanie Catanzaro, Shauna Harrell, Magen Hess, Nate Alderson, Bettie D'Nise Corcoran, Norma Frederick, Adrian Alejo, Brian DeCraene, Karen Wakefield, Scarlett Hammett, Susan DeCraene, Ann Marie Milliken, Neil Pearson, Donald Terral Harper	Hope Research Institute	Phoenix, AZ
Thomas B. Campbell, MD	Andrew Lauria, Jenelynn Kimble, Steven Johnson, Matin Krsak, Andrew Monte, Patrisha Adkins, Michelle Barron, Suzanne Fiorillo, Amy Harrison, Anderson Victoria, Nga Le, Sara Berech, Jose Castillo-Mancilla, Kristine Erlandson, Laurel Ware, Josie Marshall, Stephen Bartlett, Hillary Dunlevy	University of Colorado Denver, Anschutz Medical Campus	Aurora, CO
Shane Glade Christensen, MD	Christopher Mickelson, Jessica Shaw, Emily Raming, Amy Nelson, Gabrielle Lewis, Jenessa Folsom, Mikaela Jones, Dylan Owen, Rachel Pugmire, Jennifer Bradley, Annjanette Kemp, Krista Marti, Allyson Christensen, Madison Ellis, Holly Anderson, Emily Bloomquist, Ross Brunetti, Thomas Conner. Jr., Gina Cox, Diana Grazulis, Wesley Lewis, James Longe, Christopher Matich, Bryan Nelson, Sarah Scott, John Witbeck, Stephen Wood	J. Lewis Research	Salt Lake City, UT
Laurence Chu, MD	Jennifer Bacchi, Maria Barrientes, Lamar Box, Christian Casas, R. Michelle Chouteau, Katherine Davis, Tambra Dora, Cindy Duran, Pamela Fidler, Ruth Fitch, Brooke Harris, Isaiah Knight, Jennifer Leyva, Michelle Listz, Jennifer Montes, Javier Perez, Jessica Ruff, Dean Skiles, Sean Turnbow, Francesca Vigil, Breana Wade, Kelly Weber	Benchmark Research	Austin, TX
Jesse L. Clark, MD	Sandy MacNicoll, Somaieh Talebi, Timothy Hall, Steven Shoptaw, Emery Chang, Michael Li, David Goodman, Paul Adamson, Oladunni Adeyiga, Inez Bentancourt, Susan Reed, Christopher Blades, Jasmin Tavares, Demetria Villanueva, Simone Riley, Jonathan Veloz, Schuyler Thomas, Will Hernandez, Jennifer Baughman, Mitchell Stern, Michele Vertucci	University of California, Los Angeles	Los Angeles, CA
Michael J. Cotugno, MD	Kyra Lawson, Kim Harper, Edwin Adamson, George H. Bauer Jr., Julie Bilich, Brenda Lawson, Brandon Illickal, Lois Eaglin, Heather Salisbury, Jeff Segner	Benchmark Research	Metairie, LA
Clarence Buddy Creech II, MD	Shanda Phillips, Naomi Kown, Katherine Sokolow, Wendy Winn, Katherine Wright, Shannon Walker, Stephanie Rolsma, Anna Gallion, April Hanlotxomphou, Deborah Myers, Robert Adkisson, Natalia Jimenez, Cindy Trimmer, Roberta Winfrey, Matthew Donio, John Oleis, Donna Torr, Shelly McGehee, Robert Samuels, Sandra Yoder, Eric Brady, Isaac Thomsen, Madeleine Guy, Emma Alexander, Lana Howard, Krisha Alexander, Shane Moore, Tacora Wright, Tara Evans, Ursula Powell, Jenna Caserta, Valerie Mitchell, Meryk Moore, Melissa Lehman, Diane Anders, Constance Dotye, Crystal Rice, Lamar Bowman, Sherri Hails, Monique Bennett, Nicki Soper, Leigh Howard	Vanderbilt University Medical Center	Nashville, TN
Joseph D. Davis, MD	Sandra Kelman, Sandra Braden, Sabrina Bolland, Mia Munoz, Jose Barocio, Brendan Levy, Dhwani Shah, Neil Pearson, Stephanie Catanzaro, Nathan Alderson, Susan DeCraene, Maureen Godfrey, Skyla Clark	Hope Research Institute	Chandler, AZ
Luis I. De La Cruz, MD	Amy Ford, Taylor Wilson, Cindy Smith, Austin Lambert, Erin Zeiler, Kaelyn Rowland, Marlee Smith, Suzanna Studdard, Zandra Hamilton, Meredith Benfield, Sara Poff, David Godwin, Elizabeth Everette, Steven Clemons, Kayla Peay, Stephanie Gilreath	VitaLink Research	Greenville, SC
Douglas Scott Denham, DO	Thomas Weiss, Parke Hedges, Ayoade Avworo, Kay Scroggins, Leisel Koerber, Antonio Gutierrez, Nathan Cortez, Andrea Gomez, Darlington Akahara, Michelle Smith, Kristy Trevino, Beatriz Herrera, Shaiane Dickerson, Kerry de Jesus, Matthew Korte, Cynthia Ramos, Reanna Martinez, Erica Leal, Shakera Flores, Paul Esparza, Brian Hemming, Melinda Axton, D'Andre White, Terri Perez, Carolina Coronado, Rebecca Many, Clayton Stone, Kimberly Evans, Anshumaan Maharaj, Stephen Brick, Steffanie Barrera, Staci Poettgen, Dawn Killian, Gerardo Pena, Karol Perez, Victoria Hernandez, Kevin Martinez, Amy Griffith, Nolan Payton, Quincey Hogue, Jamie Padilla, Emily Mendez, Lily Hays, Maristelle Co, Nicholas Trinidad, Ismael Rodriguez, Amy Lewis, Cindi Nellis, Lele Simmons, Marissa Johnson	Clinical Trials of Texas	San Antonio, TX
David Joseph Diemert, MD	Linda Witkin, Aimee Desrosiers, DeEnna Wedding, Bertran Walton, LaKeisha Queen, Ryan Mouton, Caroline Thoreson, Manya Magnus, Jennifer Wald, Erika Faust, Nicholas Heredia, Robbie Kattappuram, Hira Qadir, Chelsea Ware, Hannah Yellin, Kegan Dasher, Daniel Mullen, Jeanne Jordan, Taylor Ladson, Madison Lintner, Kaitlyn Macnair, Bitana Saintilma, Kelly Thomas, Samantha Walker, Neha Rampally, Madhu Balachandran, Elissa Malkin, David Parenti, Hana Akselrod, Marc Siegel, Gary Simon, Afsoon Roberts, Aileen Chang	George Washington University	Washington, DC

Principal Investigator	Study Team	Institution	Location
Susanne Doblecki-Lewis, MD	Maria Luisa Alcaide, Jose Gonzalez-Zamora, Stephen Morris, Yimy Puerto, Annie Salvarrey, Claudia Balgas, Claudia Santos, Katherine King, Brahian Steven Erazo, Mayra Fernandez, Leopoldo Cordova-Garcia, Elisa Corzo-Sanchez, Edgar Fernandez, Loreta Padron, Stefani Ann Butts, Kenia Moreno, Juan Casuso, Maria de Pilar Valanzasca, Thomas Tanner, Marilyn Fernandez, Mary Aloise, Inza Patton, Vivian Pastrana, Sendy Puerto, Irma Barreto Ojeda, Junlin Long, Barbara Huang, Gilianne Narcisse, Vanessa Perez	University of Miami	Miami, FL
Matthew W. Doust, MD	Denise Sample, Sandra Erickson, Nate Alderson, Adrian Alejo, Stephanie Catanzaro, Susan DeCraene, Cassie Enricco, Sandra Erickson, Alex Guereque, Shauna Harrell, Shana Harshell, Stephanie Junker, Stephanie Laufenberg, Madison Mikulak, Makayla Morra, Nicole Olson, Neil Pearson, Jasmin Redden, Monique Romo, Denise Sample, Dhwani Shah, Sahara Vega, Emma Kar	Hope Research Institute	Phoenix, AZ
Valentine Mbepson Ebuh, MD	Elwaleed Elnagar, Georgette Ebuh, Genevieve Iwuala, Catina Adams, Marissa Cervenka, Ezgar Del Real, Shraddha Dubal, Elwaleed Elnagar, Jenifer Fiatte, Kathy Harrell, Genevieve Iwuala, Vicki Martinez, Robert Miranda, Brennan Opanasenko, Destiny Robinson, Liz Ruiz, Amy Sheets, Shoniece Wallace	WR-Global Medical Research	Dallas, TX
Frank Steven Eder, MD	Ryan Little, Victoria Engler, John Tarbox, Heather Rattenbury-Shaw, Deborah Hubish, Jessie Taylor, Debra Gabrielson, Jessica Fellows, Jennifer Molstead, Kathe Olmstead, Ashley Conover, Tammy Kohn, Chelsea Briar, Corrine Young, Collen McVannan, Kelli Quick, Shaylynne Hubanks, Kimber Breeden, Ann Marie Sampson, Traci Hull, Tarin Gordon, Susan Owen, Kate Macarak, Tonya Rackett, Jacob Blattstein, Partidge Jane Aton, Nicole Croft, Carolyn Grausgruber, Rebecca Miller, Ryan Little, Victoria Engler, John Tarbox, Heather Rattenbury-Shaw, Nathan Kimball, Courtney Heisey, Ginny McNew, Abigail Wine, Cindi VanKuren, Jared Frick, Tammy Dennis, Andrew Kimball	Meridian Clinical Research	Binghamton, NY
Hana M. El Sahly, MD	Jennifer A. Whitaker, C. Mary Healy, Christine Akamine, Wendy A Keitel, Robert L Atmar, Annette Nagel, Sandra Francisco, Thea Marie Cordero, Janet Brown, Jennifer Christensen, Caroline Doughty-Skierski, Connie Rangel, Carrie Kibler, Coni Cheesman, Lisreina Toro, Chanei Henry, Chianti Wade Bowers, Pedro Piedra, Kathy Bosworth, Kayla Burrell, Jesus Banay, Tykel Eddy, Trent Davis, Shetel Anassi, Yvette Rugeley, Olga Rybina-Willis	Baylor College of Medicine	Houston, TX
David Jon Ensz, MD	Pamela Allen, Taylor Bergh, Kimber Breeden, Avery Dunn, Brandon Essink, Debra Gabrielson, Rylea Gulick, Tavane Harrison, Courtney Heisey, Andrew Kimball, Shelby Klaschen, Jessica Knight, Makayla Langston, Meagan Miller, Allie Oplinger, Heather Persinger, Alison Pierce, Kathryn Stoddard, Kayla Sturgeon, Jamie Thompson, Melissa Wiseman	Meridian Clinical Research	Dakota Dunes, SD
Brandon James Essink, MD	Jay Meyer, Frederick Raiser, Kimberly Mueller, Roni Gray, Riley Brockman, Tabitha Campbell, Carrie Essink, Laura Falcone, Roni Gray, Linda Layton, Jay Meyer, Kimberly Mueller, Tiffany Nemecek, Frederick "Fritz" Raiser, III, Jessica Satorie, Chelsea Steinmetz, Nicole Osborn, Cassie Heisey, Maria Nguyen	Meridian Clinical Research	Omaha, NE
Gregory J. Feldman, MD	May-Yin Suen, Brittany Cooksey, Madison Fowler, Sarah Chynoweth, Gary Clemons, Laura Jolly, Charlie Jordan, Heather Allison, Steve Clemons, Amber Brittany Belcher, Allison Kelly, Marsha Gossett, Wendy Taylor, Amy Witt, Kendal Nelson, Jeffrey Witt, Jacqueline Muenzner, Elizabeth Everette, Supinder Channa, Allison Ayers, Joseph Boscia, Farhan Siddiqui	VitaLink Research-Spartanburg	Spartanburg, SC
Carl J. Fichtenbaum, MD	Maggie Powers-Fletcher, Michelle Saemann, Sharon Kohrs, Kimberly Mullins, Lindsay Davis, Moises Huaman, Angela Snyder, Kristin Weghorn, Brenda Miller, Elizabeth Costea, Lisa Schira, Romana Saeed, Helen Shelton, Kathleen Ballman, Laura Browning-Cho, Sherry Donaworth, Chris Goddard, Jeanine Goodin, Elizabeth Niederegger, Lisa Hachey, Tamara Maus, Pam Fletcher, Makayla Bishop, Victoria Straughn, Shaina Horner, Carrie Christofield, Dana Burns, Jason Mayes, Kelly Windholtz, Lisa Proffitt, Faizan Qureshi, Michelle O'Neil, Arustamyan Lisa, Sarah Trentman, Eva Whitehead, Jennifer Baer, Linda Hinds, Jaasiel Chapman, D'Vaughn House, Gary Frazier, Judy Houston, Lisa Altenau, Mary Burns, Dorice Smith, Justin Ragle, Eric Mueller, Cynthia Nypaver, Jaime Robertson, Anissa Moussa, Geronimo Feria Garzon, Sierra Bennett, Marlena Petrie	University of Cincinnati	Cincinnati, OH
Carlos A. Fierro, MD	Mazen Zari, Celia Gonzalez, Natalia Leistner, Mary Easley, Mary Provost, Krista Estrada, Ann Geier, Amy Thompson, Heather Barker, Karol Moore, Kelly Moen, Monica Atwood, Amber Wolf, Brandi Dickerson, Manyvohn Rinehart, Dina Hammine, Angela Eichler, Casey Johnson, Nathan Arthur	Johnson County Clin-Trials	Lenexa, KS
Veronica G. Fragoso, MD	Lisa Holloway, Cecilia McKeown-Bragas, Teresa Becker, Vicki Miller, Leena Mir, Elton Oliveira, Moez Talpur, Enya Rentas-Sherman, Gabriela Maria Becerra, Dewayne Hicks, Robert Krbashyan, Shakira Barr, Ashraf Jafri, Herman Ortiz, Zohair Harianawala, Chandra Tobin, Norma Gonzalez, Saji Perinjelil, Khorshid Amirkhosravi, Tracy Kowalski, Biman Goswami, Waheeda Sureshbabu, Amy Anderson, Berenice Ferrero, Simeen Khan, Chen-Ho Yang, Nazanin Zarinkamar, Scott Ward, Crystal Reese, Miyosha Lewis, Olga Konshina, Lorrian Yates.Joel Cano, Quiana Wilson, Kara	Texas Center for Drug Development, Inc.	Houston, TX

Principal Investigator	Study Team	Institution	Location
	Sikes, Diana Chehab, Joanna Quezan, Maryam Rabbani, Sadaf Batla, Abbyssinia Moges. Diego Carrington, Matthew Joseph, Laura Grissanty, Dean Jang, Dustin McFadden, Misbah Baloch, Elisa Moralez, Abdeali Dalal, Frances Saubon, William Fernandez, Jenny Toress, Blessing Felix, Zain Rizvi		
Ian Frank, MD	Annet Davis, Eileen Donaghy, Nicole Sundo, Juan Ramirez, Laura Schankel, Dana Brown, Katharine Bar, Dana Brown, Christopher Chianese, Gillain Constantino, Dovie Watson, Kathleen Degnan, Helen Koenig, William Short, Petra Alexander, Eileen Mergliano, Jie Ho, Michele Wisniewski, Debora Dunbar, Liani Santini-Lopez, Rosemarie Kappes, Angela Cabassa, Tammy Chen, Berry SotoVega, Deborah Kim, Devon Cliett, Kate Kearns, Jillian Baron, Vivian Leung, Florence Momplaisir, Sarah Wood, Tameka Matthews, David Metzger, Richard Tustin	University of Pennsylvania	Philadelphia, PA
Sharon E. Frey, MD	Irene Graham, Getahun Abate, Daniel Hoft, Heather Douds, Cassandra Zehenny, Joan Siegner, Helay Hassas, Kim Cooper, Shirley Dettlebach, Sabrina DiPiazza, Carol Duane, Linda Eggemeyer-Sharpe, Lauren Foreman, Jerry Hutter, Ryan Kerr, Kate Liefer, Tracy Montauk, Karla Mosby, Janice Tennant, Nicole Purcell, Kiana Wilder, Kathleen Chirco, Sharon Irby-Moore, Kathleen Koehler, Melissa Loyet, Thomas Pacatte, Susan Stewart, Azra Blazevic, Tamara Blevins, Chase Colbert, Christopher Eickhoff, Lainey Mejia Jauregui, Keith Meyer, Krystal Meza, Amanda Nethington, Huan Ning, Brittany Williams, Mei Xia, Yinyi Yu, Stanley Doublin, Mary Pat Eastman, Eric Eggemeyer, Mikayla Frye, Michelle Harris, Aleshia McCoy, Donna Duncan, Gwendolyn Tatum, Nicole Purcell, Kiana Wilder, Tammy Grant, Claudia Castillo Paredes, Rong Hou, Jin Wang, Qian Wang, Sarah George	Saint Louis University	St. Louis, MO
Cynthia Gay, MD	David Wohl, Joseph Eron, Jr., Andrew Thorne, Michelle Floris-Moore, Christopher Hurt, David Wohl, Chidinma Okafor, Janette Goins, Ulrike Adam, Ekundayo Nylander-Thompson, Anna Furlong, XinHong Ao, Kathy Guerrero, Melinda Hart, Kathleen Loeven, Rachael Mossey, Esther Speight, Rachel White, Chloe Twomey, Kristen Gray, Miriam Chicurel-Bayard, Susanne Henderson, Patti Vasquez, April Welch, Camille O'Reilly, Maureen Furlong, Noshima Darden-Tabb, Elizabeth DuBose, Marie Oriol, Dynesha Perry, Maria Stetson, Maria Bullis, Shelby Turner, Ebony Harrington, Michael Herce, Suzanne Blevins, Alexander Bradley, Susan Pedersen, Becky Straub, Sandra Barnhart, Felicia Barriga Munante, Nazneen Howerton, Tevnan Keller, Mandy Tipton, Abigail Riddick, Kristi Kirkland, Maggie Harman, Tania Hossain, Centhla Washington, Erin Hoffman, Carolina Pastrana Medina, William Johnson, Samantha Earnhardt, Amy James Loftis, Catherine Kronk, Yaa Ofori-Marfoh, Julie A Nelson, Nicole Maponga, Lina Rosengren-Hovee, William Zhao, Jennifer Thompson, Sarah Law, Holly Milner, Jonathan Oakes, Rachel Cook, Erin Cardot, Oesa Vinesett, Victoria Rucinski, Joy Wannamaker, Tanailly Giralt Smith, Eliza DuBose, Chidinma Okafor	University of North Carolina at Chapel Hill	Chapel Hill, NC
Richard M. Glover, II, MD	Stacy Slechta, Troy Holdeman, Robyn Hartvickson, Amber Grant, Jennifer Bennett, Lindsey Brewer, Janelle Brown, Kelsey Burden, Melissa Burton, Brianna Burton, Jordan Danby, Sheri Duncan, Amber Grant, Robyn Hartvickson, Lisa Hemmelgarn, Sherry Henning, Jeri King, Riley King, Colton King, April Kitterman, Shannen Lassiter, Cayla Lawless, Janna Martinez, Ragene Moore, Marissa Mueller, Aaron Nguyen, Justin Phillips, Jordan Reheis, Rebecca Ring, Katherine Saengerhausen, Shannon Thomas, Dylan Thomas, Cindy Thome, Denae Villines, Amber Wenzel, Eilleen Wilbert, Avi Woods, Caressa Presley, Brianna Newport, Olivia Allen, Miranda Santiago, Cheryl Sauerwein, Jill Longstaff, Sadie Allen, Candace Heckart	Alliance for Multispecialty Research	Newton, KS
Gregory Mark Gottschlich, MD	Melissa Gottschlich, Steven Anderson, Gregory Mark Gottschlich II, Mary Woeste, Kate Harden, Cindy Young, Michael Pordy, Audrius Ruksenas, Lacy Baird, Kim Krogman, Lori Stanton, Melissa Fuson, Mason Urban, Christine Watson, Richard Powell, Mary Smith, Jacob Sekinger, Diamond Russell, Nicole Lim, Mylene Asmar-Rios, Yusef Museitif, Craig Mitchell, Tarik Whitham, Zachary Rutledge, Troy Porter, Andrea Newlands, Jami Ramsey, Mary Frances Curry, Nishay Holloman, Crystal Barket, Michelle Spear, Shelley Mahan, Taeleigha Greene, Zachary Eardley, Gen Moussa, Mary Ann Gottschlich	New Horizons Clinical Research	Cincinnati, OH
Sinikka L. Green, MD	Julie Hamilton, Alex Fuller, Jeanette Dickhaus, Colleen Jacobson, Triny Cooper, Michelle Jackson, Taylor Evans, Tabitha Judd, Kathryn Alexander, Megan Rosallo, Sikhongi Phungwayo, Robin Dotson, Dana Finley, Michael Vasquez, Cyndi Foster, Gregg Lucksinger, Sarah Smiley, Jayasree Krishnankutty, Ray Coon, Grishma Dhimmer, Melanie Wilkerson, Tatum Shawver, Marcedes Coffman, Devin Teal, Laura Crenshaw	Advanced Clinical Research	Cedar Park, TX
Carl P. Griffin, MD	William Schnitz, Andrea Romero, Kim Hamilton, Raymond Cornelison, Angela Genovese, Shelly Brunson, April Green, Lacey Dietz, Kim Calloway, Chris Hyatt, Destiny Heinzig-Cartwright, Chalimar Rojo, Sharee Wright, Kathi Shaw, Michael Pojezny, Avery Keller, Krystal Hightower, Dalia Tovar, Shanda Gower	Lynn Health Science Institute	Oklahoma City, OK
Milton Haber, MD	Maria Candelario, Martha Bunnell-Pollak, Lauren Wade, Jackie Ziegler, Deena Ramirez, Perla Avalos, Maria Drada, Jasmine Ali, Jessica McDowell, Kehinde Busari, Patricia Church, Ronald Meza, Marco Vela, Esteban Zurita, Chris Connolly, Ruben	Laguna Clinical Research Associates	Laredo, TX

Principal Investigator	Study Team	Institution	Location
	Del Bosque, Alisha Lutat, Chelsea Fleming, Brett Potthoff, Anita Suri, Cynthia Priester, Brenda Hernandez, Veronica Procasky, Eva Cerreta, Matt Honold, Melinda Rodriguez, Maria Regalado, Jordan Stauffer		
Greg Hachigian, MD	Michael Cancilla, Ricardo Castellanos, Angela Cuellar, Yaman Darmarathne, Shaila Faulker, Yana Gordeyeva, Michelle Hisey, Ashley Jungsten, Kristin Kiersey, Pawandeep Nagra, Nav Nagra-Kooner, Jazmin Nauta, Masaru Oshita, Kenneth Quick, Julie Raygoza, Amanny Sadek, Melisa Tinder, Jhoana Torres, Deborah Murray, Kristen Kiersey	Benchmark Research	Sacramento, CA
Laurie J. Han-Conrad, MD	Brandon Baldwin, Lucian Cappoli, Tenisha Garcia, Ella Grach, Brenda Grande, Nicolle Mendez, Natalie Moy, Matthew Musikanth, Karen Mylerberg, Brennan Opanasenko, Mark Pulera, Patti Sanchez-Emery, Mireles Sarah, Todd Simmons, Denis Tarakjian	WR-Medical Center for Clinical Research	San Diego, CA
Wayne Lee Harper, MD	Toni Bland, Lori Bridges, Lucian Cappoli, Lisa Cohen, Leah Corts, Annie Craft, James Earnhardt, Lynn Eckert, Aubrey Farray, Laura Hoer, Matthew Hong, Chris Hoyle, Jenee Jiggetts, Brian Joseph, Bradley Killebrew, Kendra Lisec, Lucie Mangala, David Musante, Adnan Nasir, Amanda Olsen, Brennan Opanasenko, Marci Parks, Marion Peoples, Katherine Schuch, Judith Shand, Sabine Ucik, Douglas Wadeson, Barbara Wheeler	M3 Wake Research	Raleigh, NC
Ripley R. Hollister, MD	Jeremy Brown, Brandy Ball, Jeremy Brown, Valerie Dyster, Dalia Jeronimo, Shelby Pickle, Michael Pojezny, Melody Ronk, Kathi Shaw, Bobbi Shofner, Jami Wagner, Meghan York, Jill York	Lynn Institute of the Rockies	Colorado Springs, CO
Lisa A. Jackson, MD, MPH	Marilyn Nguyen, Maya Dunstan, Barbara Carste, Sarah Friend, Diana McFeters, Lynn Gross, Mohamed Ajenah, Jana Ffitch, Audra Mccoy, David Skatula, Susan Lasicka, Kimberly Brinker, Karen Sherwin, Melissa Scheer, Paula Lins, Roger Calvert, Roxanne Erolin, Stella Lee, Vi Tran, Stephanie Pimienta, Bruce Douglas, Lee Barr, Colin Fields, Erika Kiniry, Joe Choe, Janice Suyehira, Joyce Benoit, Michael Witte, Rebecca Lau	Kaiser Permanente Washington Health Research Institute	Seattle, WA
Spyros Andrews Kalams, MD	Greg Wilson, Kyle Rybczyk, Katie Crumbo, Carly Griffin, Latoya Hannah, Amy Kerrigan, Valerie Mitchell, Jenna Caserta, Mary Downey, Nicole Swindle, Shonda Sumner, Amber Massey, Trudy Sullivan, Rita Smith, Cindy Nochowicz, Eric Olson, Christian Warren, Josh Simmons, Dana King, Gwendolyn Rees, Matt Donio, Jesse Case, Keith Richardson, Jarissa Greenard	Vanderbilt University Medical Center	Nashville, TN
Colleen Kelley, MD, MPH	Valeria D. Cantos, Sheetal Kandiah, Carlos del Rio, Christina Bacher, Hannah Huston, Juliet Brown, Divya Bhamidipati, Nithin Gopalsamy, Brittany Lynn Speigel, Elizabeth (Betsy) Hall, Brandon Spratt, Kiran Dhillon, Caitlin Moran, Michael Chung, Felecia Wright, Marcia Peters, Rondell Jaggers, Vanessa Soliman, Ron Gaston, Christopher Foster, Sarah Wiatrek, Bezuayehu Mandefro, Pamela Weizel, Pamela Lankford-Turner, Anandi Sheth, John Gharbin, Catherine Abrams, Philip Powers, Paulina Rebolledo, Christin Root, Tiraje Lester, Sha Yi, Damien Swearing, Fred Ede, Isaac Perez, Kelly Likos, Meen Dhir, Aastha KC, Gabriela Georgial, Tucker Colvin, Nabeel Yar Khan, Valarie Hunter, D'Jamel Young, Felecia Atkinson	Emory University<br>Emory University – Ponce de Leon Clinical Research Site	Atlanta, GA
Christina Kennelly, MD	Jacob Coleman, Brittany Bundeff, Melissa C. Hennessey, Kenneth Owen, Caroline Wilds Wilds, Jennifer Womack, Susan Martello, Chiedza Hooker, Robert Brownlee, Melissa James, Deborah Wesley-Farrington, Lori Whiteheart, Hala Webster, David Framm, Cortney Fretz, Gwyn Gibson, Susan Donahue, Kelly Woodell, Linda McCarty, Jim Vesely, Scott Chatterton, Andrew Ottesen, Enrico Belgrave, Krishna Shah, James Chester Alexander, Brittain Callahan	Javara	Charlotte, NC
Shishir Kumar Khetan, MD	Taja Adams, Tanya Alexander, Tanya Alexaner, Sydney Barmoy, Jake Bart, Kira Bell, Ira Berger, Jemario Blackwell, Priscilla Buahin, Bounphone Chanthavong, Juliana DeVito, Azure Erskine, Brandon Essink, Laura Falcone, Debra Gabrielson, Beau Garland, Barb Geiger, Tiana Oliver, Courtney Heisey, Sucharita Katikala, Andrew Kimball, Heather Lang, Jeanette Lee, Asefa Mekonnen, Devan Myers, Kimberly Nieves, Allison O'Brien, Oyebisi Olanrewaju, Nicole Osborn, Adunola Oshiyoye, Rahul Patel, Alan Pollack, April Poole, Collin Smith, Kathryn Stoddard, Chao Wang, Sean Whelan, Jonathan Whelan, Graciela Zapata, Nan Zhai	Meridian Clinical Researc	Rockville, MD
Murray A. Kimmel, DO	Alexa Diec, Ann Riley, Bette Denmat, Bram Swarr, Christina Raidl, Dania Billman, Denise Dixon, Donald Dawson, Elaine Crudo, James Crowley, Katrina Carlson, Kaylie Worzick, Laura Worth, Lisbeth Gordon, Marion Oliver, Robert Holt, Simmy Pinto, Taylor Atkinson, Traci Mitchell, Lana Ghomrawi, Norma Rokoff	Optimal Research	Melbourne, FL
Judith L. Kirstein, MD	Jared Bradshaw, Krista Forster, Jeanette Dickhaus, Marcia Bernard, Erica Sanchez, Nikki Abels, Cynthia Kunakom, Vanessa Vandergoot, Jessica Fisher, Carol Remigio, Jourdan Manfred, Frederick Lloyd, Tiffany Williams, Clarisse Baudelaire, Lovette Cherelle, Nolan Mackey, Alan Valenzuela, Theodore Wyman, Alyssa Taber, Karen Myers, Craig Koch	Rancho Paseo Medical Group	Banning, CA
Michael J. Koren, MD	Shannon Trull, Amanda Elwood, Mary Strickland, Ivy Gulliermo, Chistopher Ganzhorn, Sonia Gerardo, Taylor Johnson, Victoria Kaposchansky, Cassie Lawler, Laura Little, Amanda Pratt, Sheldon Warren, Andrea West, Emery Noles, Nathanial	Jacksonville Center for Clinical Research	Jacksonville, FL

Principal Investigator	Study Team	Institution	Location
	Grant, Jillian Agnew, Lori Alexander, Brenda Anderson, Deirdre Arrington, Sara Benner, Lisa Carl, Allison Crain, Nafisa Ishaku, Robert Nix, Sharon Smith, Amber Devries, Sandy Salceiro, Opara Chukwudi, Mikaela Karney-Trull, Ramil Castillo, David Graham, Gail Lowe, Alexander Hill, Carolyn Tran, Jeffry Jacqmein, Darlene Bartilucci, Alpa Patel, Janet Garvey, Mitchell Rothstein, Kenneth Aung-Din, Margaret Gannaway, Arman Mughal, Sandra Fuit, Jolenne Wolfer, Erin Schelhorn, Jacob Wolfer, Madison Martinez, Melissa Parks, Patricia Neal		
Karen L. Kotloff, MD	Matthew Laurens, Milagritos Tapia, Lisa Chrisley, Cheryl Young, Barbara Albert, Robin Barnes, Shernel Barrett, Andrea Berry, Melissa Billington, Shannon Bittner, Colleen Boyce, Faith Pa'Ahana Brown, James Campbell, Regina Carpenter, Jamonie Carter, Ginny Cummings, Brenda Dorsey, Jorge Flores, DeAnna Friedman-Klabanoff, Shirley George, Nancy Greenberg, Hassan Haji, Elizabeth Hammershaimb, Susan Holian, Leslie Howe, Myounghee Lee, Alyson Kwon, Kirsten Lyke, Alma Valle Maldonado, Jennifer Marron, Kaitlin Mason, Monica McArthur, Rosa McBryde, Sherry McCammon, Sandra Molina, Kathleen Neuzil, Daniele Nitkowski , Justin Ortiz, Rekha Rapaka, Mardi Reymann, Toni Robinson, Wanda Somrajit, Mark Travassos	University of Maryland, School of Medicine	Baltimore, MD
Mark E. Kutner, MD	Amanda Colina, Isett Caro, Frances Beltran, Jessie Downs, Jonathan Fernandez, Mariete Renden, Mirnaya Mujica-Alabaci, Susel Figueredo, Yanelis Dominguez, Jaime Blandon, Bryan Ruiz, Leidy Montoya, Edgardo Rodriguez, Jessie Downs, Jason Rothscheld, Janett Acle, Yaime Martinez, Soraya Ricardo, Maria Hernandez Moran, Eloisa Guerra, Heidie Perez, Claudia Rodriguez, Victoria Moreno, Vanessa Hechavarria, Saray Carvajal, Daniel Lopez, Carlos Ivircu, Neiner Enriquez, Paola Garcia, Chris Hoyle, Marianela Carvajal, Janet Mendez, Edisleidy Mesa, Marco Ramirez, Dalila Del Valle, Jennifer Ortega, Yeni Hernandez, Jhobana Vargas, Carmen Amador, Juan Delgado, Maury Santos, Meredith Arguelles, Leyanis Coello, Vanessa Ansorena, Jorge Caso, Stacy Machado, Raydel Valdes, Giann Lightbourn, Dayami Dovales, Alain Chang	Suncoast Research Group	Miami, FL
Mimi Van Der Leden, MD, PhD	Chrishea Harvey, Tricia Oyeyemi, Aicha Moutanni, Stephanie Melton, Peta-Gay Jackson Booth, Jennifer Yoon, Gloria Kim, Atanas Filev, Francis Uwandi, Meyling Lopez, Janice Spreitzer, Courtney Gennes, Xiangfei Cheng, Matthew Van Sickle, Nick Bart, Brianne Okunji, Frank Maloba	Optimal Research	Rockville, MD
Michael L. Levin, MD	Brennan Opanasenko, Yajaira Ramos, Shonda Lester, Rebecca Boucher, Shawn Harrell, Shon Boucher, Patti Sanchez, Nina Scharbach, Alex Sanchez, Shyane Raniello, Wendy Guerra, Krystal Tyner, Kimberly Temple, Ruby Ortiz, Daniel Terreault, Amy Kill, Jade Odynski, Adolfo DeLeon, Debbie Carter, Eduardo Rodriguez, Julia Gass, Sara Esparza, Sierra Dansbee, Tammy Harrison, Marcy Kulic, Lucian Cappoli, Mora KIm, Matthew Fenner, Heather Jimenez, Shraddha Dubal, Julie Hussey	WR-Clinical Research Center of Nevada	Las Vegas, NV
Michael Lewis, MD	Nancy Mohler, Mai Pham, Ron Waldorf, Elham Ghadishah, Samantha Feril, Stella Lee, Dzuyen Nguyen, Ruoxiang Wang, Justine Velandria, Benjamin Dreskin, Joseph Yusin, Lauren Vigil, Sara Wong, Suchi Tiwari, Joseph Pisegna, Sunita Dergalust, Wayman Lee, Krissa Caroff	VA Greater Los Angeles Healthcare System	Los Angeles, CA
Gregg H. Lucksinger, MD	Jaleh Ostovar, Craig Koch, Danuel Hamlin, Kelly Chase, Jeanette Dickhaus, Edward Kerwin, Frederick Forde, Allison Alvord, Dawn Stewart, Dan Hamlin, Kevin Parks, Ryan Israelsen, Kary Kelly, Tiffany Smith, Melissa Myers, Ryan Rackley, Audrey Kuehl, Savannah Peterson, Hannah Hall, Jay Weisbart, Alison Dodenhoff, Emily Kelly	Velocity Clinical Research	Medford, OR
Mary Beth Manning, MD	Carol Salango, Alec Ireland, Lisa Hoagland, Jeanette Dickhaus, Toby Briskin, Joan Rothenberg, Michael Gaston, Sharita Tedder-Edwards, Denise Roadman, Megan Sokolowski, Tina Shickluna, Katherine Bielanski, Samantha Hood, Talia Chandler, Brianna Arman, Melinda DeLong, Naqib Ahmad, Karly Tarase, Jade Svoboda, Lisle Merriman, Melisa Sebera, Emma Landskroner, Amy Maroun, Brooke Glivar, Jennifer Gaston, Sarah Dzigiel, Cassiandra Uminski, Karol Sabol, Devan Patel, Nick Zarbo, Briana Jackson, Brian Sharpe, Nicole, Baitt, Kaitlyn Duffy, Gabrielle Jacobs, Ann Czuprun, Tracee Cash, Diamond Ivey, Kaitlyn Rubell	Rapid Medical Research	Cleveland, OH
Kristen Marks, MD	Grant Ellsworth, Tina Wang, Timothy Wilkin, Mary Vogler, Carrie Johnston, Marshall Glesby, Roy Gulick, Ole Vielemeyer, Rebecca Fry, Todd Stroberg, Caitlin Rhoades, Noah Goss, Shaun Barcavage, Valery Hughes, Jonathan Berardi, Caroline Greene, Sarah Galloway, Caique Mello, Ashley Machado, Mia Crowley, Monique Williams, Katherine Fee, Elizabeth DeJesus, Andrew Yu, Minkyung Lee, Susan Herder, Mary Ann Zweibel, Patrice Weller, Antonio Rivera-Lopez, Edward Kenny, Hetal May, Natella Fridman, Parul Shah, Ruby Lee, Venus Fernandez, Victoria Lesina, Celine Arar, Byron Bullough, Kinge-Ann Marcelin, Brian Mangano, Jessenia Fuentes, Jiamin Li, Genessi Rodriguez, Catherine Jerry, Nadi Islam, Liqun Cai, Wayne Burns, Akinbayo Caulcrick, Andrika Thomas, Barbara Batog, Guoan He, Sara Yoder, Tamara Crowder, Gianna Resso, Sophia Alvarez, Tahera Begum, Elizabeth Connolly, Roxanne Rosario, Paul Kim, Steven Wang, Vasilika Koci	Cornell Clinical Trials Units - Weill Cornell Chelsea and Uptown	New York, NY

Principal Investigator	Study Team	Institution	Location
Judith Martin, MD	Alejandro Hoberman, Timothy Shope, Gysella Muniz, Sonika Bhatnagar, Kumaravel Rajakumar, Anne-Marie Rick, Peri Unligil, Jennifer Nagg, Melissa Andrasko, Mary Ann Sieber, Jennifer Opal, Lalicia Roman, Spenser Kinsey, Michelle Burke, Matthew Lee, Dominic Kramer, Linette Milkovich, Emily Dougherty, Emily Carney, Shannon Mance, Nader Shaikh, Diana Kearney, Jamie Fries, Lisa Vavro, Shayla Goller	UPMC University Center	Pittsburgh, PA
John W. McGettigan, Jr., MD	Walter Patton, Jennifer Schnider, Riemeka Brakema, Heeten Desai, Mikell Brett Karsten, Patricia Jalomo, Cindy Finch Benoy, Karin Choquette, Jonlyn McGettigan, Yvonne De Los Reyes, Melissa Cozzens, Amanda Hermosillo, Cindy Montgomery, Susan Tarwid, Annette Elzy, Tianna Young, Saysamone Banks, Cristina Fernandez, Damaris Atondo, Zoe Sesma, Norma Barrientos, Maggie Tono, Kisha Adams, JoAnn Wilkins, Arianna Bermudez, Carol Sayer, Julie McDowell, Angelina Navarro, Mercedes Sullivan, Crystal Mata, Sheldon Gingrich, Aaliyah Sestiaga, Gia Longo	Quality of Life Medical & Research Centers	Tucson, AZ
Mark Montgomery McKenzie, MD	Tiffany Jewell, Zackery Harmon, Michael Elizabeth, Christy Sweet, Teresa Deese, Catherine Schon, Misti Earwood, Lou Cappoli, Brennan Opanasenko, Lisa Guider, Michelle Forgey, Justian Jarrett, Rachel Scott, Elizabeth Michael, Erica Osmundsen, Andrew Wood, Shelly Brooks, Gisela Heintz, Lilian Nukuna	WR-ClinSearch	Chattanooga, TN
Vicki E. Miller, MD	Sajjad Naqvi, Soofia Masood, Fredric Santiago, Sonia Guerrero, Subhash Koneru, Nirja Shah, Andrea Torres, Ramani Gali, Talha Baig, Heather Leary, Afifah Ayub, Nayab Goher, Patti Tate, Reagen Reed, Muhammad Irfan, Amy Starr, Alefiyah Motiwala, Julia Kenny, Victoria Aguilar, Jessica Arguijo, Insiya Valika, Victoria Aguilar, Jagruti Patel, Anna Pena, Faryal Mahmood, Blanca Gomez, Nancy Torres, Kristyn Latil, Tarori Mark, Laura Djampou, Lindsey Kueng, Marianne Tadros, Mohammad Millwala, Monica Murray, Murtaza Marvi, Shivani Shah, Vanessa Gonzalez, Zohair Harianawala, Zainab Rizvi, Ambily Dileep, Jaquelyn Gonzales, Ragen Powell, Carolina Deandres, Syed Fahad Ali Kazmi, Sandra Natalia Perez, Shannon Amacker, Shiela Varghese	DM Clinical Research	Tomball, TX
Gowdhami Mohan, MD	Rodolfo Barrera, Emma Partin, Kelly White, Ashley Rochester, Charles Thompson, Stefanie Tyson, Ashten Sheriff, Alyssa-Kay Peay, Kayla Corn, Barbara A. Richardson, Kristin Miller, Steven Clemons, Cameron King, Emma Partin, Gary Clemons, Brianna Starr, Danyel Johnson, Taylor Davis, Niki Tyson	VitaLink Research	Anderson, SC
Kathleen M. Mullane, DO, PharmD	David L. Pitrak, Cheryl Nuss, Karen Cornelius, Randee Estes, Amy Luckett, Michelle Moore, Judi Pi, Stephen Schrantz, Jill Stetkevych	University of Chicago	Chicago, IL
Joseph Lee Newberg, MD	Mary Reyes, Nicole Leahy, Victoria Andriulis, Herbert Whinna, Patricia James, Lana Ghomrawi, Carole Kempfer, Miriam Arroyo, Maria Castro, Anna Maddox, Reuben Martinez, Jacquilyn McCormick-Burks, Laura Pearlman, Rosalinda Vazquez, Shaheera Suleiman, Neha Atal, Rosalind Vazquez	Synexus Clinical Research	Chicago, IL
Richard M. Novak, MD	Regina Harden, Maria Schwarber, Michael Pacini, Rebeca Gansari, Margie Villarreal, Stephanie Martin, Michelle Lee, Richard Morrissy, , Taylor Ellis, Samuel Rene, Tara Cobbs, Claudia Preciado, Scott Borgetti, Maximo Brito, Olamide Jarrett, Mahesh Patel, Tracy Cable, Charity Ball, Maryann Holtcamp, Rodrigo Burgos, Sarah Michienzi, Emily Drwiega, Mikayla Johnson, Fischer Herald, Benjamin Ladner, Minseung Chu, Carolyn Dickens, Alfredo Mena Lora, Stockton Mayer, Andrea Wendrow, Habiba Sultana, Nanu Nunwar, David Chan, Marla Schwarber, Khandaker Anwar, Mahmood Ghassemi, Md Ruhul Amin, Doris Carroll, Rosa Valencia, Michelle Agnoli, Elena Llinas, Samuel Rene, Liam Morrissy, Adrian Raygoza, Addis Mekkonnen, Lisa Lindemann, Daniel Meslar, Karen Pacini, Corey Ringhisen, Amy Kennedy-Krage, Claudia Miller, Lorna Sanchez McCann, Gizelle Alvarez, Nia Moragne-Oneal, Nusirat Williams, Ian Feather, Nikki Griffith, Wardrick Nealon, Renyce Powell, Nila Safaeian, Monica Gingell, Diana Bahena, Gerald Beck, Brad Farrington, Rod Reyes, Monica Wilson, Juline Wondrasek, Kimberly Shapiro, Shannon Whitted, Victoria Roehl, Braulio Carrasco, Michael Chen, Olivia Murray, Yasiel Lacalle, Tessa Eckley, Anna Schluckebier, Kevin Cao, Elise DeBruyn	University of Illinois at Chicago - Project WISH	Chicago, IL
Paul Joseph Nugent, DO	Leonard Singer, Jennifer Jones, April Smith, Georgettea Geuss, Lana Ghomrawi, Christine Bennett, Norma Blevins, Linda Brotherton, Michele Byrd, Krista Doss, Victoria Holden, Christine Hull, Jean Montgomery, Nancy Cipollone, Savanah Torline, Brandon Brown, Meagan Thomas, Katie Ziska, Dana Sias, Hannah Wagner	Synexus Clinical Research	Cincinnati, OH
Jeffrey Scott Overcash, MD	Hanh Chu, Kia Lee, Karla Zepeda, John Rodriguez, Adam Prince, Yashveer Dubbula, Elizabeth Tomatsu Michael Voskanian, Crystle Rajania, Stephanie Ramirez, Claudia Camacho, Lauren Arnett, Kecia Darbeau, Ashley Smith, Kimberly Quillin, Cesar Ramirez, Daniel Robitaille, Erica Sanchez, Allie Davis, Michael Waters, Pat Kappen, Valerie Horne, Thao Vuong, Andrew Dennis, Nikki Abels, Dominique Panis, Richard McQuaid, Whitley Harbison, Erika Trujillo, Andrea Garcia, Jose Jacob Esparza, Carlos Vera, Raquel Taitingfong, Cathy Meza, He Pu, Jackielynn Smith, Shandel Odom, Zahira Nieves, Ashliegh Lindsay, Ariana Nasatka, Jose Cazarez, Nora Martinez, Angela Hunt, Antonio Delgado, Linda Vega, Angela Anorve, Erica Martinelli, Melania Riordan, Sylvia Lindholm, Gina Ciezkowski, Grecia Perez, Jacob Pineda,	Velocity Clinical Research, San Diego	La Mesa, CA

Principal Investigator	Study Team	Institution	Location
	Nathan Tyler, Ranya Salem, Amara Yilmaz, Jessica Gonzales, Zabrina Ruiz, Laura Castillo, Yajaira Contreras, Angelica Guzman, Makenna Orel, Jeffery Alvarez, Gordon Bovee, Roxana Ramirez, Joan Esquivel		
James Todd Peterson, MD	Christopher Mickelson, Madeline Maldonado, Alison Charlton, Ashley Bragg, Sean Hansen, Emily Wilcox, Colby Bostock, Megan Henry, Pam Iwasaki, Bradley Young, Katelyn Walker, Joy Nguyen, Lindsey Bevan, Megan Grimmett, Madeline Grote, Heather Littell, Natalie Bee, Alexander Clark, Shana Eborn, Susan Edwards, Dan Henry, Heather Jackson, Gerald Kelty, Issac Pena-Renteria, Jacqueline Rohrer, Jack Taylor, Brooke Barrick, Ty Henry, Anna Dansie, Kenadie Hamblin	J. Lewis Research	Salt Lake City, UT
Paul Pickrell, MD	Susan Bonner, Blaire Graham, Staci Taggart, Hussain Malbari, Tiffany Lemuz, Ethan Shotton, Andrew Bell, Megan Malek, David Pampe, Carol Ann Linebarger, Michelle Peterson, Brandi Chalman, John Luna, Elizabeth Santellanes, Christina Martinez, Lisa Johnson, Lisa Savage, Melissa Winn, Wendi McKenzie, Eileen Euperio, Stefanie Mott, Paul Menefee, Katie Caballero, Darrell O'Brien, Morgan Schulle, Kate Jurek, Olivia Hapanowicz	Tekton Research, Inc.	Austin, TX
Terry L. Poling, MD	Meenakshi (Kavya) Natesan, Patricia Contreras, Denise Hole, Avi Woods, Jill Hiebert, Melissa Burton, Olivia Eagleson, Laura Holz, Terri Ford, Cindy Thome, Terry D Klein, Gregory Greer, Diandra Henriques, Tracy R Klein, Thomas C Klein, Christa Shue, Gina Young, Brenna Sprout	Alliance for Multispecialty Research	Wichita, KS
Bruce G. Rankin, DO	Jennifer Dittman, Lora Parahovnik, Crystal Paccione, Melissa Hodges, Katina Marchione, Matt Maxwell, Any Dominy, Diana Toney, Andrea Marrafino, Laura Isbell, Leandro Fernandez, Claxton Copeland, Michelle Tutt, Adam VanDeusen, Kevin Feldman, Clark Mason, Tifany Huertas, Over Seijas, Jennifer Cline, Christian Beierschmitt, Ryan Hobbick, Jessica Gilliam, Jeanette de Leon, Iman Mencia, Daniel Layish, Vienna Bauer, Shatonia Fields, Albert Garcia, Carrie Rycort, Tasha Brocato, Marshall Nash, Samantha Watts, Amy Houck-Dominy, Angela Hammerle, Teresa Logsdon, Erika Wierzbicki, Taylor Martin, Ranie Hutchins, Fadhel Alyunis, Gail Lavine, Jeffery Hood, Robert Duran, Michelle Jones, Ginny McClanahan, Heather Jackson, Leandra Fernandez, Douglas Winter, Antonio Rivera, Amber Vasquez, Thais Truffa, Daniel Campbell, Grace Newcomb, Elizabeth Orlando, Steven Shinn, John Hill, Christina Isbell, Dhaneshwar Oomrow, Alicia Cevera	Accel Research Sites	DeLand, FL
Michele Diane Reynolds, MD	Jennifer Bashour, Robert Schmidt, Cynthia Mayeux, Uvoka Huffman, Lisa Nicholson, Jacklyn Newton, Lynn Yauch, Cathy Monroe, Kathleen Carty, Angelica Banks, Taylor Werner, Pamela Echols, Pauline Jackson, Chana Hines, Lorine Cook, Cristina Puig, Patrick Brooks, Jennifer Ruiz, Deanna Bowman, Ladina Garcia	Synexus Clinical Research	Dallas, TX
Rambod Rouhbakhsh, MD, MBA	John Johnston, Richard Calderone, Tasha Stevenson, Tameka Fortune, Brandi Pace, Adreanna Pou, Jerrica Sullivan, Yolanda Lewis, April Rouse, Tiffany Jefferson, Elizabeth Danford, Jeff Repper, Mason Boutwell, Alexycia Washington, Krista Hirth, Meagan Grabel	MediSync Clinical Research Hattiesburg Clinic	Petal, MS
Nadine Rouphael, MD	Renata Dennis, Tigisty Girmay, Michelle Wiles, Sharon Curate-Ingram, Lauren Hewitt, Alexis Ahonen, Mari Hart, Sarah Bechnak, Erin Carter, Lauren Nolan, Daniel Sans Graciaa, Geoffrey Kamau, Easton Beshears, Sy Tran, Mary Atha, Mary Bower, Ghina Alaaedine, Brandy Johnson, Jacob Usher, Eileen Osinski, Erin Scherer, C. Tae Stallworth, Stephanie Ramer, Rose Pope, Esther Park, Francine Dyer, Laura Clegg, Rebecca Gonzalez, Stacey Wheeler, Susan Rogers, Vy Ngo, Vanessa Soliman, Kristen Unterberger, Bernadine Panganiban, Christopher Huerta, Juton Winston, Ali Alvarez, Jianguo Xu, Colleen Kelley, Paulina Rebolledo, Nicholas Scanlon, Jessica Traenkner, Matthew Collins, Hollie Macenczak, Cassie Grimsely-Ackerley, Tiffany Lee, Amy Anderson, Michele Paine McCullough, Hannah Huston, Daniella Carter, Lisa Harewood, Srilatha Edupuganti, Varun Phadke, Mindee Adamson, Jeanne Allen, Debbie Bartenfeld, Lily Berz, Amy Cromwell, Sergio Cruz, Fred Ede, Monica Godfrey, Evan Gutter, Angelle Ijeoma, Sara Jo Johnson, Vinit Karmali, Dean Kleinhenz, Jennifer Kleinhenz, Alexandra Koumanelis, Maranda Leary, Tiraje Lester, Juliet Alise Morales, Shashi Nagar, Julia Paine, Dilshad Rafi Ahmed, Brittany Robinson, Amanda Rosner, Renee Silver, Trevor William Simon, Talib Sirajud-Deen, Damien Swearing, Maliya Tolbert, Pamela Turner, Chia Uzuegbunam, Claire Wan, Dongli Wang, Erika Wimberly, Jean Winter, Joy Winters, Yong Xu, Sha Yi	Emory University - Hope Clinic	Decatur, GA
Richard Rupp, MD	Amber Stanford, Megan Berman, Laura Porterfield, Gerianne Casey, Hala Ghoson, Doreen Jones, Michael Willig, Cori Burkett, Robert Cox, Amy McMahan, Diane Barrett, Kristin Pollock	University of Texas Medical Branch	Galveston, TX
Jamshid Saleh, MD	Matthew Miles, Rafael Lupercio, Vicky Martin, Marla Clark, Matthew Pohlmeyer, Ruba Zanaid, Veronica Blevins, Tara Ulberg, Carlyee Chambers, Marisol Corrales, Emily Crews, Mohamed Yassin, Sarah Sandberg, Frank Chen, Mandy Swanson	Paradigm Clinical Research Center	Redding, CA
John W. Sanders, MD, MPH	Stacy Harpe-Hall, Jesse Hopkins, Ann Schweppe, Jaymous Fayssoux, Kathryn Bender, James Peacock, Katharine Pearsall, Brandy Snyder, Deidre Knox, Megan Thorpe, Melissa Ellingson, Brittany Bundeff, Lisa Ashworth, Meredith Hiatt, Ritu Rathee, Stacy Woodliff, Brian Strittmatter, Amanda Wright, Daisy DeWeese-Gatt,	Wake Forest University Health Sciences	Winston Salem, NC

Principal Investigator	Study Team	Institution	Location
	Caryn Morse, John Williamson, Samantha Wheeler, Lori Whiteheart, Susan Donahue, James Lovette, Kaitlyn Van Leuvan, Kelly Ledbetter, Scott Chatterton, Julio Nasim, Amie Sidberry, Ashley Davis, Carter Noecker, Chie Hooker, Johanna Breenan, Sam Cable, Anna Bowman, Stephanie Boothe, Shea Overcash		
Howard I. Schwartz, MD	Carlos Valladares, Jocelyn Morrera, Yulexis Amestoy, Tori Wallenburg, Thelma Beltran, Terry Piedra, Monica Garces, Alexandra Galvis, Wanda Delgado, Catherine Casas, Lesly Miguel Sosa, Vivian Rosales, Jose Fernando Henriquez, Mikael Yaniz, Beatriz Rivera, Peter Ventre, Gabriella Huyke, Maria Companioni, Jessie De Vega, Brianna Gamez, Stephanie Diaz, James Jean-Mary, Americo Padilla, Nikita Notise, Yorlina Luquetta, Monifa Wilson-Morris, Kenia Gutierrez, Roilan Garcia, Karla Pentzke, Leyda Valentin, Lazara Novas, Marilein Camacho, Jazmin Henfield, Laymis Alvarez, Myriam Rosado, Maxine Bryant, Maria Pinero, Laura Raucci, Francisco Ramirez, Angelic Gamez, Mailin Perez, Yasmin Baddour, Hary Leon Joseph, Yaquelin De la Cruz, Dunia Torres, Rosaidaliz Carreira, Chanella Garcia, Surisaday Mederos, Jose Muniz, Karenda Plotka, Sara Gomez, Maria Soto, Cathy Cruz, Nelia Sanchez-Crespo, Jennifer Schwartz, Barbara Corral, Matthew Muniz, Dayana Deltejo, Ana Castro, Reem Hassan	Research Centers of America	Hollywood, FL
Nathan Segall, MD	Michelle Sowell, Nancy Levine, Erynn McKinley, Hannah Smith, Karen Hickson, Elizabet West, Patrizia Greene, Jon Finley, Mildred Stull, Susan Jones, Jennifer LeBrun, Pamela Talbott, Kwannda Whatley, Jeffrey Jones, Michelle Binns, Donna Toepfer, Cynthia Steele, Grace Newville, Gillian Waite, Cynthia Pinckney, Karen Yangapatty, Kiara Tyner, Kimberly Cobb, Kourtney Richardson	Clinical Research Atlanta	Stockbridge, GA
William Seger, MD	Kimberly Pullen, Jean Seignon, Anthony Kim, Mohammed Antwi, Allison Green, Lizzy Seger, Elizabeth Boydston, Abdur Rafay Qadri, Deborah Devlin, Tasha Todd, Oluwatosin Akingbala, Alma Guel, Tisha Davis, Melody Dufrene, Samantha Loudermilk, Virginia Loudermilk, Crystal Starr, John Villegas, Ben Seger, Katherine Hollie	Benchmark Research	Fort Worth, TX
Neil Parmanand Sheth, MD	Kenneth Stell, David Beckett, Enitt Gonzalez, Donna McGunigal, Amanda Burns, Nancy Wood, Shelley Miceli, Christina Avila, Rebecca Baker, Laura Vigliotti, Sarah Kading, Samer Salama	Synexus Clinical Research	Glendale, AZ
William B. Smith, MD	Richard L Gibson, Jennifer Winbigler, Elizabeth Parker, Madison Watts, Suzann Cloninger, Talya Thomas	Alliance for Multispecialty Research	Knoxville,TN
Joel Solis, MD	Martha Carmen Medina, Xavier Morales, Hank Heller, Blake Torrence, Joanna Gurrola-Mahoney, Cynthia Bueno, Heather Holloway, Irving Salinas, Joel Perez, Paola Garcia, Erica Canales, Blanca Urbina, Brancisilio Gutierrez, Carolina Cantu, Chelsea Vargas, Cindy Vasquez, Cody McIntire, Gabriela Gutierrez, Hugo Sosa, Irvin Munoz, Jessica Estrada, Jonna Lopez, Kaegan Knox, Mirella Melendez, Natalia Valle, Natalie Echavarria, Nicole Litton, Amber Victor, Nancy Torrence, Madhu Shreya, Mathew Maran, Asfak Alam, Westly Keating, Tara Green, Devora Torrence, Gerardo Sedas, Shruti Konda, Prem Jangam, Mario Echavarria, Alejandro Silva, Anne McNulty, Daniel Contreras, Daniel Gomez, Edgar Garcia, Elizabeth Weber, Luis Lopez, Samuel Ramirez, Kayla Lopez, Pedro Penalo, Angel Salinas, Jaime Solis, Shannon Moyer, Aryana Ibarra, Guadalupe Gurrola, Jenna Anastasiades, Uchechi Ehiemua, Sara Solorzano	Centex Studies, Inc.	McAllen, TX
Stephen A. Spector, MD	Amaran Moodley, Jill Blumenthal, Baharin Abdullah, Christina Addington, Juan Carlos Alcantar, Deyna Arellano, Bernadette Cale, Brendan Costello, Tammelita Cotlon-Pineda, Fanny Delebecque, Karen Deutsch, Aram Dimayuga, Son Do, Yasmeen Esshaki, Aileen Everhart, Cindy Ewing, Veronica Figueroa, Medardo Gaytan, Crystal Groom, Carolyn Hernandez, Heather Huitema, Benjamin Hull, Sylvia Isaac, Jaclyn Jaskowiak, Cindy Knott, Leander Lazaro, Thuan Le, Megan Loughran, Michelle Madey, Rosalva Martha-Patten, Colleen McLellan, Jeff Ledford-Mills, Asami Mimura, Patty Moraes, Jennifer Morales, Jessica Nasca, Phirum Nguyen, Marielys Padilla-Martinez, Dennis Perpetua, Mike Pizza, Shannon Ransom, Emily Rizo, Carlos Rojas, Thaine Ross, Marie Sagrado, Eugene Sato, Lisa Stangl, Ji Sun, Nancy Tang, Mina Trivedi, Rodney Trout, Donna Voss, Lindsey Woronicz	University of California, San Diego	La Jolla, CA
Cynthia Becher Strout, MD	Rica Santiago, Yvonne Davis, Patty Howenstine, Alison Bondell, Jaime Robertson, Anissa Moussa, Geronimo Feria Garzon, Sierra Bennett, Marlena Petrie	Coastal Carolina Research Center	Mount Pleasant, SC
Shobha Swaminathan, MD	Amesika Nyaku, Tilly Varughese, Rondalya Deshields, Michelle L DallaPiazza, Elise Lewis, Jennifer Punsal, Mario Portilla, Malithi Desilva, Christina Daliani, Susana Rivera, Aidan Ziobro, Andressa Rebellatto, Brian Murloy, Christina Ninan, Ernest Pianim, Eunice Wang, Merit Henen, Muhammad Usman, Rebecca Kim, Shiao Wang, Gener Eric Cruz, Bethany Birago, Joyell Arscott, Dina Meawad, Christie Lyn Costanza, Francesca Escaleira, Zoraida Cruz-Barahona, Jared Khan, Valeria Cadorett, Jamir Tuten, Travis Love, Eric Asencio, Sukhwinder Singh	Rutgers New Jersey Medical School	Newark, NJ

Principal Investigator	Study Team	Institution	Location
Ramy Joseph Toma, MD	Olivia Graves, Josiah Robinson, Patricia Hammonds, Lana Ghomrawi, Kara Quinnelly, Shaun O'Conor, Michael Lambe, Rachell Stewart, William Kirby, Pink Folmar, Rachel Culbreth, Heidi Leblanc, Julie McDaniel, Rian Montgomery, Andrea Woodle, Samantha Williams, Hunter Russell, Shereen Lowe, Maureen Mayer, Hollis Ryan, Elaine Reese	Synexus Clinical Research	Birmingham, AL
Timothy P. Vachris, MD	Mark Hutchens, Stephen Daniels, Margaret Wells, Sandra Clancy, Rebecca Martinez, Jessica Buot, Merissa Daugherty, Julie Hamilton, Kimberly Hernandez, Ashli Alejandro, Amy Collins, Monique Gawlik, Patricia Johnson, Maria Moreno, Ashley Washington, Tina Rountree, Daniel Dore, Ravi Davuluri, Ashlee Brunaugh, Jorge Martinez, James Hermon, Vianai Carreno, Mia Rountree, Colleen Coelho	Optimal Research	Austin, TX
Keith William Vrbicky, MD	Charles Harper, Chelsie Nutsch, Wendell Lewis III, Cathy Laflan, Linden DeBoer, Kayla Andal, Misty Appeldorn, Jenniger Grebe, Russell Herstein, Catherine King, Samantha Wieseler, Alisha Kiepke, Christy Lee, Kelsey Kelley, Kelli James, Ashley Frisch, Courney Green, Taysha Hingst, Jeni Hoppe, Kimber Breeden, Debra Gabrielson, Ginny McNew	Meridian Clinical Research	Norfolk, NE
Larkin T. Wadsworth III, MD	Ashley Dale, Christy Schultz, Rebecca Munsch, Anya Penly, Liz Garner, Stephanie Tesson, George Cherniawski, Angie Kean, Dan Reed, Courtney Kubiak, Maureen Dempsey, Heather Cherniawski, Breanna Galibert, Kristin Branson, Laura Hartupee, Karen Knapp, Horacio Marafioti, Lyly Dang, Jennifer Berry, Lauren Clement, Megan Dandurand	Sundance Clinical Research	St. Louis, MO
Jordan L. Whatley, MD	Patricia Whatley, Christopher Dedon, Anika Payne, Amie Shannon, Kristen Losavio, Nicole Harrell, Mary Margaret Dobson, Lindsey Hall, Chaney Bennett, Crystal Rowell, Mimi Dimmick, Amy Thomassie, Kimber Breeden, Cody LaFleur, Makaylea Truitt, Taryn Collett, Emily Best, Alexandra Caillouet,	Meridian Clinical Research	Baton Rouge, LA
Judith White, MD	Amy Edridge, Chelsea Montalvo, Eugenia Clark, Lisa Russell, Zahra Somji, Lesli Leimer, Robert Meyer, Christine Murphy, Prity Patel, Sejal Patel, Ruben Moliere, Samantha Merveillard, Yarnick Mirjah, Bryn Walls, Joey Cruz, Aaron Cooper, Jessica Bienaime, Ashley Gilcrist, Alisa Petit, Tyler Knightly, Kimberly Stokes, Christina Rosario, Talhia Matos, Ilona Boggs, Nicholas Weber, Felix Busot, Linda Colon, Heather Gillenwater, Cristina Kaplun, Melissa Caputi, Shayna Siplin, Daminee Shah, Samuel Martin, Alexis Waldorf, Vihar Upadhyay, Adolfo Henriquez, Saskia Singh, Maria Roberts, John Caporelli, Shirley Salvador, Quevina Scarver, Vanessa Garcia, Taylor Moore, Jayasen Singh, Curshinda Galvin-Burch, Mary Kesner, Jasmin Gil, Shay Gray, Steven Monsegur, Michele Steinmetz, Michael Lambe, Heather Powell, Sandra Torres, Shaban Katbeh, Taylor Wilson	Synexus Clinical Research	Orlando, FL
Priyantha N. Wijewardane, MD	Natalie Johnson, Martha Evans, Sondra Wright, Richard Pellegrino, Lastida Burns, Natasha Williams, Haylee Rowe, Kayla Graham, Amanda Horn, Eric Bravo, Jeffrey Thessing, A. Michele Maxwell, Amy Cooper, Lauren Evans, Tonya Cato, Haylee Tucker, Lesa Gann, Hannah Jones, Amanda May, Tiffany Walker, A. LeiAn Diaz, Laura Khalil, Lydia Purcell, Timothy Campbell, Charlotte Garcia-Velez, Andrea Scarborough, Beatrice A. Miller, Keith Bracy, Aujania Thompson, Cassandra Johnson, Krishana Day, Freddie Hicks, Jamie Pettus	Baptist Health Center for Clinical Research	Little Rock, AR
Barton G. Williams, MD	Flo Abbott, Nicole Burton, Alice Cipollini, Madison Croucher, Philip Dattilo, Erin Harrelson, Kelsey Heston, James Ingram, William H Jones, Karla Lane, Brandy Lowman, Evan Lucas, Megan Marles, Morgan Mathis, Angie Northcott, Clyda Pasquantonio, Alyssa Valente, Ciara Winders, Stephanie Graham	Trial Management Associates	Wilmington, NC
Marcus J. Zervos, MD	Paul Kilgore, Mayur Ramesh, Jelena Verkler, Pardeep Pabla, Andrew Clark, Katrina Williams, Dee Dee Wang, Beverley Duthie, Samia Arshad, Alandra White, Anna Kern, Ashley Mattern, Bilqis Mosed, Dana Parke, Doreen Dankerlui, Dragana Spasevska, Hanah Woods, Helina Misikir, Howard Klausner, Janay Scott, Jessica Heinonen, John Zervos, Joseph Miller, Kate Zenlea, Kristin Eis, Marissa Vasquez, Maurice Slaughter, Meaghan Flynn, Michael Garcia, Michelle Sankah, Nina Paeilli, Philip Benson, Robert Devore, Stevanya Baho, Tony Eljallad, Tyler Prentiss, Yaman Ahmed, Sharon Mathys, Linda Kaljee, Jeffrey Van Laere, Claudia Hanni, Hassan Zafar, Mona Desai, Gina Maki, Mary Perri, Dora Vager, Shannon Thomas, Autumn Robinson, Isis Hamilton, Sonia Eliya, Jehan Jazrawi, Biljana Popovic, Sharon Zahul, Joshua Ruzzin, John Laguio, Ali Mathena, Bobby Cook Jr., Marlene Hesler, Rochelle Fleming, Terria Minniefield, John Simons, Sherese Henderson, Ashley Hopkins, Rebecca McFarlane, Raeshell Carson, Jonathan Williams, Katherine Reyes, Erica Herc, Indira Brar, Mayur Ramesh, John McKinnon, Lacquis Duncan, Tim Asmar, Margaret Beyer, Kaleem Chaudhry, Madison Lee, Jo-Ann Rammal, Karthik Sridasyam, Siddesh Veer, Angelique Buluran, Kimberlyn Lott, Jeremiah Rooker, Alayna Wilder, Kathleen Wilson, Allison Weinmann, Hassan Mourtada	Henry Ford Health System	Detroit, MI

United States Government (USG)/Coronavirus Prevention Network (CoVPN) Biostatistics Team

(PubMed listed, and ordered alphabetically by institution affiliation)

Affiliation	Team Members
Biomedical Advanced Research and Development Authority (BARDA), Washington, DC	Di Lu, James Zhou
Department of Biostatistics and Bioinformatics, Rollins School of Public Health, Emory University	David Benkeser
Vaccine and Infectious Disease Division, Fred Hutchinson Cancer Research Center, Seattle, WA	Jessica Andriesen, Bhavesh Borate, Lindsay N. Carpp, Andrew Fiore-Gartland, Youyi Fong*, Peter B. Gilbert*, Ying Huang*, Yunda Huang, Ellis Hughes, Ollivier Hyrien, Holly E. Janes*, Michal Juraska, Yiwen Lu, April K. Randhawa, Brian Simpkins, Brian D. Williamson, Lars W.P. van der Laan, Chenchen Yu
Biostatistics Research Branch, NIAID, NIH, Bethesda, MD	Michael P. Fay, Dean Follmann, Martha Nason
Division of Biostatistics, School of Public Health, University of California, Berkeley, CA	Nima S. Hejazi
Department of Biostatistics, University of Washington, Seattle, WA	Marco Carone, Kendrick Li, Wenbo Zhang
Department of Statistics, University of Washington, Seattle, WA	Alex Luedtke
Department of Population Health Sciences, Weill Cornell Medical College, New York, New York	Iván Díaz

*YF, PBG, YH, and HEJ are also affiliated with the Department of Biostatistics, University of Washington, Seattle, WA. PBG is also affiliated with the Public Health Sciences Division, Fred Hutchinson Cancer Research Center, Seattle, WA.

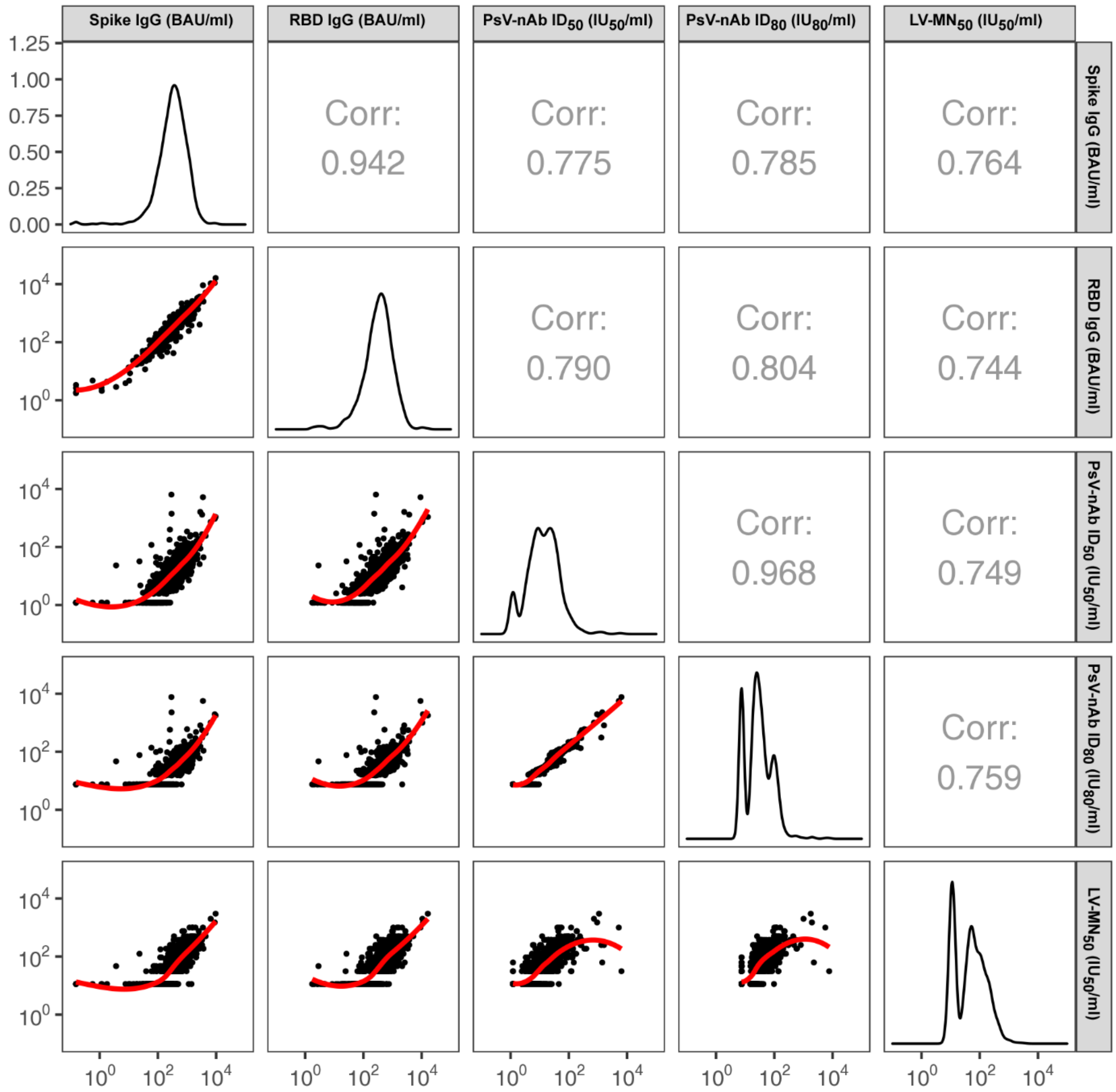

Fig. S1. In baseline SARS-CoV-2 negative per-protocol vaccine recipients in the immunogenicity subcohort, D29 LV-MN$_{50}$ titers are highly correlated with D29 spike IgG concentrations, D29 RBD IgG concentrations, D29 PsV-nAb ID$_{50}$ titers, and D29 PsV-nAb ID$_{80}$ titers.

Corr indicates baseline variable-adjusted Spearman rank correlation. ID$_{50}$, 50% inhibitory dilution; ID$_{80}$, 80% inhibitory dilution; LV, live virus; MN$_{50}$, 50% microneutralization dilution; nAb, neutralizing antibody; PsV, pseudovirus. Correlations of spike IgG, receptor binding domain (RBD) IgG, PsV-nAb ID$_{50}$, and PsV-nAb ID$_{80}$ were reported previously [fig. S5 in (*10*)] and are included here for comparison with LV-MN$_{50}$. Serological assay readouts are expressed in values relative to the World Health Organization (WHO) International Standard for anti-SARS-CoV-2 immunoglobulin (*27*): binding antibody readouts were converted to Binding Antibody Units per ml (BAU/ml) and pseudovirus neutralizing antibody titers as well as microneutralization assay readouts were calibrated to International Units/ml (IU$_{50}$/ml or IU$_{80}$/ml).

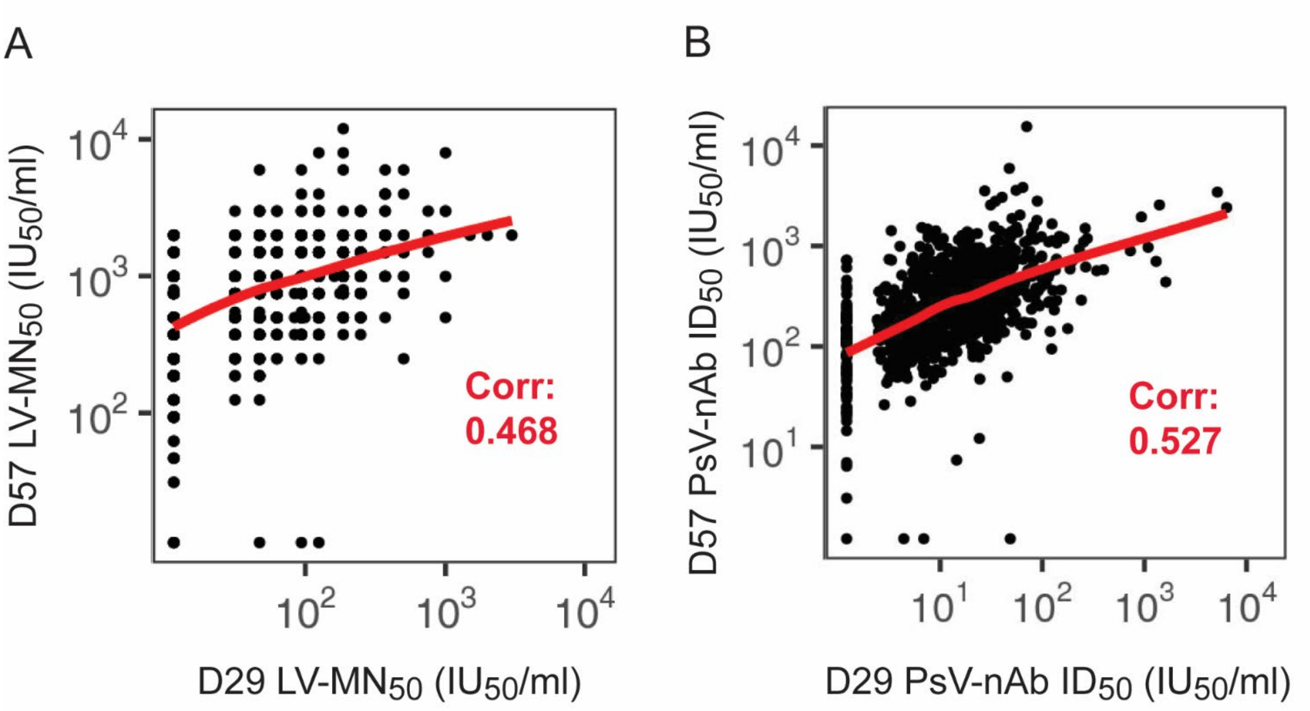

Fig. S2. D29 LV-MN$_{50}$ (PsV-nAb ID$_{50}$) titers correlate moderately with D57 LV-MN$_{50}$ (PsV-nAb ID$_{50}$) titers in baseline SARS-CoV-2 negative per-protocol vaccine recipients in the immunogenicity subcohort.

(A) LV-MN$_{50}$ and **(B)** PsV-nAb ID$_{50}$ values are presented. Corr indicates baseline variable-adjusted Spearman rank correlation. **(B)** was published previously [fig. S4C in (*10*)] and is included here for comparison. Serological assay readouts are expressed in values relative to the WHO International Standard for anti-SARS-CoV-2 immunoglobulin (*28*): binding antibody readouts were converted to BAU/ml and pseudovirus neutralizing antibody titers as well as microneutralization assay readouts were calibrated to IU$_{50}$/ml or IU$_{80}$/ml. ID$_{50}$, 50% inhibitory dilution; IU, international units; LV, live virus; MN$_{50}$, 50% microneutralization dilution; nAb, neutralizing antibody; PsV, pseudovirus.

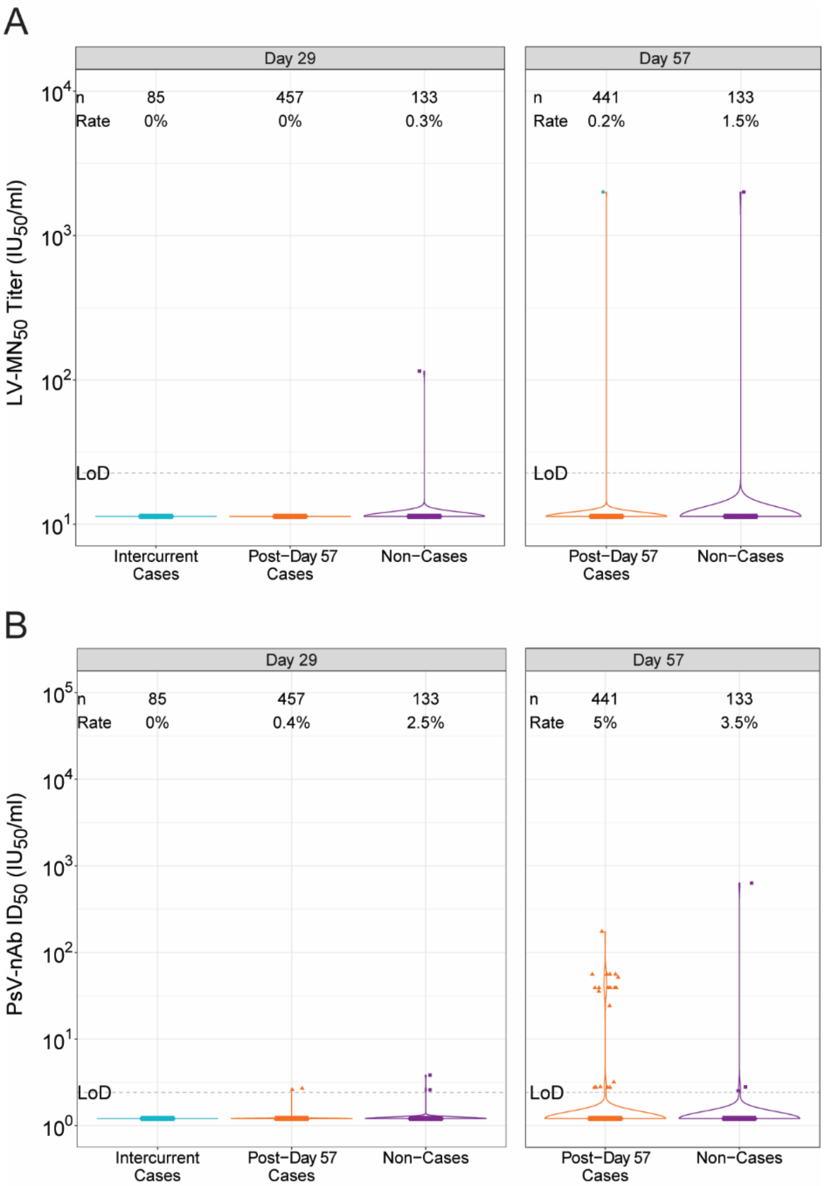

Fig. S3. D29 and D57 LV-MN50 and PsV-nAb ID50 titers and response rates are zero or near-zero in baseline SARS-CoV-2 negative per-protocol placebo recipients.

(A) LV-MN_{50} and **(B)** PsV-nAb ID_{50} values are presented by COVID-19 outcome status in placebo recipients. Data points are from baseline SARS-CoV-2 negative per-protocol placebo recipients in the D29 marker or D57 marker case-cohort set. Post D57 cases are COVID-19 endpoints starting 7 days post D57 through the end of blinded follow-up (last COVID-19 endpoint 126 days post dose two); Intercurrent cases are COVID-19 endpoints starting 7 days post D29 through 6 days post D57. The violin plots contain interior box plots with upper and lower horizontal edges showing the 25th and 75th percentiles of antibody concentrations and the middle showing line the 50th percentile. Vertical bars show the distance from the 25th (or 75th) percentile and the minimum (or maximum) within the 25th (or 75th) percentile minus (or plus) 1.5 times the interquartile range. Each side shows a rotated probability density (estimated by a kernel density estimator with a default Gaussian kernel) of the data. Positive response for MN_{50} was defined by value > limit of detection (LoD; 22.66 IU_{50}/ml). Positive/detectable response for ID_{50}

was defined by value > LoD (2.42 IU_{50}/ml). Positive response rates are computed with Inverse Probability Sampling (IPS) weighting. The pseudovirus-nAb ID_{50} values were previously published [fig. S8C in (*10*)] and are included here for comparison. The minor differences in PsV-nAb ID_{50} response rates between those in (*10*) and those here are due to the fact that in (*10*) the lower limit of quantitation (LLOQ) was used to define positive response rate for the pseudovirus neutralization assay (instead of LoD as incorrectly stated), whereas LoD was used to define positive response rate for the pseudovirus neutralization assay in the present work. Serological assay readouts are expressed in values relative to the WHO International Standard for anti-SARS-CoV-2 immunoglobulin (*27*): pseudovirus neutralizing antibody titers and microneutralization assay readouts were calibrated to IU_{50}/ml. LV, live virus; PsV, pseudovirus; nAb, neutralizing antibody; ID_{50}, 50% inhibitory dilution; MN_{50}, 50% microneutralization dilution.

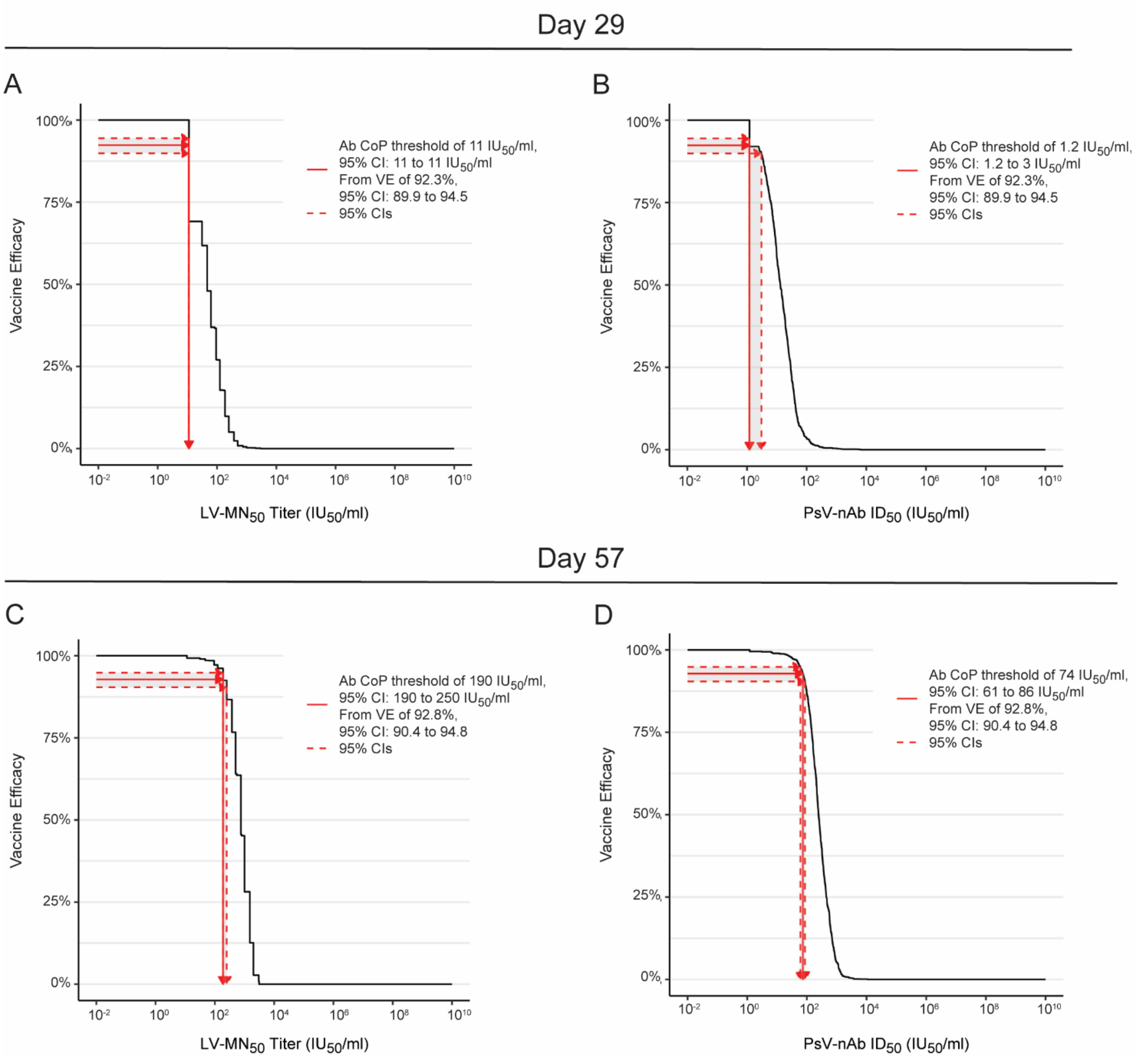

Fig. S4. By applying the Siber method (*35*), a threshold of perfect vs. no protection for each antibody marker is estimated.

Shown are inverse probability sampling (IPS)-weighted empirical reverse cumulative distribution function curves for **(A)** D29 LV-MN_{50}, **(B)** D29 PsV-nAb ID_{50}, **(C)** D57 LV-MN_{50}, and **(D)** D29 PsV-nAb ID_{50}. **(B)** and **(D)** were previously published [fig. S12C and fig. 11C, respectively, in (*10*)] and are included here for comparison. Serological assay readouts are expressed in values relative to the WHO International Standard for anti-SARS-CoV-2 immunoglobulin (*27*): PsV nAb titers and MN assay readouts were calibrated to IU_{50}/ml. Ab, antibody; CoP, correlate of protection; CI, confidence interval; ID_{50}, 50% inhibitory dilution; LV, live virus; MN_{50}, nAb, neutralizing antibody; 50% microneutralization dilution; PsV, pseudovirus; VE, vaccine efficacy.

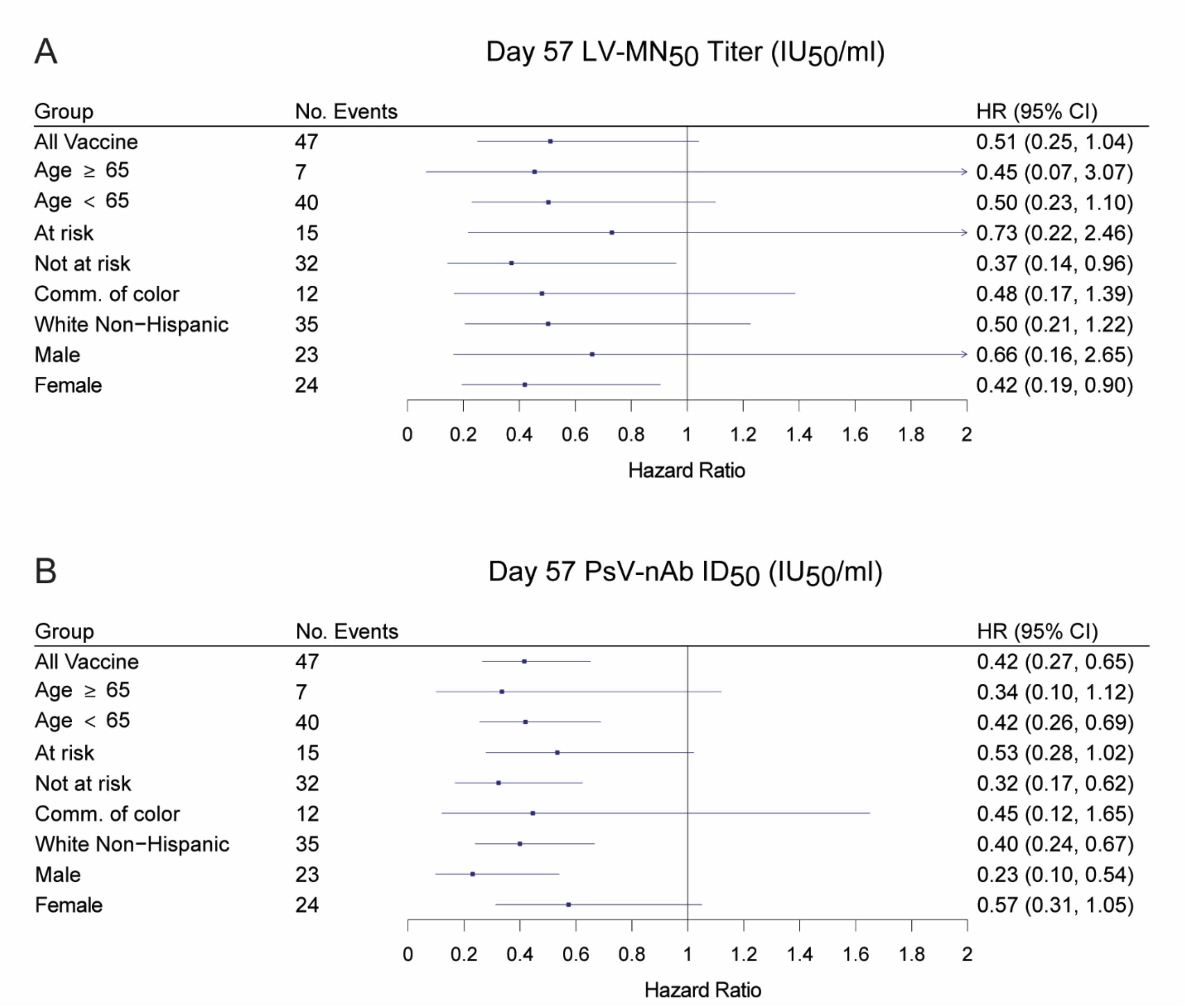

Fig. S5. Hazard ratios (HRs) of COVID-19 in prespecified vaccine recipient subgroups per 10-fold increase in each D57 neutralizing antibody marker are all less than 1, but with most 95% confidence intervals for LV-MN_{50} titer overlapping one.

(A) LV-MN_{50} titer and **(B)** PsV-nAb ID_{50} titer are shown for baseline SARS-CoV-2 negative per-protocol vaccine recipients overall and for individual subgroups. Community (Comm.) of color is the complement of White Non-Hispanic participants (different from Minority because missing values are included in Community of color). **(B)** was published previously as Fig. 3C in (*10*) and is included here for comparison. Baseline covariates adjusted for baseline risk score, at risk status, and community of color status. Serological assay readouts assessed as immune correlates were first expressed in values relative to the WHO International Standard for anti-SARS-CoV-2 immunoglobulin (*27*): pseudovirus neutralizing antibody titers and microneutralization assay readouts were calibrated to IU_{50}/ml. CI, confidence interval; ID_{50}, 50% inhibitory dilution; IU, international units; LV, live virus; MN_{50}, 50% microneutralization dilution; nAb, neutralizing antibody; PsV, pseudovirus.

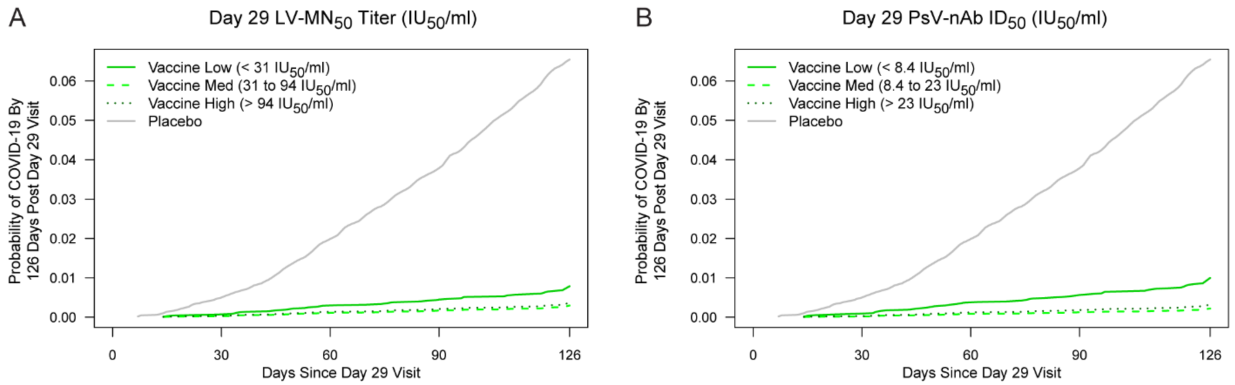

A — No. At-Risk*

	0	30	60	90	126
Low:	5388	5383	5332	4885	1994
Med:	4941	4940	4898	4429	1966
High:	3812	3812	3798	3509	1568
Plac:	13972	13856	13250	11117	3963

A — Cumulative No. of COVID-19 Endpoints**

	0	30	60	90	126
Low:	0	5	16	22	31
Med:	0	1	6	11	12
High:	0	0	4	6	12
Plac:	0	71	278	507	734

B — No. At-Risk*

	0	30	60	90	126
Low:	4688	4684	4635	4371	1687
Med:	4778	4778	4737	4185	1917
High:	4675	4673	4656	4266	1925
Plac:	13972	13856	13250	11117	3963

B — Cumulative No. of COVID-19 Endpoints**

	0	30	60	90	126
Low:	0	4	18	25	33
Med:	0	0	4	5	8
High:	0	2	4	8	13
Plac:	0	71	278	507	734

*No. At-Risk = estimated number in the population for analysis: baseline negative per-protocol vaccine recipients not experiencing the COVID-19 endpoint through 6 days post Day 29 visit.
**Cumulative No. of COVID-19 Endpoints = estimated cumulative number of this cohort with a COVID-19 endpoint.

C

COVE Immunologic Marker	Tertile	No. cases / No. at-risk	Attack rate	Haz. Ratio Pt. Est.	Haz. Ratio 95% CI	P-value (2-sided)	Overall P-value	FDR-adjusted P-value	FWER-adjusted P-value
Live Virus-MN$_{50}$ (IU$_{50}$/ml)	Low	31/5,388	0.0058	1	N/A	N/A	0.025	0.026	0.021
	Medium	12/4,941	0.0024	0.37	(0.17,0.82)	0.014			
	High	12/3,812	0.0031	0.46	(0.21,1.01)	0.053			
Pseudovirus-nAb ID$_{50}$ (IU$_{50}$/ml)	Low	33/4,688	0.0070	1	N/A	N/A	<0.001	0.001	0.001
	Medium	8/4,778	0.0017	0.22	(0.09,0.53)	<0.001			
	High	13/4,675	0.0028	0.32	(0.15,0.69)	0.003			
Placebo		751/13,972	0.0538						

Fig. S6. Analyses of COVID-19 risk by Low, Medium, and High tertiles show evidence for D29 LV-MN$_{50}$ and D29 PsV-nAb ID$_{50}$ as correlates of risk.

The plots and table show cumulative incidence of COVID-19 by Low, Medium, and High tertiles of **(A)** D29 LV-MN$_{50}$ titer or **(B)** D29 PsV-nAb ID$_{50}$ titer in baseline SARS-CoV-2–negative per-protocol vaccine recipients. **(C)** Estimated hazard ratios of COVID-19 for Medium vs. Low and for High vs. Low tertile of D29 antibody marker titer. The overall p-value is from a generalized Wald test for whether the COVID-19 hazard differed across Low, Medium, and High subgroups. The PsV-nAb ID$_{50}$ results in **(B)** and **(C)** were previously published [fig. S18B and S18C, respectively, in (*10*)] and are included here for comparison. Baseline covariates adjusted for were baseline risk score, at risk status, and community of color status. Serological assay readouts assessed as immune correlates were first expressed in values relative to the WHO International Standard for anti-SARS-CoV-2 immunoglobulin (*27*): pseudovirus neutralizing antibody titers and microneutralization assay readouts were calibrated to IU$_{50}$/ml. Maximum failure event time 126 days post D29 visit. Tertile values are given in the legends in **(A)** and **(B)**. No. at-risk = estimated number in the population for analysis: baseline negative per-protocol vaccine recipients not experiencing the COVID-19 endpoint through 6 days post D29 visit; No. cases = estimated number of this cohort with an observed COVID-19 endpoint. The total count

(55) across all tertiles for each marker differs from 46 (Fig. 2, Table 1), because the 55 includes all vaccine breakthrough cases including the 9 without D1, D29, D57 antibody marker data. FDR (false discovery rate)-adjusted p-values and FWER (family-wise error rate)-adjusted p-values are computed over the set of p-values both for quantitative markers and categorical markers (Low, Medium, High) using the Westfall and Young permutation method (10,000 replicates). CI, confidence interval; ID_{50}, 50% inhibitory dilution; IU, international units; LV, live virus; MN_{50}, 50% microneutralization dilution; nAb, neutralizing antibody; PsV, pseudovirus; Pt. Est., point estimate.

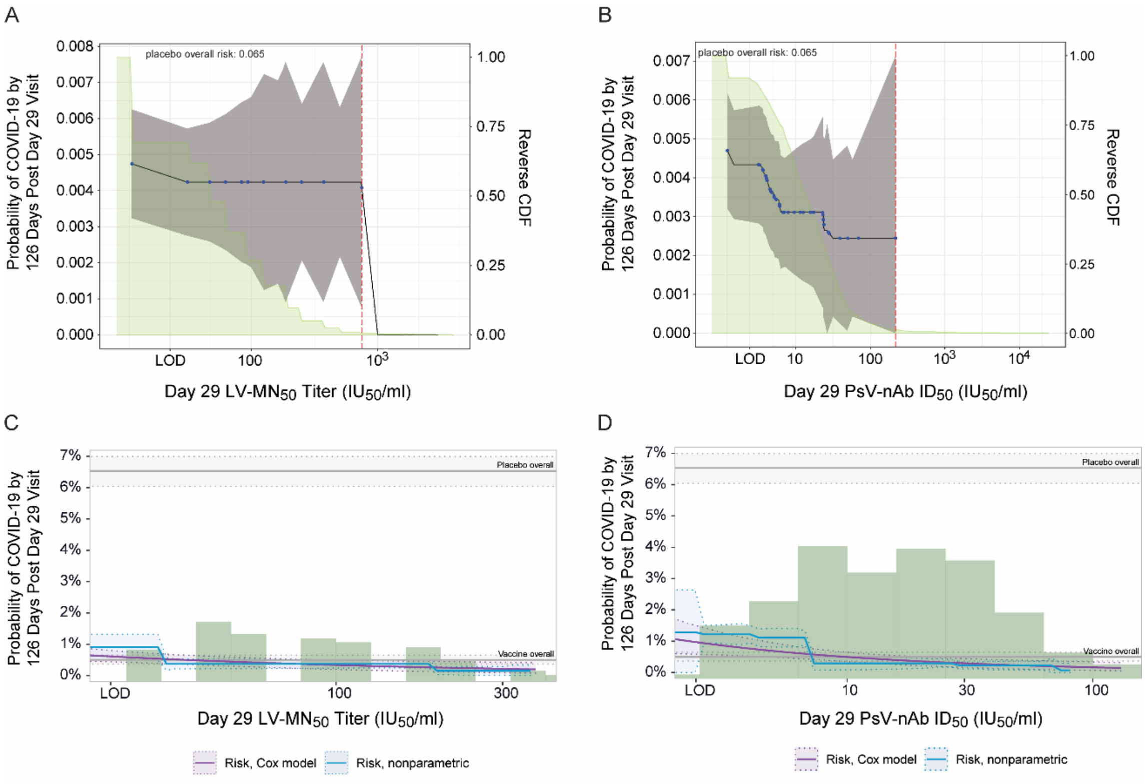

Fig. S7. Further correlate of risk analyses show weaker evidence for D29 LV-MN$_{50}$ compared to D29 PsV-nAb ID$_{50}$ as a correlate of risk.

(A and B) Cumulative incidence of COVID-19 by 126 days after D29 by vaccinated baseline SARS-CoV-2–negative per-protocol subgroups defined by D29 **(A)** LV-MN$_{50}$ or **(B)** ID$_{50}$ titer above a threshold, with reverse cumulative distribution function (CDF) of D29 marker titer overlaid in green. The blue dots correspond to marker values where an event is observed, linearly interpolated by solid black lines. The gray shaded area is pointwise 95% CIs. The upper boundary of the green shaded area is the estimate of the reverse cumulative distribution function (CDF) of the D29 marker value in baseline SARS-CoV-2–negative per-protocol vaccine recipients. The vertical red dashed line is the marker threshold above which no post–D29 COVID-19 endpoints occurred. **(C and D)** Cumulative incidence of COVID-19 by 126 days post D29 is shown by **(C)** D29 LV-MN$_{50}$ titer or **(D)** D29 PsV-nAb titer, estimated using (solid purple line) a Cox model or (solid blue line) a nonparametric model. The purple dotted lines indicate bootstrap pointwise 95% CIs and the blue dotted lines indicate influence-function-based Wald-based 95% CIs. The upper and lower horizontal gray lines are the overall cumulative incidence of COVID-19 from 7 to 126 days post D29 in placebo and vaccine recipients, respectively. **(B)** and **(D)** were previously published [fig. S22A and S22B, respectively, of (*10*) and are shown here for comparison]. The green histograms indicate the frequency distribution of D29 marker among baseline SARS-CoV-2 negative per-protocol vaccine recipients. All analyses adjusted for the following baseline covariates: baseline risk score, at risk status, and community of color status. Serological assay readouts assessed as immune correlates were first expressed in values relative to the WHO International Standard for anti-SARS-CoV-2 immunoglobulin (*27*): pseudovirus neutralizing antibody titers and microneutralization assay readouts were calibrated to IU$_{50}$/ml. CI, confidence interval; ID$_{50}$, 50%

inhibitory dilution; IU, international units; LV, live virus; MN_{50}, 50% microneutralization dilution; nAb, neutralizing antibody; PsV, pseudovirus.

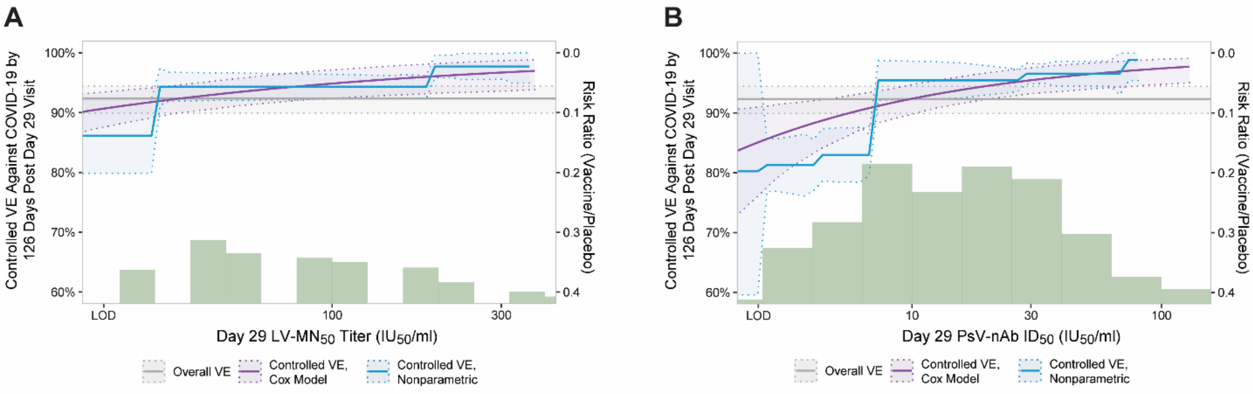

C

	D29 LV-MN50		D57 LV-MN50	
Controlled VE Pt. Est.	87.7%	97.0%	87.6%	95.5%
Marker Percentile	2.5th	97.5th	2.5th	97.5th
Percentile Value (IU50/ml)	11.3	375	93.7	2998

Fig. S8. Correlate of protection analyses show that vaccine efficacy against COVID-19 increases with D29 LV-MN50 titer and more strongly with D29 PsV-nAb ID50 titer.

(A and B) Vaccine efficacy (solid purple line) by D29 **(A)** LV-MN50 titer or **(B)** PsV-nAb ID50 titer was estimated using a Cox proportional hazards implementation of (*30*). The dashed gray lines indicate bootstrap point-wise 95% CIs. Vaccine efficacy (solid blue line) by D29 **(A)** PsV-nAb ID50 titer or **(B)** LV-MN50 titer was estimated using a nonparametric implementation of (*30*). The blue shaded area represents the 95% CIs. The green histograms indicate the frequency distribution of D29 marker among baseline SARS-CoV-2 negative per-protocol vaccine recipients. The horizontal gray line is the overall vaccine efficacy from 7 to 126 days post D29, with the dotted gray lines indicating the 95% CIs. Baseline covariates adjusted for were baseline risk score, at risk status, and community of color status. Serological assay readouts assessed as immune correlates were first expressed in values relative to the WHO International Standard for anti-SARS-CoV-2 immunoglobulin (*27*): pseudovirus neutralizing antibody titers and microneutralization assay readouts were calibrated to IU50/ml. **(C)** Comparison of controlled VE point estimates at the 2.5th and 97.5th marker percentiles for D29 LV-MN50 (fig. S8A) and D57 LV-MN50 (Fig. 2F). CI, confidence interval; ID50, 50% inhibitory dilution; IU, international units; LV, live virus; MN50, 50% microneutralization dilution; nAb, neutralizing antibody; PsV, pseudovirus.

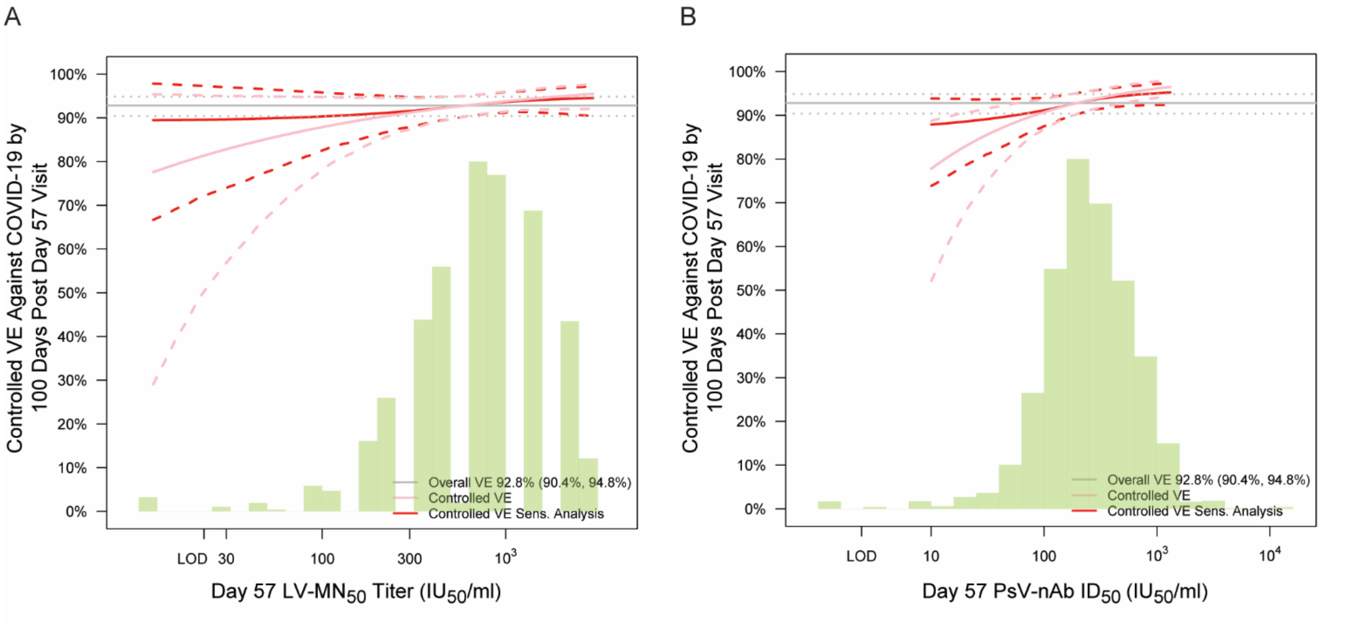

Fig. S9. Estimated vaccine efficacy against COVID-19 still increases, but to a lesser extent, with increasing D57 LV-MN$_{50}$ titer and D57 PsV-nAb ID$_{50}$ titer in a sensitivity analysis.

Vaccine efficacy is shown by D57 **(A)** LV-MN$_{50}$ and **(B)** PsV-nAb ID$_{50}$ titer. Vaccine efficacy estimates were obtained using the method of Gilbert, Fong, and Carone (*30*). The pink solid line represents point estimates assuming no unmeasured confounding; the dashed lines are bootstrap point-wise 95% CIs. The red solid line represents point estimates assuming unmeasured confounding in a sensitivity analysis (dashed lines are bootstrap point-wise 95% CIs). See supplementary text S2 in (*16*) and the Statistical Analysis Plan (Section 12.1.2) for details of the sensitivity analysis. The horizontal gray line is the overall vaccine efficacy from 7 to 100 days post D57, with the dotted gray lines indicating the 95% CIs (this number 92.8% differs from the 94.1% reported in (*16*), which was based on counting COVID-19 endpoints starting 14 days post D29). The green histogram is an estimate of the frequency distribution of D57 marker among baseline SARS-CoV-2 negative per-protocol vaccine recipients. Serological assay readouts assessed as immune correlates were first expressed in values relative to the WHO International Standard for anti-SARS-CoV-2 immunoglobulin (*27*): pseudovirus neutralizing antibody titers and microneutralization assay readouts were calibrated to IU$_{50}$/ml. CI, confidence interval; ID$_{50}$, 50% inhibitory dilution; IU, international units; LV, live virus; MN$_{50}$, 50% microneutralization dilution; nAb, neutralizing antibody; PsV, pseudovirus.

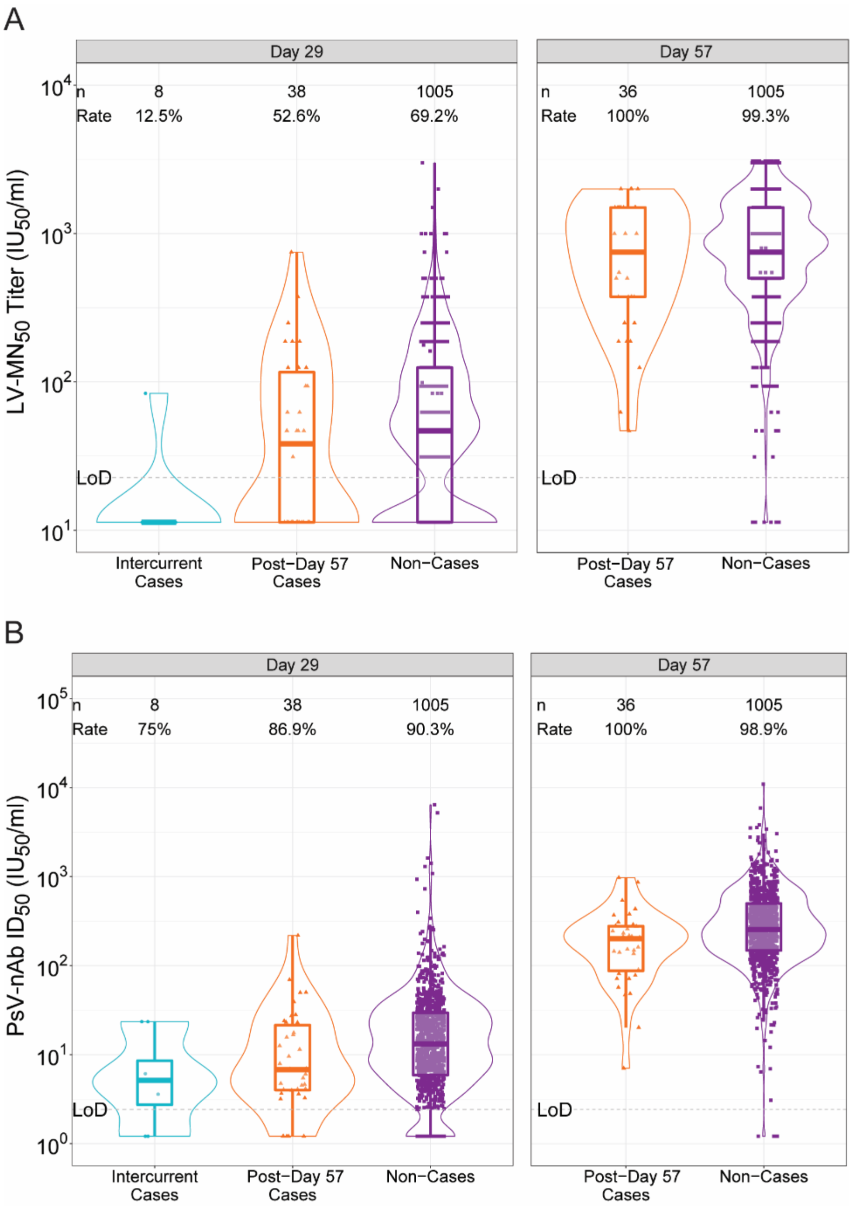

Fig. S10. Response rates and neutralizing antibody titers are higher at D57 than at D29, for both LV-MN$_{50}$ and PsV-nAb ID$_{50}$.

(A) LV-MN$_{50}$ and **(B)** PsV-nAb ID$_{50}$ titers were stratified by COVID-19 outcome status in vaccine recipients. Data points are from baseline negative per-protocol vaccine recipients in the D29 marker or D57 marker case-cohort set. Post D57 cases are COVID-19 endpoints starting 7 days post D57 through the end of blinded follow-up (last COVID-19 endpoint 126 days post dose 2); Intercurrent cases are COVID-19 endpoints starting 7 days post D29 through 6 days post D57. The violin plots contain interior box plots with upper and lower horizontal edges indicating the 25th and 75th percentiles of antibody concentration and middle line the 50th percentile; vertical bars represent the distance from the 25th (or 75th) percentile and the minimum (or maximum) within the 25th (or 75th) percentile minus (or plus) 1.5 times the interquartile range. Each side shows a rotated probability density (estimated by a kernel density estimator with a default Gaussian kernel) of the data. Positive response rates were computed with inverse probability of

sampling weighting. The PsV-nAb ID_{50} results in **(B)** were previously published [Fig. 1B in (*10*)] and are included here for comparison. Positive response for MN_{50} was defined by value > LoD (22.66 IU_{50}/ml). Positive/detectable response for ID_{50} was defined by value > LoD (2.42 IU_{50}/ml). The minor differences in pseudovirus-nAb ID_{50} response rates between those in (*10*) and those here are due to the fact that in (*10*) the lower limit of quantitation (LLOQ) was used to define positive response rate for the pseudovirus neutralization assay (instead of LoD as incorrectly stated), whereas LoD was used to define positive response rate for the pseudovirus neutralization assay in this work. Serological assay readouts are expressed in values relative to the WHO International Standard for anti-SARS-CoV-2 immunoglobulin (*27*): pseudovirus neutralizing antibody titers and microneutralization assay readouts were calibrated to IU_{50}/ml. ID_{50}, 50% inhibitory dilution; IU, international units; LV, live virus; MN_{50}, 50% microneutralization dilution; nAb, neutralizing antibody; PsV, pseudovirus.

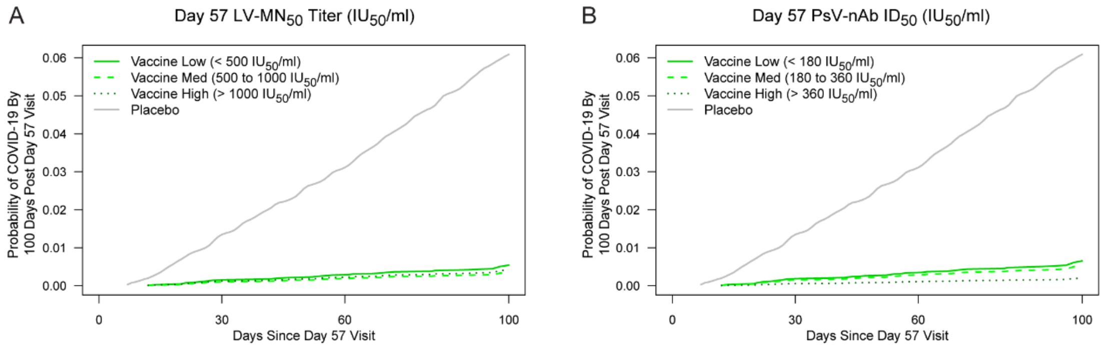

A

No. At-Risk*

	0	30	60	100
Low:	5053	5023	4608	1703
Med:	5056	4987	4559	1649
High:	3955	3939	3704	1572
Plac:	13758	13218	11165	3364

Cumulative No. of COVID-19 Endpoints**

	0	30	60	100
Low:	0	8	13	20
Med:	0	4	8	13
High:	0	5	9	14
Plac:	0	187	409	646

B

No. At-Risk*

	0	30	60	100
Low:	4727	4705	4384	1391
Med:	4681	4635	4260	1669
High:	4656	4609	4228	1865
Plac:	13758	13218	11165	3364

Cumulative No. of COVID-19 Endpoints**

	0	30	60	100
Low:	0	10	13	21
Med:	0	4	10	18
High:	0	3	7	8
Plac:	0	187	409	646

*No. At-Risk = estimated number in the population for analysis: baseline negative per-protocol vaccine recipients not experiencing the COVID-19 endpoint through 6 days post Day 57 visit.
**Cumulative No. of COVID-19 Endpoints = estimated cumulative number of this cohort with a COVID-19 endpoint.

C

COVE Immunologic Marker	Tertile	No. cases / No. at-risk	Attack rate	Haz. Ratio Pt. Est.	Haz. Ratio 95% CI	P-value (2-sided)	Overall P-value	FDR-adjusted P-value	FWER-adjusted P-value
Live Virus-MN50 (IU50 /ml)	Low	20/5,053	0.0040	1	N/A	N/A	0.579	0.587	0.571
	Medium	13/5,056	0.0026	0.66	(0.30,1.46)	0.305			
	High	14/3,955	0.0035	0.78	(0.34,1.77)	0.551			
Pseudovirus-nAb ID50 (IU50/ml)	Low	21/4,727	0.0044	1	N/A	N/A	0.052	0.067	0.108
	Medium	18/4,681	0.0038	0.82	(0.39, 1.72)	0.599			
	High	8/4,656	0.0017	0.31	(0.12, 0.80)	0.016			
Placebo		659/13,758	0.0479						

Fig. S11. Analyses of COVID-19 risk by Low, Medium, and High tertiles show no evidence for D57 LV-MN_{50} or D57 PsV-nAb ID_{50} as a correlate of risk.

The plots and table show cumulative incidence of COVID-19 by Low, Medium, and High tertiles of **(A)** D57 LV-MN_{50} titer or **(B)** D57 PsV-nAb ID_{50} titer in baseline SARS-CoV-2–negative per-protocol vaccine recipients. **(C)** Estimated hazard ratios of COVID-19 for Medium vs. Low and for High vs. Low tertile of D57 antibody marker titer. The overall p-value is from a generalized Wald test of whether the hazard rate of COVID-19 differed across the Low, Medium, and High subgroups. The PsV-nAb ID_{50} results in **(B)** and **(C)** were previously published [Fig. 2B and 2C, respectively, in (*10*)] and are included here for comparison. Baseline covariates adjusted for were baseline risk score, at risk status, and community of color status. Serological assay readouts assessed as immune correlates were first expressed in values relative to the WHO International Standard for anti-SARS-CoV-2 immunoglobulin (*27*): pseudovirus neutralizing antibody titers and microneutralization assay readouts were calibrated to International Units/ml (IU_{50}/ml). Maximum failure event time 100 days post D57 visit. Tertile values are given in the legends in **(A)** and **(B)**. No. at-risk = estimated number in the population for analysis: baseline negative per-protocol vaccine recipients not experiencing the COVID-19 endpoint through 6 days post D57 visit; No. cases = estimated number of this cohort with an observed COVID-19 endpoint. The total count (47) across all tertiles for each marker differs from 36 (Fig. 2, Table 1), because the 47 includes all vaccine breakthrough cases including the 9 without D1, D29, D57 antibody marker data. FDR (false discovery rate)-adjusted p-values and

FWER (family-wise error rate)-adjusted p-values are computed over the set of p-values both for quantitative markers and categorical markers (Low, Medium, High) using the Westfall and Young permutation method (10,000 replicates). CI, confidence interval; ID_{50}, 50% inhibitory dilution; IU, international units; FDR, false discovery rate; FWER, family-wise error rate; LV, live virus; MN_{50}, 50% microneutralization dilution; nAb, neutralizing antibody; PsV, pseudovirus; Pt. Est., point estimate.

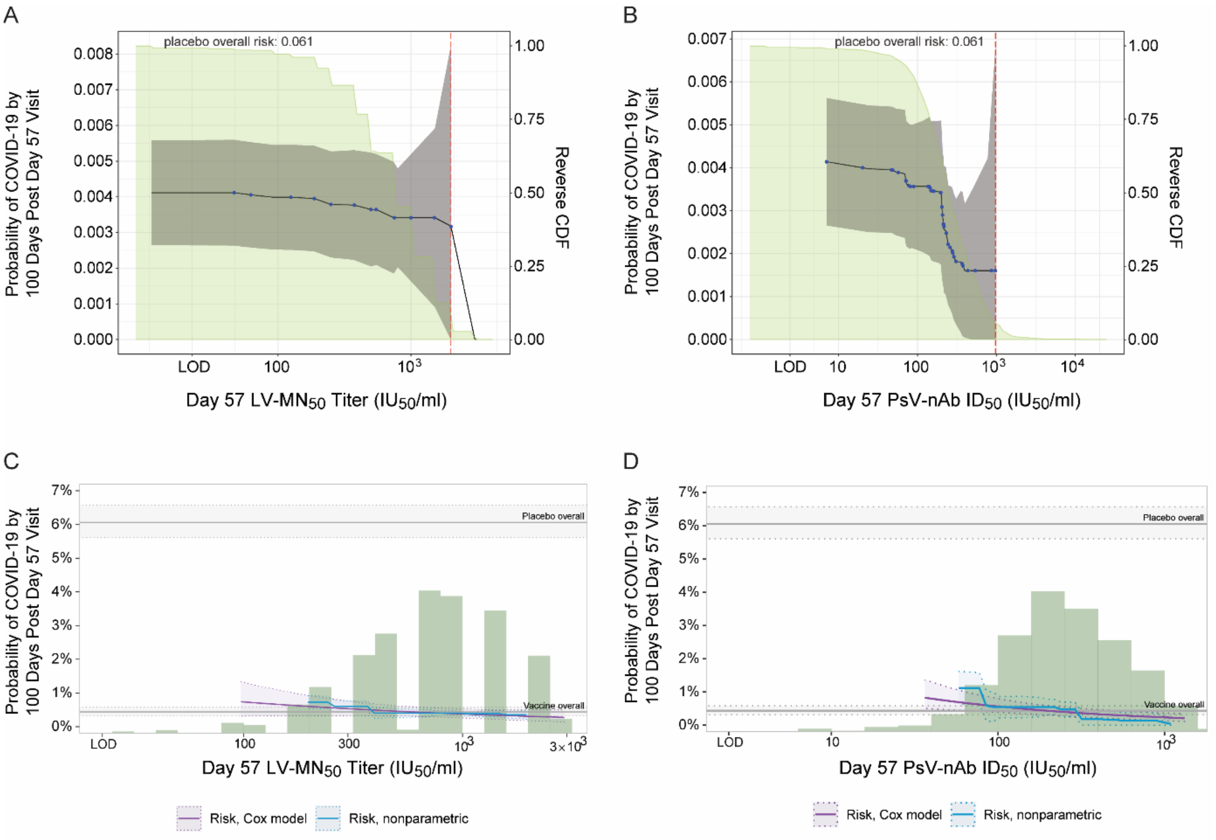

Fig. S12. Correlate of risk analyses show evidence for both D57 LV-MN$_{50}$ and D57 PsV-nAb ID$_{50}$ as correlates of risk, with stronger evidence for D57 PsV-nAb ID$_{50}$.

(A and B) Cumulative incidence of COVID-19 by 100 days after D57 in vaccinated baseline SARS-CoV-2–negative per-protocol subgroups defined by D57 **(A)** LV-MN$_{50}$ or **(B)** PsV-nAb ID$_{50}$ titer above a threshold, with reverse cumulative distribution function (CDF) of D57 marker titer overlaid in green. The blue dots correspond to marker values where an event is observed, linearly interpolated by solid black lines. The gray shaded area is pointwise 95% CIs. The upper boundary of the green shaded area is the estimate of the reverse cumulative distribution function (CDF) of the D57 marker value in baseline SARS-CoV-2–negative per-protocol vaccine recipients. The vertical red dashed line is the marker threshold above which no post–D57 COVID-19 endpoints occurred. **(C and D)** Cumulative incidence of COVID-19 by 100 days post D57 is shown by **(C)** D57 LV-MN$_{50}$ titer or **(D)** D57 PsV-nAb titer, estimated using (solid purple line) a Cox model or (solid blue line) a nonparametric model. The purple dotted lines indicate bootstrap pointwise 95% CIs and the blue dotted lines indicate influence-function-based Wald-based 95% CIs. The upper and lower horizontal gray lines are the overall cumulative incidence of COVID-19 from 7 to 100 days post D57 in placebo and vaccine recipients, respectively. The dotted black lines indicate bootstrap pointwise 95% CIs. **(B)** and **(D)** were previously published [Fig. 4A and 4B, respectively, of (*10*) and are shown here for comparison]. The green histograms indicate the frequency distribution of D29 marker among baseline SARS-CoV-2 negative per-protocol vaccine recipients. All analyses adjusted for the following baseline covariates: baseline risk score, at risk status, and community of color status. Serological assay

readouts assessed as immune correlates were first expressed in values relative to the WHO International Standard for anti-SARS-CoV-2 immunoglobulin (*27*): pseudovirus neutralizing antibody titers and microneutralization assay readouts were calibrated to IU_{50}/ml. CI, confidence interval; ID_{50}, 50% inhibitory dilution; IU, international units; LV, live virus; MN_{50}, 50% microneutralization dilution; nAb, neutralizing antibody; PsV, pseudovirus.

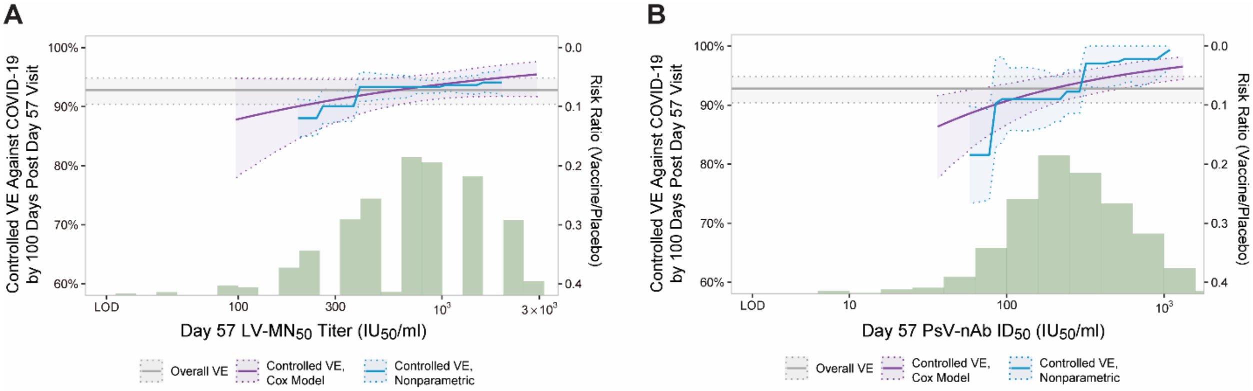

Fig. S13. Correlate of protection analyses of D57 neutralizing antibody concentrations show that vaccine efficacy against COVID-19 increases with D57 LV-MN$_{50}$ titer and more strongly with D57 PsV-nAb ID$_{50}$ titer.

(A and B) Vaccine efficacy (solid purple line) by D57 **(A)** LV-MN$_{50}$ titer or **(B)** PsV-nAb ID$_{50}$ titer was estimated using a Cox proportional hazards implementation of (*30*). The dashed gray lines indicate bootstrap point-wise 95% CIs. Vaccine efficacy (solid blue line) by D57 **(A)** live virus-MN$_{50}$ titer or **(B)** PsV-nAb ID$_{50}$ titer was estimated using a nonparametric implementation of (*30*). The blue shaded area represents the 95% CIs. The green histograms indicate the frequency distribution of D57 marker among baseline SARS-CoV-2 negative per-protocol vaccine recipients. The horizontal gray line is the overall vaccine efficacy from 7 to 100 days post D57, with the dotted gray lines indicating the 95% CIs. Baseline covariates adjusted for were baseline risk score, at risk status, and community of color status. Serological assay readouts assessed as immune correlates were first expressed in values relative to the WHO International Standard for anti-SARS-CoV-2 immunoglobulin (*27*): pseudovirus neutralizing antibody titers and microneutralization assay readouts were calibrated to IU$_{50}$/ml. CI, confidence interval; ID$_{50}$, 50% inhibitory dilution; IU, international units; LV, live virus; MN$_{50}$, 50% microneutralization dilution; nAb, neutralizing antibody; PsV, pseudovirus.

Table S1. Numbers of per-protocol baseline negative participants with antibody markers measured and included in each of the D29 and D57 correlates analyses, for each of the five immunoassays. Cases for D29 marker correlates analyses (Intercurrent cases + Post D57 cases) are baseline SARS-CoV-2 negative per-protocol vaccine recipients with the symptomatic infection COVID-19 primary endpoint diagnosed starting 7 days after D29 through the end of the blinded phase. Cases for D57 marker correlates analyses (Post D57 cases) are baseline SARS-CoV-2 negative per-protocol vaccine recipients with the symptomatic infection COVID-19 primary endpoint diagnosed starting 7 days after D57 through the end of the blinded phase. The last COVID-19 endpoint within the blinded phase occurred 100 days after D57.

	**D29 Correlates Analyses**		**D57 Correlates Analyses**	
**Antibody Marker**	**Cases**	**Non-Cases**	**Cases**	**Non-Cases**
Binding antibody to spike	46	1005	36	1005
Binding antibody to RBD	46	1005	36	1005
PsV-nAb ID_{50} titer	42	1005	32	1005
PsV-nAb ID_{80} titer	42	1005	32	1005
LV-MN_{50}	39	985	31	968

Table S2. Comparison of live virus neutralization MN_{50} titer and pseudovirus neutralization ID_{50} titer response rates and Geometric Mean Titers (GMTs) by COVID-19 outcome status. Analysis based on baseline negative per-protocol vaccine recipients in the D29 marker or D57 marker case-cohort sets. Median (interquartile range) days from dose one to D29 was 28 (28 to 30) and from D29 to D57 was 28 (28 to 30) are shown. [1]Cases for D29 marker correlates analyses (Intercurrent cases + Post D57 cases) are baseline SARS-CoV-2 negative per-protocol vaccine recipients with the symptomatic infection COVID-19 primary endpoint diagnosed starting 7 days after D29 through the end of the blinded phase. Cases for D57 marker correlates analyses (Post D57 cases) are baseline SARS-CoV-2 negative per-protocol vaccine recipients with the symptomatic infection COVID-19 primary endpoint diagnosed starting 7 days after D57 through the end of the blinded phase. The last COVID-19 endpoint within the blinded phase occurred 100 days after D57. [2]N for Cases for D29 marker analyses is the number of vaccine breakthrough cases with D1 and D29 antibody marker data included, and N for Cases for D57 marker analyses is the number of vaccine breakthrough cases with D1, D29, and D57 antibody data included. [3]N for Non-Cases in Immunogenicity Subcohort is the number of participants with D1, D29, and D57 antibody marker data included in both the D29 and D57 marker correlates analyses. [4]Serological assay readouts are expressed in values relative to the WHO International Standard for anti-SARS-CoV-2 immunoglobulin (*28*): pseudovirus neutralizing antibody titers and microneutralization assay readouts were calibrated to IU_{50}/ml. [5]Pseudovirus-nAb ID_{50} GMTs and ratios were previously published *(16)* and are included here for comparison. The pseudovirus-nAb ID_{50} response rates in this table differ from those in Table 1 in (*10*) because here positive response for ID_{50} was defined by value > LoD (2.42 IU_{50}/ml), whereas in *(16)* positive response for ID_{50} was defined by value > LLOQ (4.477 IU_{50}/ml).

		COVID-19 Cases[1]			Non-Cases in Immunogenicity Subcohort			Comparison	
**Visit for Marker**	**Marker**	**N[2]**	**Response Rate (95% CI)**	**GMT (95% CI)**	**N[3]**	**Response Rate (95% CI)**	**GMT (95% CI)**	**Response Rate Difference (95% CI)**	**Ratio of GM (Cases/Non-Cases) (95% CI)**
D29	LV-MN_{50} (IU_{50}/ml)[4]	46	45.7% (31.6%, 60.4%)	31.4 (22.0, 45.0)	1005	69.2% (65.8%, 72.4%)	48.4 (44.6, 52.6)	-24% (-38, -8.4%)	0.65 (0.45, 0.94)
D29	PsV-nAb ID_{50} (IU_{50}/ml)[5]	46	84.8% (70.9%, 92.7%)	7.6 (5.4, 10.8)	1005	92.0% (89.8%, 93.7%)	13.0 (11.9, 14.1)	-7.2% (-21, 1%)	0.59 (0.41, 0.84)
D57	LV-MN_{50} (IU_{50}/ml)	36	100.0% (100.0%, 100.0%)	594 (433, 816)	1005	99.3% (98.3%, 99.7%)	718 (676, 763)	1% (0, 2%)	0.83 (0.60, 1.14)
D57	PsV-nAb ID_{50} (IU_{50}/ml)	36	100.0% (100.0%, 100.0%)	160 (117, 220)	1005	99.5% (98.6%, 99.8%)	247 (231, 264)	1% (0, 1%)	0.65 (0.47, 0.90)

Table S3. Ranking of antibody marker performance in each of three categories of immune correlate-quality criteria. Baseline covariates were adjusted for baseline risk score, at risk status, and community of color status. Maximum failure event time was 126 days post D29 visit (D29 markers) or 100 days post D57 visit (D57 markers). FWER-adjusted P values were computed over the set of p-values both for quantitative markers and categorical markers (Low, Medium, High) using the Westfall and Young permutation method (10,000 replicates). All serological assay readouts assessed as immune correlates were first expressed in values relative to the WHO International Standard for anti-SARS-CoV-2 immunoglobulin (*27*): binding antibody readouts were converted to BAU/ml and pseudovirus neutralizing antibody titers as well as microneutralization assay readouts were calibrated to IU_{50}/ml or IU_{80}/ml. The shading represents, within each category, the antibody marker (out of all 10 for categories 1 and 2; out of the 3 evaluable ones for category 3) with the best performance.

	**Category 1: Correlate of Risk (CoR)**									**Category 2: Correlate of Protection (CoP): VE Modification**							**Category 3: CoP: VE Mediation**				
	HR per SD (Cox, quant.)		HR p-value (Cox, quant.)		HR High vs. Low tertile (Cox)		Hazard Ratio p-value tertile (Cox)		**CoR: Median rank**	Range of CVE Pt. Est. (Cox, 5th to 95th perc.)		Range of CVE Pt. Est. (NP, 5th to 95th percentile)		E-value Marg. Risk Ratio 95% UCL		**CoP: VE Mod Median rank**	Proportion Mediated				**CoP: VE Med Median rank**
	Pt. Est. (95% CI)	Rank	FWER	Rank	Pt. Est. (95% CI)	Rank	FWER	Rank		Pt. Est. (95% CI)	Rank	Pt. Est. (95% CI)	Rank	E-value	Rank		Pt. Est.	Rank	95% LCL	Rank	
D29 Spike IgG (BAU/ml)	0.73 (0.62, 0.86)	7	<0.001	1	0.19 (0.08, 0.44)	1	<0.001	1	**1**	6.6	7	16.9	7	4.5	1	**7**	--	--	--	--	--
D57 Spike IgG (BAU/ml)	0.85 (0.76, 0.95)	10	0.020	9	0.23 (0.09, 0.60)	4	0.020	5	**7**	2.7	10	21.9	2	3.1	4	**4**	--	--	--	--	--
D29 RBD IgG (BAU/ml)	0.68 (0.55, 0.83)	5	0.001	2	0.28 (0.13, 0.60)	5	0.005	4	**4.5**	9.3	3	14.9	8	3.0	5	**5**	--	--	--	--	--
D57 RBD IgG (BAU/ml)	0.80 (0.70, 0.92)	9	0.010	7	0.28 (0.12, 0.67)	5	0.021	6	**6.5**	4.0	9	23.9	1	2.6	6	**6**	--	--	--	--	--
D29 PsV-nAb ID_{50} (IU_{50}/ml)	0.55 (0.38, 0.79)	2	0.004	5	0.32 (0.15, 0.69)	8	0.001	2	**3.5**	17.7	1	18.7	3	2.5	7	**3**	69.9 (59.8, 80.0)	1	59.8	1	**1**
D57 PsV-nAb ID_{50} (IU_{50}/ml)	0.69 (0.57, 0.83)	6	0.002	3	0.31 (0.12, 0.80)	7	0.108	9	**6.5**	7.5	6	17.7	6	2.0	8	**6**	--	--	--	--	--
D29 PsV-nAb ID_{80} (IU_{80}/ml)	0.48 (0.30, 0.77)	1	0.006	6	0.22 (0.09, 0.51)	3	0.004	3	**3**	14.7	2	18.2	4	3.7	2	**2**	48.5 (35.0, 62.0)	2	35.0	2	**2**
D57 PsV-nAb ID_{80} (IU_{80}/ml)	0.67 (0.54, 0.83)	4	0.003	4	0.20 (0.07, 0.61)	2	0.025	8	**4**	8.4	5	17.8	5	3.3	3	**5**	--	--	--	--	--
D29 LV-MN_{50} (IU_{50}/ml)	0.62 (0.43, 0.91)	3	0.017	8	0.46 (0.21, 1.01)	9	0.021	6	**7**	9.3	3	11.5	9	1.2	9	**9**	29.2 (17.2, 41.2)	3	17.2	3	**3**
D57 LV-MN_{50} (IU_{50}/ml)	0.78 (0.59, 1.02)	8	0.108	10	0.78 (0.34, 1.77)	10	0.571	10	**10**	5	8	6.0	10	1.0	10	**10**	--	--	--	--	--

Table S4. Ratio of sample sizes required to power a future immunogenicity or immunobridging trial for the column D57 antibody marker compared to the row D57 antibody marker, calculated using the method described by Follmann (*18*). Confidence intervals are based on 10^3 bootstrap replicates.

Ratios

	D57 Spike IgG	D57 RBD IgG	D57 ID_{50}	D57 ID_{80}	D57 LV-MN_{50}
D57 Spike IgG	-	0.85	0.54	0.58	2.33
D57 RBD IgG	1.18	-	0.64	0.68	2.75
D57 ID_{50}	1.84	1.56	-	1.06	4.29
D57 ID_{80}	1.74	1.47	0.94	-	4.04
D57 LV-MN_{50}	0.43	0.36	0.23	0.25	-

Lower and upper bounds of the ratios

	D57 Spike IgG	D57 RBD IgG	D57 ID_{50}	D57 ID_{80}	D57 LV-MN_{50}
D57 Spike IgG	-	(0.56, 1.20)	(0.11, 1.82)	(0.13, 1.59)	(0.57, Inf)
D57 RBD IgG	(0.83, 1.79)	-	(0.15, 2.14)	(0.17, 1.80)	(0.69, Inf)
D57 ID_{50}	(0.55, 8.94)	(0.47, 6.77)	-	(0.67, 1.74)	(1.07, Inf)
D57 ID_{80}	(0.63, 7.77)	(0.56, 5.74)	(0.58, 1.49)	-	(1.15, Inf)
D57 LV-MN_{50}	(0.00, 1.76)	(0.00, 1.44)	(0.00, .93)	(0.00, .87)	-

Table S5. Ratio of sample sizes required to power a future immunogenicity or immunobridging trial for the column D29 antibody marker compared to the row D29 antibody marker, calculated using the method described by Follmann (*18*). Confidence intervals are based on 10^3 bootstrap replicates.

**Ratios**					
	D29 Spike IgG	D29 RBD IgG	D29 ID_{50}	D29 ID_{80}	D29 LV-MN_{50}
D29 Spike IgG	-	1.12	1.41	1.65	2.45
D29 RBD IgG	0.90	-	1.26	1.48	2.19
D29 ID_{50}	0.71	0.79	-	1.17	1.74
D29 ID_{80}	0.61	0.68	0.85	-	1.48
D29 LV-MN_{50}	0.41	0.46	0.58	0.68	-
**Lower and upper bounds of the ratios**					
	D29 Spike IgG	D29 RBD IgG	D29 ID_{50}	D29 ID_{80}	D29 LV-MN_{50}
D29 Spike IgG	-	(0.74, 2.59)	(0.53, 4.59)	(0.62, 5.10)	(0.89, 17)
D29 RBD IgG	(0.39, 1.35)	-	(0.36, 3.25)	(0.39, 3.68)	(0.59, 12)
D29 ID_{50}	(0.22, 1.88)	(0.31, 2.77)	-	(0.73, 1.70)	(0.55, 11)
D29 ID_{80}	(0.20, 1.62)	(0.27, 2.57)	(0.59, 1.37)	-	(0.51, 10)
D29 LV-MN_{50}	(0.06, 1.12)	(0.08, 1.70)	(0.09, 1.83)	(0.10, 1.95)	-

Table S6. Estimated hazard ratio of COVID-19 per standard deviation (SD) increase in D29 and D57 marker, restricting to post D57 cases, in SARS-CoV-2 baseline negative per-protocol vaccine recipients (exploratory analyses). *Two-phase sampling Cox model was adjusted for baseline risk score, at risk status, and communities of color status. All serological assay readouts assessed as immune correlates were first expressed in values relative to the WHO International Standard for anti-SARS-CoV-2 immunoglobulin (*27*): binding antibody readouts were converted to BAU/ml and pseudovirus neutralizing antibody titers as well as microneutralization assay readouts were calibrated to IU_{50}/ml or IU_{80}/ml. BAU, binding antibody units; CI, confidence interval; ID_{50}, 50% inhibitory dilution; ID_{80}, 80% inhibitory dilution; IU, international units; LV, live virus; MN_{50}, 50% microneutralization dilution; nAb, neutralizing antibody; PsV, pseudovirus; Pt. Est., point estimate; SD, standard deviation.

	**Hazard ratio* per SD increase in marker**	
**Antibody marker**	D29 markers<br>Pt. Est. (95% CI)	D57 markers<br>Pt. Est. (95% CI)
Spike IgG (BAU/ml)	0.65 (0.47, 0.89)	0.66 (0.50, 0.88)
RBD IgG (BAU/ml)	0.59 (0.36, 0.96)	0.57 (0.40, 0.82)
PsV-nAb ID_{50} (IU_{50}/ml)	0.45 (0.22, 0.92)	0.42 (0.27, 0.65)
PsV-nAb ID_{80} (IU_{80}/ml)	0.29 (0.09, 0.92)	0.35 (0.20, 0.61)
LV-MN_{50} (IU_{50}/ml)	0.63 (0.30, 1.33)	0.51 (0.25, 1.04)

Table S7. Estimated hazard ratio of COVID-19 per standard deviation (SD) increase in the marker, for antibody marker pairs in SARS-CoV-2 baseline negative per-protocol vaccine recipients (exploratory analyses). *Two-phase sampling Cox model was adjusted for baseline risk score, at risk status, and communities of color status. **P-value from generalized Wald test of null hypothesis that all assay marker variables have null association. All serological assay readouts assessed as immune correlates were first expressed in values relative to the WHO International Standard for anti-SARS-CoV-2 immunoglobulin (*28*): binding antibody readouts were converted to BAU/ml and pseudovirus neutralizing antibody titers as well as microneutralization assay readouts were calibrated to IU_{50}/ml or IU_{80}/ml. BAU, binding antibody units; CI, confidence interval; ID_{50}, 50% inhibitory dilution; ID_{80}, 80% inhibitory dilution; IU, international units; LV, live virus; MN_{50}, 50% microneutralization dilution; nAb, neutralizing antibody; PsV, pseudovirus; SD, standard deviation.

Marker	Estimated Hazard Ratio per SD-Increase in the Marker (95% CI)*		
**D29 Markers**			
D29 RBD IgG (BAU/ml)	--	0.91 (0.62, 1.34)	0.77 (0.58, 1.04)
D29 PsV-nAb ID_{50} (IU_{50}/ml)	0.60 (0.34, 1.07)	0.60 (0.34, 1.04)	--
D29 LV-MN_{50} (IU_{50}/ml)	0.89 (0.49, 1.60)	--	0.75 (0.47, 1.19)
P-value**	0.005	0.002	0.004
**D57 Markers**			
D57 RBD IgG (BAU/ml)	--	1.04 (0.70, 1.55)	0.82 (0.65, 1.04)
D57 PsV-nAb ID_{50} (IU_{50}/ml)	0.56 (0.37, 0.86)	0.66 (0.40, 1.08)	--
D57 LV-MN_{50} (IU_{50}/ml)	1.26 (0.78, 2.04)	--	0.95 (0.63, 1.43)
P-value**	<0.001	<0.001	0.008

Table S8. Predictors of symptomatic COVID-19 in baseline negative per-protocol vaccine recipients and their coefficients in the discrete SuperLearner learner (top-performing learner based on cross-validated risk) selected for a representative fold of cross-validation (out of the 5 folds) across all 34 variable sets. Results shown are for the first random seed (out of 10 random seeds). SL.glm_screen_all: generalized linear model fit without screening of variables; SL.glmnet.0_screen_all: Lasso model with alpha = 1 fit without screening of variables; risk score: detailed in supplementary text S1 of (*10*)]; HighRiskInd: Indicator that participant had at least one of the following risk factors: chronic lung disease (e.g., emphysema, chronic bronchitis, idiopathic pulmonary fibrosis, cystic fibrosis, or moderate-to-severe asthma); cardiac disease (e.g., heart failure, congenital coronary artery disease, cardiomyopathies, or pulmonary hypertension); severe obesity (body mass index≥40); diabetes (type 1, type 2, or gestational); liver disease; or HIV infection; MinorityInd: Indicator that subjects are Black or African American, Hispanic or Latino, American Indian or Alaska Native, Native Hawaiian, and other Pacific Islander; Day29bindSpike: IgG Spike concentration at D29; Day57pseudoneutid50: PsV-nAb ID_{50} titer at D57; Day29pseudoneutid80: PsV-nAb ID_{80} titer at D29; Day57pseudoneutid80: PsV-nAb ID_{58} titer at D57; Delta29overBpseudoneutid80_2fold: Indicator that D29 PsV-ID_{80} titer is at least 2 times larger than D1 PsV-nAb ID_{80} titer; Delta29overBpseudoneutid50_4fold: Indicator that D29 PsV-nAb ID_{50} titer is at least 4 times larger than D1 PsV-nAb ID_{50} titer; Day57liveneutmn50: WT LV-MN_{50} titer at D57; Delta57overBliveneutmn50_4fold: Indicator that D57 WT LV-MN_{50} titer is at least 4 times larger than D1 WT LV-MN_{50} titer; comb_nlPCA1_d57_d29: First component of non-linear principal component analysis (PCA) as combination score across all five markers at D29 and D57; Coefficient: log odds ratio in a logistic regression model (glm) or an L1-penalized logistic regression model (glmnet). CI, confidence interval; ID_{50}, 50% inhibitory dilution; ID_{80}, 80% inhibitory dilution; IU, international units; LV, live virus; MN_{50}, 50% microneutralization dilution; nAb, neutralizing antibody; PsV, pseudovirus.

Variable Set	Learner	Predictors/Features	Coefficient	Odds Ratio
1 Baseline Risk Factors	SL.glm_screen_all	(Intercept)	-5.874	0.0028
		risk_score	0.433	1.542
		HighRiskInd	0.328	1.388
		MinorityInd	-0.209	0.811
2 Baseline Risk Factors + IgG Spike, RBD markers at D29, D57	SL.glmnet.0_screen_all	(Intercept)	-5.686	0.0034
		risk_score	0.170	1.185
		HighRiskInd	0.174	1.190
		MinorityInd	-0.031	0.969
		Day57bindSpike	-0.090	0.914
3 Baseline Risk Factors + Pseudovirus neutralization ID_{50}, ID_{80} markers at D29, D57	SL.glm_screen_all	(Intercept)	-6.086	0.0023
		risk_score	0.690	1.993

		HighRiskInd	0.275	1.317
		MinorityInd	-0.071	0.932
		Day57pseudoneutid80	-0.329	0.720
		Day29pseudoneutid80	-0.048	0.953
		Delta29overBpseudoneutid80_2fold	-0.395	0.674
4 Baseline Risk Factors + Wild-Type Live virus neutralization ID_{50}, ID_{80} markers at D29, D57	SL.glmnet.0_screen_all	(Intercept)	-5.991	0.003
		risk_score	0.556	1.744
		HighRiskInd	0.231	1.259
		MinorityInd	-0.17	0.844
		Day57liveneutmn50	-0.334	0.716
		Delta57overBliveneutmn50_4fold	0.202	1.224
5 Baseline Risk Factors + All Markers at D29, D57 _	SL.glmnet.0_screen_all	(Intercept)	-6.038	0.0024
		risk_score	0.547	1.728
		HighRiskInd	0.181	1.198
		MinorityInd	-0.098	0.906
		Day57pseudoneutid50	-0.140	0.870
		Day57pseudoneutid80	-0.086	0.918
		Delta29overBpseudoneutid50_4fold	-0.071	0.932
		Delta29overBpseudoneutid80_2fold	-0.274	0.760
		comb_nlPCA1_d57_d29	0.0398	1.041

Table S9. Immune assay limits. All serological assay readouts assessed as immune correlates were first expressed in values relative to the WHO International Standard for anti-SARS-CoV-2 immunoglobulin (*28*): binding antibody readouts were converted to BAU/ml and pseudovirus neutralizing antibody titers as well as microneutralization assay readouts were calibrated to IU_{50}/ml or IU_{80}/ml. [1]LV-MN_{50} values below the LoD were assigned the value LoD/2. Values between the LoD and the LLOQ were taken as their actual numeric value. Values greater than the ULOQ were assigned the value of the ULOQ. [2] PsV-nAb ID_{50} and PsV-nAb ID_{80} values below the LoD were assigned the value LoD/2. Values between the LoD and the LLOQ were taken as their actual numeric value. For immunogenicity reporting, values greater than the ULOQ were not given a ceiling value of the ULOQ, the actual readouts were used. For the immune correlates analyses, values greater than the ULOQ were assigned the value of the ULOQ. [3] Antibody response was defined by detectable IgG concentration above the antigen-specific positivity cut-off (10.8424 BAU/ml for spike, 14.0858 BAU/ml for RBD). BAU, binding antibody units; CI, confidence interval; ID_{50}, 50% inhibitory dilution; ID_{80}, 80% inhibitory dilution; IU, international units; LoD, limit of detection; LV, live virus; MN_{50}, 50% microneutralization dilution; nAb, neutralizing antibody; PsV, pseudovirus; ULOQ, upper limit of quantitation.

	Live Virus Microneutralization Assay (LV-MN_{50}) (Battelle) (IU_{50}/ml)[1]	Pseudovirus Neutralization Assay (PsV-nAb) (Duke) (IU_{50}/ml)[2]	Pseudovirus Neutralization Assay (PsV-nAb) (Duke) (IU_{80}/ml)[2]	Binding Antibody Assay (VRC) (BAU/ml)[3]	Binding Antibody Assay (VRC) (BAU/ml)[3]
	LV-MN_{50}	PsV-nAb ID_{50}	PsV-nAb ID_{80}	Spike	RBD
Positivity Cutoff	22.66	2.42	15.02	10.8424	14.0858
LoD	22.66	2.42	15.02	0.3076	1.5936
LLOQ	44.1	4.477	21.4786	1.7968	3.43
ULOQ	3083.74	10919	15368	10,155.95	16,269

Table S10. The 13 learner-screen combinations inputted into superlearner.

Learner	Screen
SL.mean	All
SL.glmnet.0	All
SL.glmnet.1	All
SL.xgboost.2.no	All
SL.xgboost.4.no	All
SL.xgboost.2.yes	All
SL.xgboost.4.yes	All
SL.ranger.yes	All
SL.ranger.no	All
SL.glm	All
SL.glm	glmnet
SL.glm	univar_logistic_pval
SL.glm	highcor_random

Table S11. 34 variable sets for fitting superlearner to predict COVID-19 in baseline negative per-protocol vaccine recipients. Baseline risk factors are baseline risk score, at-risk status, and community of color status. bAb, binding antibody; BAU, binding antibody units; ID_{50}, 50% inhibitory dilution; ID_{80}, 80% inhibitory dilution; IU, international units; LoD, limit of detection; LV, live virus; MN_{50}, 50% microneutralization dilution; nAb, neutralizing antibody; PCA, principal components analysis; PsV, pseudovirus; ULOQ, upper limit of quantitation.

Variable Set Name	Variables included in the set
1_baselineRiskFactors	Baseline risk factors only (Reference model)
2_bAbSpike_D57	Baseline risk factors + D57 bAb anti-Spike markers
3_bAbRBD_D57	Baseline risk factors + D57 bAb anti-RBD markers
4_pnabID50_D57	Baseline risk factors + D57 p-nAb ID_{50} markers
5_pnabID80_D57	Baseline risk factors + D57 p-nAb ID_{80} markers
6_lnabMN50_D57	Baseline risk factors + D57 l-nAb MN_{50} markers
7_bAb_pnabID50_D57	Baseline risk factors + D57 bAb markers and p-nAb ID_{50} markers
8_bAb_pnabID80_D57	Baseline risk factors + D57 bAb markers and p-nAb ID_{80} markers
9_bAb_lnabMN50_D57	Baseline risk factors + D57 bAb markers and l-nAb MN_{50} markers
10_bAb_combScores_D57	Baseline risk factors + D57 bAb markers and combination scores across the five markers [PCA1, PCA2, FSDAM1/FSDAM2 (the first two components of nonlinear PCA), and the maximum signal diversity score]
11_allMarkers_D57	Baseline risk factors + all individual D57 marker variables
12_allMarkers_combScores_D57	Baseline risk factors + all individual D57 marker variables and their combination scores (Full model of D57 markers)
13_bAbSpike_D29	Baseline risk factors + D29 bAb anti-Spike markers
14_bAbRBD_D29	Baseline risk factors + D29 bAb anti-RBD markers
15_pnabID50_D29	Baseline risk factors + D29 p-nAb ID_{50} markers
16_pnabID80_D29	Baseline risk factors + D29 p-nAb ID_{80} markers
17_lnabMN50_D29	Baseline risk factors + D29 l-nAb MN_{50} markers
18_bAb_pnabID50_D29	Baseline risk factors + D29 bAb markers and p-nAb ID_{50} markers
19_bAb_pnabID80_D29	Baseline risk factors + D29 bAb markers and p-nAb ID_{80} markers
20_bAb_lnabMN50_D29	Baseline risk factors + D29 bAb markers and l-nAb MN_{50} markers
21_bAb_combScores_D29	Baseline risk factors + D29 bAb markers and combination scores across the five markers [PCA1, PCA2, FSDAM1/FSDAM2 (the first two components of nonlinear PCA), and the maximum signal diversity score]
22_allMarkers_D29	Baseline risk factors + all individual D29 marker variables
23_allMarkers_combScores_D29	Baseline risk factors + all individual D29 marker variables and their combination scores (Full model of D29 markers)
24_bAbSpike_D29_D57	Baseline risk factors + D29 and D57 bAb anti-Spike markers
25_bAbRBD_D29_D57	Baseline risk factors + D29 and D57 bAb anti-RBD markers
26_pnabID50_D29_D57	Baseline risk factors + D29 and D57 p-nAb ID_{50} markers
27_pnabID80_D29_D57	Baseline risk factors + D29 and D57 p-nAb ID_{80} markers
28_lnabMN50_D29_D57	Baseline risk factors + D29 and D57 l-nAb MN_{50} markers
29_bAb_pnabID50_D29_D57	Baseline risk factors + D29 and D57 bAb markers and p-nAb ID_{50} markers

30_bAb_pnabID80_D29_D57	Baseline risk factors + D29 and D57 bAb markers and p-nAb ID_{80} markers
31_bAb_lnabMN50_D29_D57	Baseline risk factors + D29 and D57 bAb markers and l-nAb MN_{50} markers
32_bAb_combScores_D29_D57	Baseline risk factors + D29 and D57 bAb markers and combination scores across the ten markers [PCA1, PCA2, FSDAM1/FSDAM2 (the first two components of nonlinear PCA), and the maximum signal diversity score]
33_allMarkers_D29_D57	Baseline risk factors + all individual D29 and D57 marker variables
34_allMarkers_combScores_D29_D57	Baseline risk factors + all individual D29 and D57 marker variables and their combination scores (Full model of D29 and D57 markers)

Data file S1. Statistical analysis plan.
