# Supplementary material for: Comparing antibody assays as correlates of protection against COVID-19 in the COVE mRNA-1273 vaccine efficacy trial
Source: Sci Transl Med. Author manuscript; Available in PMC 2023 Jun 6. (PMC10243212; doi:10.1126/scitranslmed.ade9078)
Supplement: Suppl MDAR Reproducibility [file NIHMS1899084-supplement-Suppl_MDAR_Reproducibility.pdf]

Materials Design Analysis Reporting (MDAR) Checklist for Authors

The MDAR framework establishes a minimum set of requirements in transparent reporting applicable to studies in the life sciences (see Statement of Task: doi:10.31222/osf.io/9sm4x.). The MDAR checklist is a tool for authors, editors, and others seeking to adopt the MDAR framework for transparent reporting in manuscripts and other outputs. Please refer to the MDAR Elaboration Document for additional context for the MDAR framework.

For all that apply, please note where in the manuscript the required information is provided.

Materials:

Newly created materials	indicate where provided: page no/section/legend)	n/a
The manuscript includes a dedicated "materials availability statement" providing transparent disclosure about availability of newly created materials including details on how materials can be accessed and describing any restrictions on access.	On Page 39 (one of the notes after the References), in section "Data and materials availability"	

Antibodies	indicate where provided: page no/section/legend)	n/a
For commercial reagents, provide supplier name, catalogue number and RRID, if available.	Methods -> Live SARS-CoV-2 Virus Neutralizing Antibody Assay" p. 23-25:<br>• 2019-nCoV/USA-WA1/2020; GenBank Accession Number MN985325.1; CDC provided<br>• Anti-nucleocapsid protein primary antibody cocktail (clones HM1056 and HM1057) (EastCoast Bio); catalog no. HM1056 and HM1057.<br>• secondary antibody (goat anti-mouse IgG Horse Radish Peroxidase (HRP) conjugate; Fitzgerald; catalog no. 43C-CB1569	

DNA and RNA sequences	indicate where provided: page no/section/legend)	n/a
**Short novel DNA or RNA including primers, probes:** Sequences should be included or deposited in a public repository.	N/A	N/A

Cell materials	indicate where provided: page no/section/legend	n/a
**Cell lines:** Provide species information, strain. Provide accession number in repository **OR** supplier name, catalog number, clone number, **OR** RRID.	Methods -> Live SARS-CoV-2 Virus Neutralizing Antibody Assay" p. 23:<br>• Vero-E6 cells (African green monkey kidney, passage 31; originally obtained from BEI Resources [Catalog No. NR-596]	
**Primary cultures:** Provide species, strain, sex of origin, genetic modification status.		N/A

Experimental animals	indicate where provided: page no/section/legend)	n/a
**Laboratory animals or Model organisms:** Provide species, strain, sex, age, genetic modification status. Provide accession number in repository **OR** supplier name, catalog number, clone number, **OR** RRID.		N/A
**Animal observed in or captured from the field:** Provide species, sex, and age where possible.		N/A

Plants and microbes	indicate where provided: page no/section/legend)	n/a
**Plants:** provide species and strain, ecotype and cultivar where relevant, unique accession number if available, and source (including location for collected wild specimens).		N/A

**Microbes:** provide species and strain, unique accession number if available, and source.	Methods -> Live SARS-CoV-2 Virus Neutralizing Antibody Assay" p. 23-24:<br>"The SARS-CoV-2 stock was produced by infecting VERO E6 cells (African green monkey kidney, passage 31; originally obtained from BEI Resources [Catalog No. NR-596]) with CDC provided material (2019-nCoV/USA-WA1/2020; GenBank Accession Number MN985325.1; passage 3) at multiplicity of infection of 0.001 in Eagle's Minimum Essential Medium supplemented with antibiotics and 2% fetal bovine serum. Virus-containing supernatant was harvested following 72 hours of incubation at 37°C ± 2°C and 5% ± 2% CO2, pooled, clarified by centrifugation, aliquoted, and stored at ≤ -70°C."	

**Human research participants**	**indicate where provided: page no/section/legend) or state if these demographics were not collected**	**n/a**
If collected and within the bounds of privacy constraints report on age, sex and gender or ethnicity for all study participants.	Demographics and clinical characteristics of baseline SARS-CoV-2 negative per-protocol trial participants in the immunogenicity subcohort and thus have Day 1, 29, 57 antibody marker data were reported in table S1 in Gilbert et al. Science 2022 (reference *10* in the manuscript). This is referenced on p. 6 in Results "Immunogenicity subcohort, case-cohort sets, and COVID-19 endpoints".	

Design:

Study protocol	indicate where provided: page no/section/legend)	n/a
If study protocol has been pre-registered, provide DOI. For clinical trials, provide the trial registration number **OR** cite DOI.	Added in Introduction, bottom of p. 4.	

Laboratory protocol	indicate where provided: page no/section/legend)	n/a
Provide DOI **OR** other citation details if detailed step-by-step protocols are available.		N/A

Experimental study design (statistics details)		
**For in vivo studies:** State whether and how the following have been done	**indicate where provided: page no/section/legend. If it could have been done, but was not, write not done**	**n/a**
Sample size determination	The second paragraph of the “Study Design” section in Materials and Methods (p. 22) states that the minimum number of COVID-19 endpoint cases in the vaccine arm required for each immune correlate analysis is provided in Table 7 of the SAP.	
Randomisation	The last paragraph of the “Study Design” section in Materials and Methods (p. 22) states that participants were randomly sampled for measurement of antibody markers on D1, D29, and D57. The case-cohort sampling design for this is detailed in Gilbert et al., which is cited there (ref *10*).	
Blinding	The second paragraph of the “Study Design” section in Materials and Methods (p. 21-22) states that all laboratory staff conducting the immune assays were blinded to group allocation during data collection and analysis.	
Inclusion/exclusion criteria	The third paragraph of the “Study Design” section in Materials and Methods (p. 22) states that “Correlates analyses were conducted for baseline-negative per-protocol participants defined in (10), i.e. participants with no immunologic or virologic evidence of prior COVID-19 at enrollment [as in (16)] who received both doses without major protocol violations.”	

Sample definition and in-laboratory replication	indicate where provided: page no/section/legend	n/a
State number of times the experiment was replicated in laboratory.	Methods -> Live SARS-CoV-2 Virus Neutralizing Antibody Assay” p. 24: “Each sample was tested independently in singlet by one operator on one test plate following standard operator procedures. The same sample was then tested by a second operator in singlet on a different plate on the same day. If necessary, repeat testing of any samples were performed in singlet by one operator on a different test day. The final reportable value for each sample was the median LV-MN50 titer of a minimum of two passing independent results.” This information is also in the legend of Figure 2.	
Define whether data describe technical or biological replicates.	Methods -> Live SARS-CoV-2 Virus Neutralizing Antibody Assay” p. 24: “Each sample was tested independently in singlet by one operator on one test plate following standard operator procedures. The same sample was then tested by a second operator in singlet on a different	

	plate on the same day. If necessary, repeat testing of any samples were performed in singlet by one operator on a different test day. The final reportable value for each sample was the median LV-MN50 titer of a minimum of two passing independent results." This information is also in the legend of Figure 2.	

Ethics	indicate where provided: page no/section/legend	n/a
**Studies involving human participants:** State details of authority granting ethics approval (IRB or equivalent committee(s), provide reference number for approval.	Materials and Methods, Study Design (p. 22-23): "The Institutional IRB approval number for the use of human sera in the pseudovirus neutralization assay is Pro00105358 (DUHS Institutional Review Board, 2424 Erwin Rd, Durham, NC, 919.668.5111, Federalwide Assurance No: FWA 00009025 Suite 405). The human specimens for Battelle's analysis were collected from human volunteers in accordance with the requirements of ModernaTX IRB of record (Advarra IRB; Clinical Trial NCT04470427). All human specimens received by Battelle were coded. Biospecimens were not identifiable to Battelle nor did Battelle possess any code-key or way to associate results of analysis with the original human donors. Further, there was no intention to try to identity or otherwise attribute any results of analysis to the original human donors. As such, this study did not meet regulatory criteria for categorization as human subjects research for the Battelle-specific scope of work and Battelle is not considered to be engaged in research according to DHHS published guidance. This opinion for the use of human sera in the microneutralization assay is identified as IRB HSRE 389-0100142771. The opinion was provided on behalf of the Battelle Institutional Review Board: Federalwide Assurance FWA00004696, IRB Registration Number IRB0000284."	
**Studies involving experimental animals:** State details of authority granting ethics approval (IRB or equivalent committee(s), provide reference number for approval.		N/A
**Studies involving specimen and field samples:** State if relevant permits obtained, provide details of authority approving study; if none were required, explain why.		N/A

Dual Use Research of Concern (DURC)	indicate where provided: page no/section/legend	n/a
If study is subject to dual use research of concern regulations, state the authority granting approval and reference number for the regulatory approval.		N/A

Analysis:

**Attrition**	**indicate where provided: page no/section/legend**	**n/a**
Describe whether exclusion criteria were preestablished. Report if sample or data points were omitted from analysis. If yes report if this was due to attrition or intentional exclusion and provide justification.	See the Statistical Analysis Plan, provided as Supplementary Material: See Sections 3.1 "Study Cohort for Correlates Analyses", 3.2 "Study Endpoints", 5.1 "Immunogenicity subcohort", 16 "Avoiding Bias with Pseudovirus Neutralization Analysis due to Use of Anti-HIV Antiretroviral Drugs".	

**Statistics**	**indicate where provided: page no/section/legend**	**n/a**
Describe statistical tests used and justify choice of tests.	Materials and Methods -> Statistical Analysis (p. 29): "All p-values are two-sided. For each set of hypothesis tests, q-values and FWER p-values (family-wise error rate adjusted p-values) were computed over the set of p-values (separately for D29 and for D57 marker correlates of risk) both for quantitative markers and categorical markers (considering all five antibody markers: spike IgG, RBD IgG, PsV-nAb ID50, PsV-nAb ID80, and LV-MN50) using the Westfall and Young (34) permutation method (10000 replicates)."	

**Data availability**	**indicate where provided: page no/section/legend**	**n/a**
For newly created and reused datasets, the manuscript includes a data availability statement that provides details for access or notes restrictions on access.	"Data and materials availability" statement on p. 39.	
If newly created datasets are publicly available, provide accession number in repository **OR** DOI **OR** URL and licensing details where available.		N/A
If reused data is publicly available provide accession number in repository **OR** DOI **OR** URL, **OR** citation.		N/A

**Code availability**	**indicate where provided: page no/section/legend**	**n/a**
For all newly generated custom computer code/software/mathematical algorithm or re-used code essential for replicating the main findings of the study, the manuscript includes a data availability statement that provides details for access or notes restrictions.	"Data and materials availability" statement on p. 39.	
If newly generated code is publicly available, provide accession number in repository, **OR** DOI **OR** URL and licensing details where available. State any restrictions on code availability or accessibility.	"Data and materials availability" statement on p. 39.<br>Ref #34 contains the DOI: doi: 10.5281/zenodo.7510753.	
If reused code is publicly available provide accession number in repository **OR** DOI **OR** URL, **OR** citation.		N/A

Reporting

MDAR framework recommends adoption of discipline-specific guidelines, established and endorsed through community initiatives. Journals have their own policy about requiring specific guidelines and recommendations to complement MDAR.

Adherence to community standards	indicate where provided: page no/section/legend	n/a
State if relevant guidelines (e.g., ICMJE, MIBBI, ARRIVE) have been followed, and whether a checklist (e.g., CONSORT, PRISMA, ARRIVE) is provided with the manuscript.	p. 38: "ICMJE guidelines for authorship have been adhered to."	
